# Remyelination promoting therapies in multiple sclerosis animal models: a systematic review and meta-analysis

**DOI:** 10.1038/s41598-018-35734-4

**Published:** 2019-01-29

**Authors:** Carlijn R. Hooijmans, Martin Hlavica, Florian A. F. Schuler, Nicolas Good, Andrin Good, Lisa Baumgartner, Gianluca Galeno, Marc P. Schneider, Tarzis Jung, Rob de Vries, Benjamin V. Ineichen

**Affiliations:** 1 Brain Research Institute, University of Zurich and Department of Health Sciences and Technology, ETH Zurich, 8057 Zurich, Switzerland; 2 Systematic Review Centre for Laboratory Animal Experimentation (SYRCLE), Department of Health Evidence, Radboud University Medical Center, Nijmegen, The Netherlands; 3 Institute of Neuroinformatics, University of Zurich and ETH Zurich, Zurich, Switzerland; 4 Waidspital Zurich, Department of Radiology, Tiechestrasse 99, 8037 Zurich, Switzerland; 5 Present Address: Department of Neurology, Inselspital, Bern University Hospital, University of Bern, Bern, Switzerland

## Abstract

An unmet but urgent medical need is the development of myelin repair promoting therapies for Multiple Sclerosis (MS). Many such therapies have been pre-clinically tested using different models of toxic demyelination such as cuprizone, ethidium bromide, or lysolecithin and some of the therapies already entered clinical trials. However, keeping track on all these possible new therapies and their efficacy has become difficult with the increasing number of studies. In this study, we aimed at summarizing the current evidence on such therapies through a systematic review and at providing an estimate of the effects of tested interventions by a meta-analysis. We show that 88 different therapies have been pre-clinically tested for remyelination. 25 of them (28%) entered clinical trials. Our meta-analysis also identifies 16 promising therapies which did not enter a clinical trial for MS so far, among them Pigment epithelium-derived factor, Plateled derived growth factor, and Tocopherol derivate TFA-12.We also show that failure in bench to bedside translation from certain therapies may in part be attributable to poor study quality. By addressing these problems, clinical translation might be smoother and possibly animal numbers could be reduced.

## Introduction

Multiple Sclerosis (MS) is a chronic demyelinating disease^1^. With the only exception of Ocrelizumab^2^, which has a modest impact on disease progression, none of the 16 FDA approved MS therapies are able to stop or at least to decelerate the progressively increasing disability of affected patients^3^. One well-acknowledged approach to prevent disease progression is via a boost of myelin repair. Remyelination not only restores efficient electric conduction along axons but, because the myelin sheaths have trophic functions for the axons^4^, also reduces neurodegeneration, which closely correlates with clinical disability^5^.

The intense search for strategies to enhance myelin regeneration to hinder further neurodegeneration and to increase clinical function of patients, is as well reflected by the large number of pre-clinical studies assessing potential remyelinating strategies. Commonly used experimental systems to study potential remyelinating therapies include neuro-inflammatory animal models such as experimental autoimmune encephalomyelitis (EAE)^6^ or virus-induced demyelination/inflammation^7^ as well as toxin-induced demyelination models with cuprizone^8^, lysolecithin, ethidium bromide, and complement/anti-galactocerebroside antibodies^9^ being the most commonly used agents of the latter group^10^. All of these models have strengths and limitations; whereas neuro-inflammatory models reproduce well the disseminated and inflammatory features of MS, toxin-induced demyelination models are more suited to dissect specific mechanisms of myelin decline and regeneration with a clear temporal separation of these processes and without concomitant inflammation^10,11^.

Cuprizone is a systemic copper-chelating agent. Upon feeding, it leads to demyelination of distinct brain regions, among them the corpus callosum. Cuprizone has a highly reproducible timeline of de- and remyelination and enables long-term demyelination when fed for a prolonged time window. The exact mechanism of cuprizone-induced demyelination is unknown^10,12^. Compared to Cuprizone, lysolecithin has the disadvantage of needing an invasive injection to a pre-defined CNS-position. Nevertheless, it is also highly predictive under temporal aspects. Moreover, any CNS area can be targeted selectively with this detergent^13^. Ethidium bromide leads to much larger areas of demyelination and degrades all nucleated cells within the injection area (including astrocytes and microglia cells)^9^. The local injection of Anti-galactocerebroside antibodies/complement is rarely used as toxic demyelination model. The time to complete remyelination is shorter in this model but involves a greater demyelinating area than lysolecithin^10^.

Many putative therapies have been identified using these toxic demyelination models, from which some already entered clinical trials. We aimed at summarizing all the already pre-clinically tested putative remyelinating therapies via a systematic review and meta-analysis in order to assess which remyelinating therapies can be promising and could be tested in clinical trials. We also investigate the efficacy of the therapies in these  experimental animal models that have already entered clinical trials. We focused our analysis on the four *in vivo* toxic demyelination models cuprizone, lysolecithin, ethidium bromide, and complement/anti-galactocerebroside antibodies. These models might be more suited to assess potential therapies aiming at halting disease progression of progressive MS in contrast to neuro-inflammatory models, in which potential immune-modulatory effects of therapies could confound efficacy^11^. The results of our review should provide a framework for future clinical trials investigating putative remyelinating interventions for MS, in particular during the chronic phase of the disease when remyelination failure determines disability progression.

## Results

### Study characteristics

#### Study selection process

**Figure 1:**
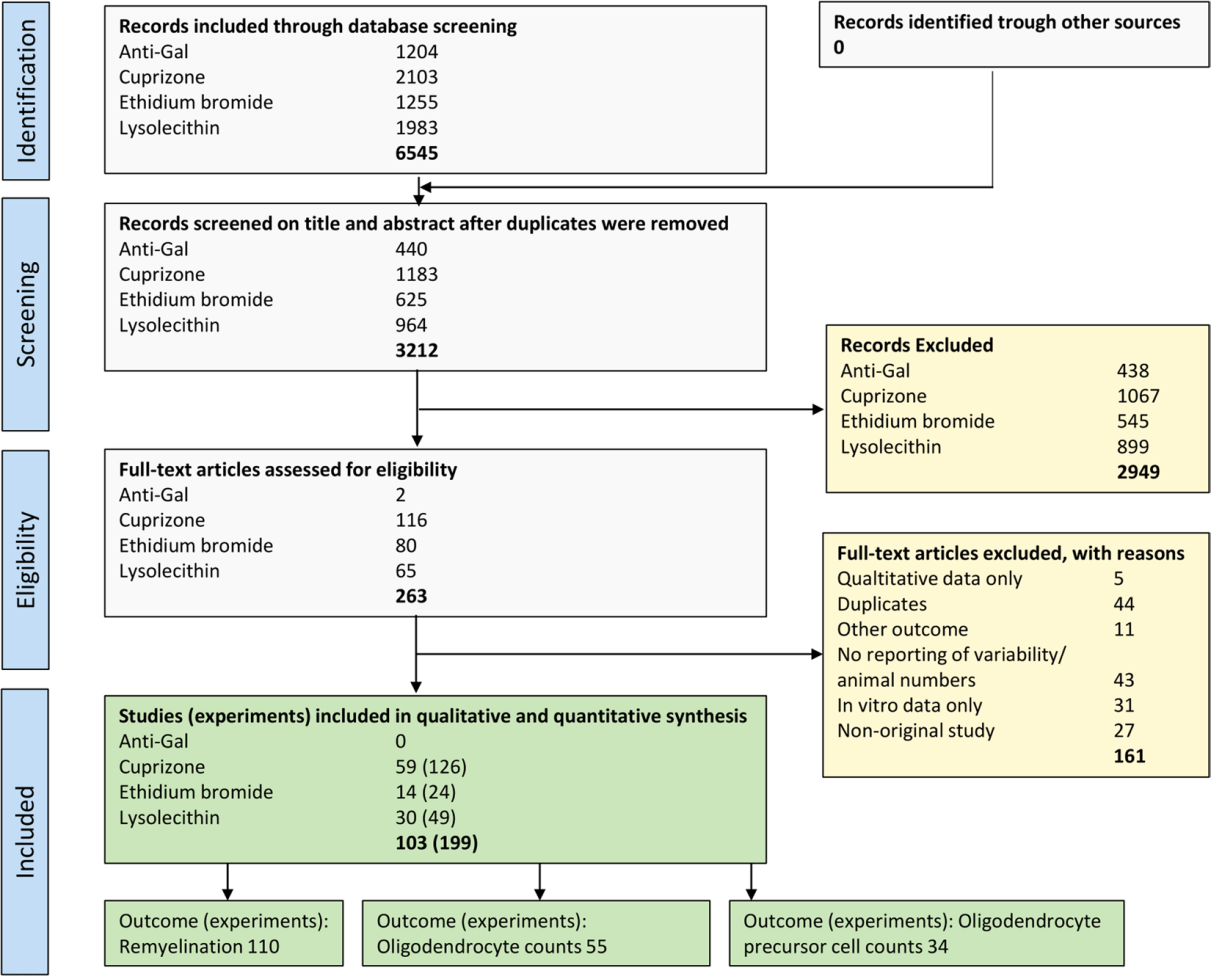
Quorum chart of the study selection process^14^. Duplicate references are references that are mentioned in multiple medical databases (e.g. same references in Embase and Pubmed) or studies published in duplicate (e.g. same study in multiple journals).

Figure 1 depicts the Quorum flow chart of the study selection process^14^. Using 4 different search strings for anti-galactocerebroside antibodies, cuprizone, ethidium bromide, and lysolecithin, respectively (see Supplementary Information), a total of 6545 publications were retrieved from EMBASE, go3R, Medline, Pubmed, Scopus, and Web of Sciences. After initial screening of titles and abstracts, 263 publications were included for full-text search. Out of these, 103 studies met our inclusion criteria (see supplementary reference list). The remainder were excluded according to the criteria listed in Fig. 1.

#### Publication over time

**Figure 2:**
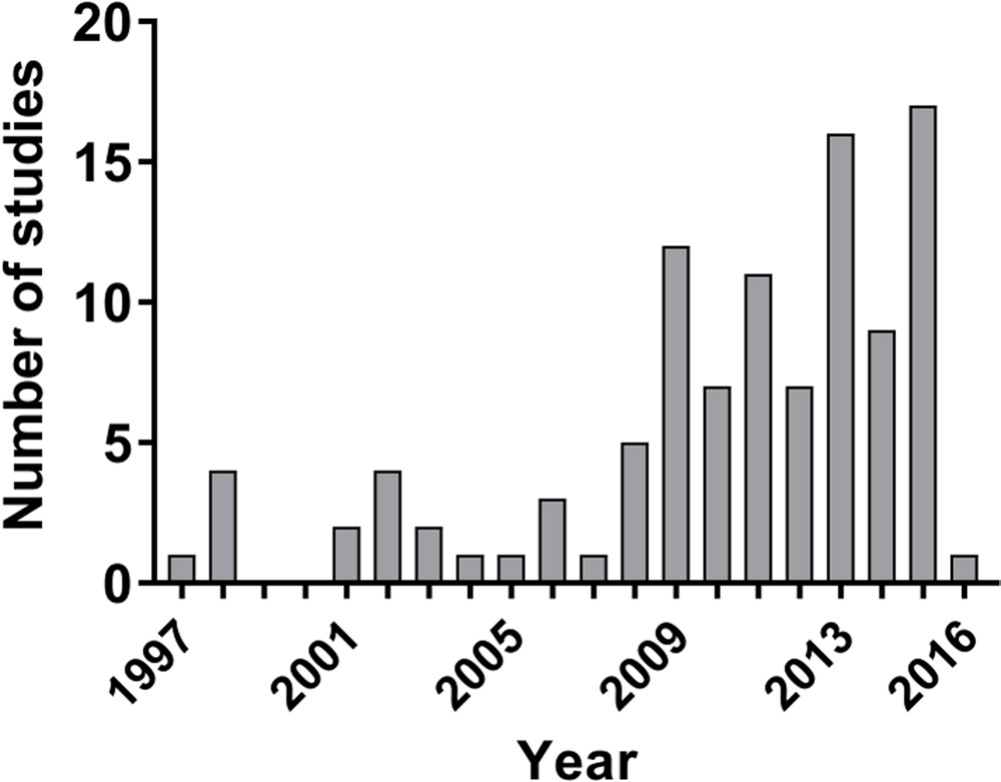
Histogram depicting the number of included studies per year.

The first reports using models of toxic demyelination *in vivo* were published in the seventies. Lysolecithin was the first model being published in 1971^15^, followed by cuprizone in 1972^16^, ethidium bromide in early 1979^17^, and anti-galactocerebroside antibodies in mid 1979^18^. The first reports using these models to assess a therapy for its remyelinating potential *in vivo* and meeting our inclusion criteria, however, was only in 1997^19^. Thus, all studies included in the meta-analysis were published between 1997 and 2016 (Fig. 2).

#### Description of the included studies

**Figure 3:**
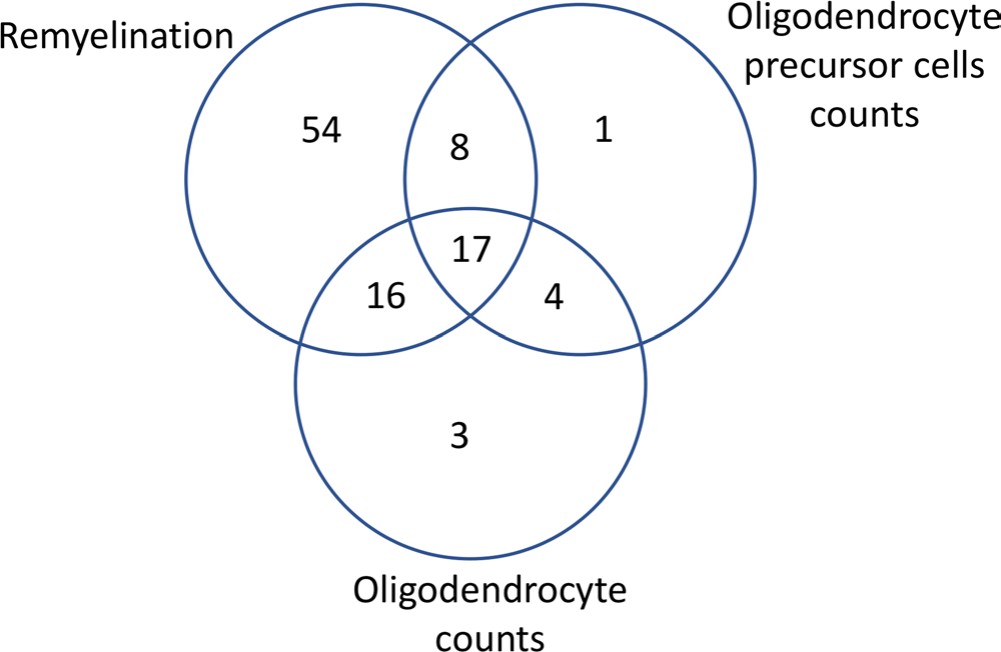
Venn diagram illustrating how many of the 103 studies assessed one or more of the outcomes remyelination, oligodendrocyte precursor (OPC) count, and oligodendrocyte count.

The characteristics of the 103 included studies are shown in Supplementary Table 1. A total of 88 different therapies were tested in the included studies. 59 studies used cuprizone (58%), 30 studies used lysolecithin (29%), 14 studies used ethidium bromide (13%), and none of the included studies used anti-galactocerebroside antibodies as demyelination model. Only two different animal species have been used in the included studies: mice (72 studies, 70%) and rats (31 studies, 30%). Most studies used male animals (47, 45%), whereas females were less commonly used (28, 27%); 25 studies (24%) did not report which sex they used, and 3 studies (3%) combined female and male animals within the same experimental groups. Most of the studies used remyelination as an outcome measure (95, 93%), 54 of them (52%) investigated remyelination as the single outcome without using oligodendrocyte or OPC counts as outcome. 51 used oligodendrocyte cell counts (50%), and 33 used oligodendrocyte precursor cells (OPC) counts as outcome (32%). Nine studies (9%) did not directly assess remyelination but assessed oligodendrocyte or OPC counts only. Seventeen studies (17%) assessed all three outcome measures remyelination, oligodendrocyte, and OPC count **(**Fig. 3).

#### Study quality and risk of bias

**Figure 4:**
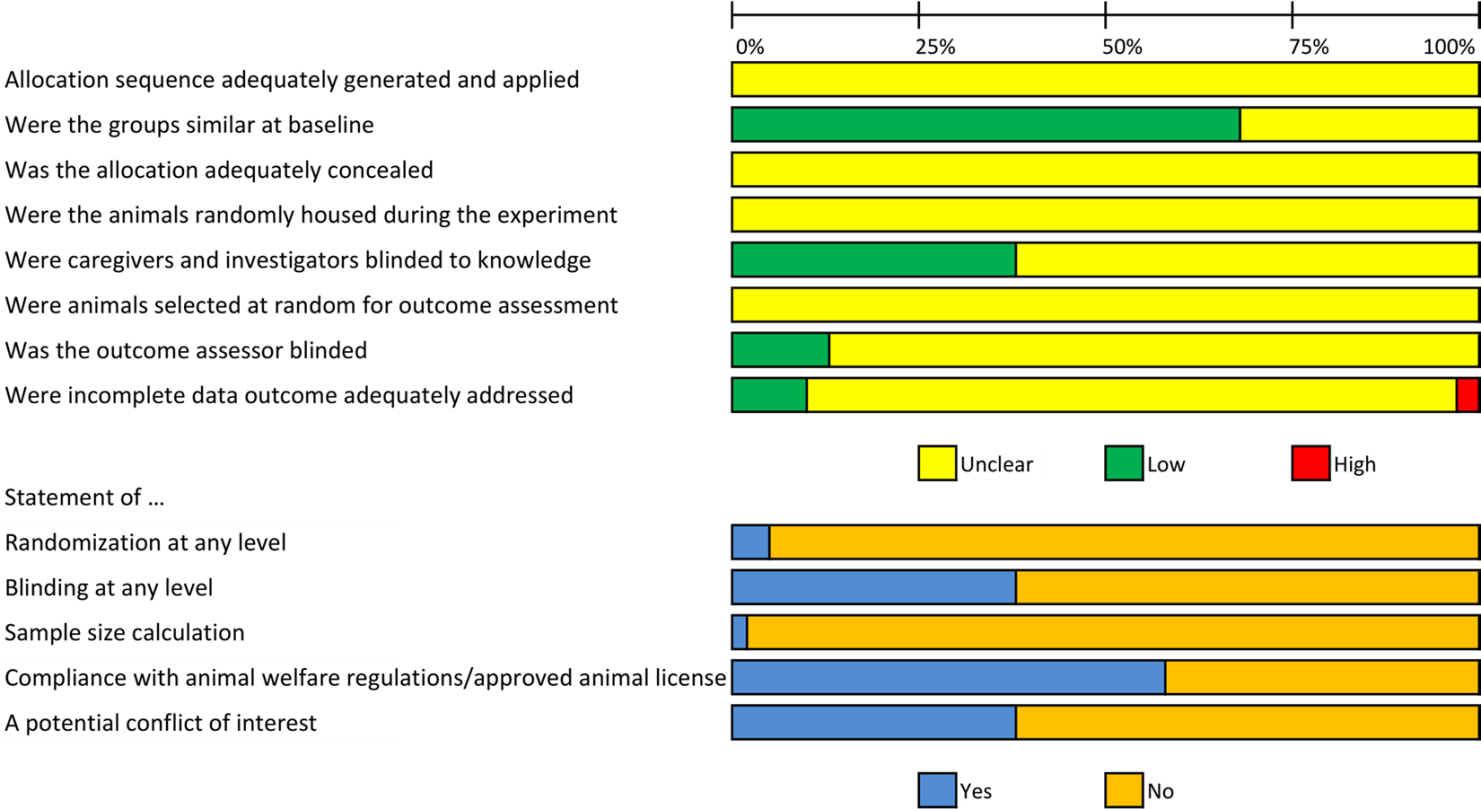
Risk of bias assessment of the included studies. The first 8 items assess study quality by reporting “yes” indicating low risk of bias, “no” high risk of bias, and “unclear” as not reported^20^. The remaining 5 items score a “yes” if reported and a “no” if not reported.

Figure 4 depicts the risk of bias assessment for the 103 included studies. For many items of the adapted SYRCLE risk of bias tool^20^, poor reporting regarding experimental details led overall to an unclear risk of bias. Assessment of selection bias showed that none of the included studies described whether or not they blinded the allocation of the animals to the experimental or groups or described the methods used for allocation sequence generation. Although only 5% of the papers reported any measure used to randomise the experiment, fortunately 68% of publications reported similar experimental groups at baseline. Regarding the risk of performance bias, none of the publications reported whether the animals were randomly housed across cages and rooms during the experiment. 38% of the publications reported that staff was blinded during the experiments. Concerning the risk of detection bias, none of the publications reported on random outcome assessment whereas in 13% of the studies, the outcome assessor had been blinded for animal allocation. Measures to reduce attrition bias were reported in 10% of publications. There was a high risk of attrition bias in 3%. In 87%, the risk of attrition bias was unclear as a consequence of poor reporting of the number of experimental animals used in both methods and result sections of the papers. Sample sizes appeared small in most of the included studies, although only 2% of the included studies reported a a prior sample size calculation. The median number of animals in the treatment or control group was between 5 and 5.5 (Table 1).

**Table 1:** Sample sizes of the included studies, Abbreviations: SD, standard deviation; OPC, oligodendrocyte precursor cells.

Outcome	Median/mean number (±SD) of animals in treatment group	Median/mean number (±SD) of animals in control group	Range
Remyelination	5.5/6.7 (±3.7)	5/6.1 (±3.7)	2–21
Oligodendrocyte cell counts	5/6.5 (±3.7)	5/6.1 (±3.5)	3–18
OPC counts	5/6.5 (±3.8)	5/6.4 (±3.7)	3–21

Because poor reporting in preclinical studies is a known problem and therefore many items of the risk of bias tool are scored as unclear risk of bias, we additionally scored two reporting items. These items were also scored in a similar study in EAE^21^ and in focal cerebral ischemia (FCI)^22^: Five percent of studies reported randomization at any level (For comparison - EAE: 9%, FCI: 36%). Blinding of the experiment at any level was reported in 38% of studies (For comparison - EAE: 16%, FCI: 29%).

Finally, 2 studies (1.8%) reported a prior sample size calculation (EAE: <1%, FCI: 3%) whereas 2 studies (1.8%) reported no prior sample size calculation. The remaining publications did not mention whether a sample size calculation has been performed. Compliance with animal welfare regulations or an approved animal license were reported in 58% of cases (For comparison - EAE: 32%, FCI: 57%). A statement whether a conflict of interest was present was reported in 38% of studies (For comparison - EAE: 6%, FCI: 23%).

### Meta-analysis

#### Remyelination as outcome

In the studies measuring remyelination, different methods were used in the 110 comparisons: most investigators used electron microscopy as outcome (32 experiments, 29%), followed by myelin basic protein (MBP) immunohistofluorescence/-chemistry (26 experiments, 24%), Luxol Fast Blue (LFB) staining (17 experiments, 15%), and Toluidine blue staining on semithin sections (11 experiments, 10%). In contrast, Black Gold staining (8 experiments, 7%), Hematoxylin and Eosin staining (5 experiments, 5%), PLP immunohistofluorescence (4 experiments, 4%), Eriochrome staining (3 experiments, 3%), MOG immunohistochemistry (2 experiments, 2%), MRI lesion load (1 experiment, 1%), and MRI magnetization transfer (1 experiment, 1%) were less commonly used.

Pooling the individual effect sizes of all therapies in our meta analyses showed that the therapies described in literature enhance remyelination (Standardized mean difference (SMD): 1.61, 95% CI: [1.26, 1.97], Table 2). The overall heterogeneity^23^ between the studies, however, was high (I2 = 85%), reflecting the anticipated differences between interventions, models used and study design.

**Table 2:** subgroup analysis of the included studies for outcome remyelination.

Subgroup	Comparisons	Effect estimate [95% CI]	I^2^
**Overall**	**110**	**1.61** [**1.26, 1.97]**	**85%**
**MS model**			
Cuprizone	66	1.61 [1.16, 2.07]	83%
Ethidium bromide	16	1.67 [0.68, 2.65]	87%
Lysolecithin	28	1.60 [0.90, 2.30]	86%
**Method**			
Electron microscopy	32	1.73 [1.05, 2.40]	87%
MBP immuno-staining	26	2.16 [1.44, 2.87]	82%
Luxol fast blue	17	1.85 [0.97, 2.73]	79%
H&E lesion area	5	5.17 [2.83, 7.51]	92%
MOG immune-staining	2	3.29 [0.23, 6.35]	85%
**Therapeutic regimen**			
therapeutically	102	1.67 [1.30, 2.04]	84%

The effect estimate was displayed by the standardized mean difference (SMD) and 95% confidence intervals (CIs). No significant differences were found in the subgroup analyses. Abbreviations: MBP, myelin basic protein; MOG, myelin oligodendrocyte glycoprotein.

**Figure 5:**
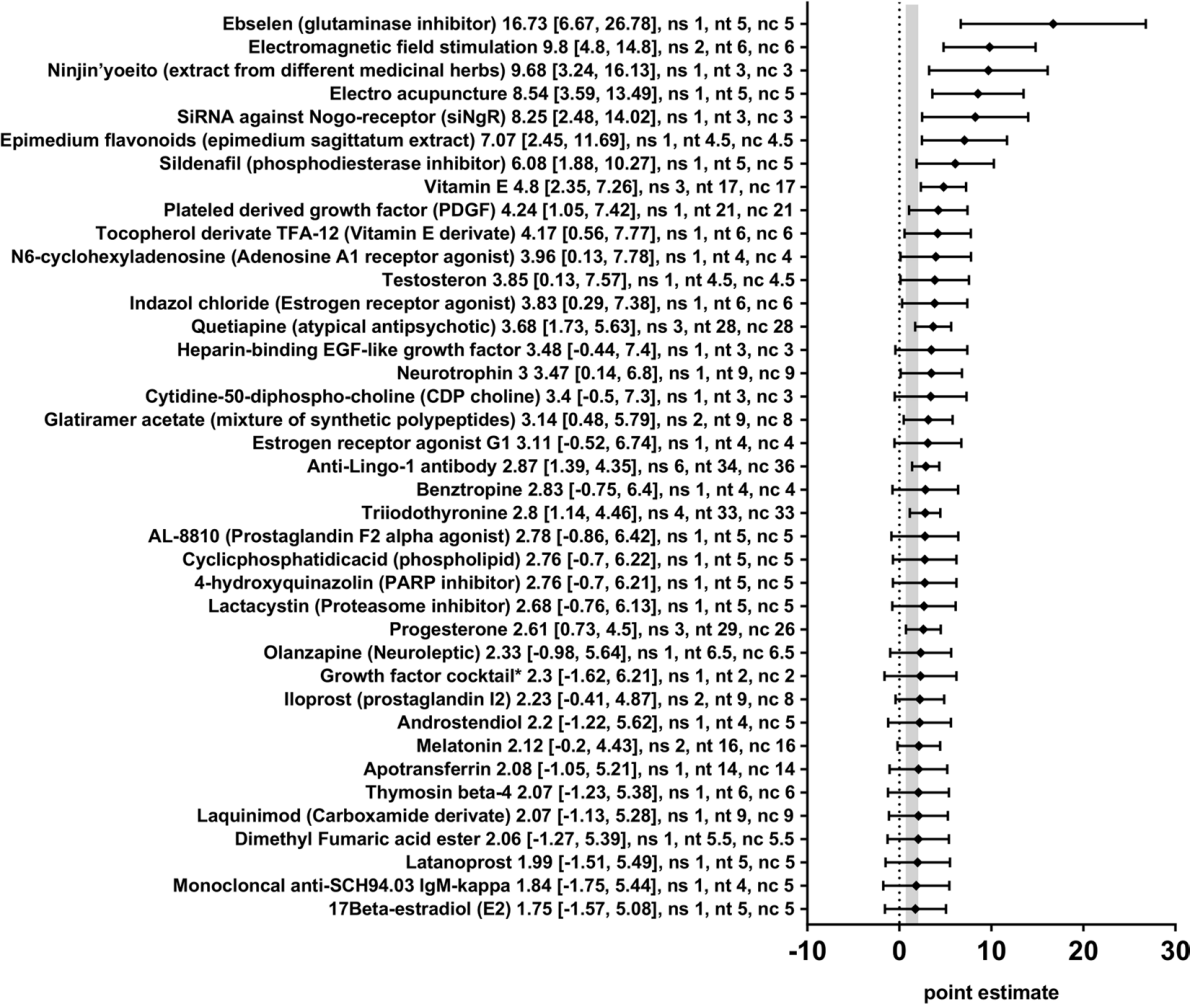
Forest plot of the included studies for the outcome remyelination (part 1). The diamond indicates the global estimate and its 95% confidence interval (CI). The numbers listed after a certain intervention are: the exact effect size with its 95% CI, the number of included studies for a certain intervention (ns), the total number of treated animals (nt) or control animals (nc). The gray bar indicates the 95% CI of the overall effect size. References are given in the Supplementary Information. *The growth factor cocktail contained Platelet derived growth factor-AA, neurotrophin-3, basic fibroblast growth factor, and insulin-like growth factor-1.

**Figure 6:**
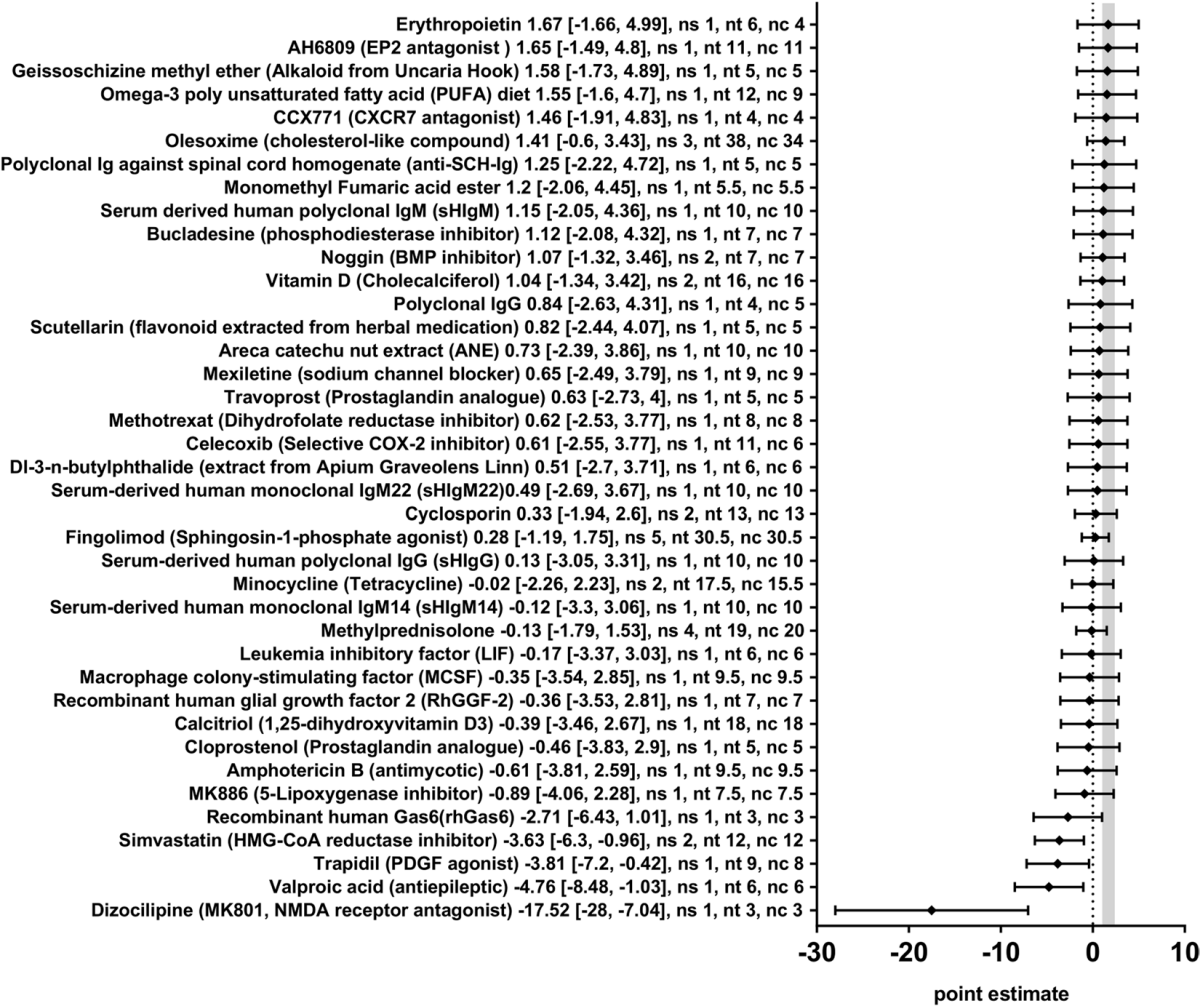
Forest plot of the included studies for the outcome remyelination (part 2). The diamond indicates the global estimate and its 95% confidence interval (CI). The numbers listed after a certain intervention are: the exact effect size with its 95% CI, the number of included studies for a certain intervention (ns), the total number of treated animals (nt) or control animals (nc). The gray bar indicates the 95% CI of the overall effect size. References are given in the Supplementary Information.

In order to obtain a more detailed overview of the efficacy of the various therapies included in this review, we also analysed the effect of the seventy-eight different therapeutic approaches in 110 different comparisons for their remyelination potential as measured by different morphometric approaches (i.e. the quantification of thinly myelinated axons in electron microscopy or by similar methods) separately. Nineteen therapeutic approaches significantly enhanced remyelination (Anti-Lingo-1-antibody, Ebselen, Electro acupuncture, Electromagnetic field stimulation, Epimedium flavonoids, Glatiramer acetate, Indazol chloride, N6-cyclohexyladenosine, Neurotrophin 3, Ninjińyoeito, Plateled-derived growth factor, Progesterone, Quetiapine, Sildenafil, SiRNA against Nogo-receptor, Testosterone, Tocopherol derivate TFA12, Triiodothyronine, and Vitamin E) and four therapies dampened the remyelination after experimental demyelination (Dizocilipine, Simvastatin, Trapidil, and Valproic acid) (Figs 5 and 6). For the remaining 55 therapeutic approaches, no statistically significant results were found. In many cases, only one or a few studies were available per therapeutic approach.

Further subgroup analyses for timing of the intervention (e.g. prophylactical or therapeutical administration) or type of model used revealed no differences. Due to the small number of comparisons in the subgroups, these results must be interpreted with caution.

#### Oligodendrocyte precursor cell (OPC) count as outcome

In the studies visualizing OPCs, different methods were used in the 34 different comparisons: NG2 was the most commonly used marker for OPCs (19 experiments, 56%), followed by PDGFRα (13 experiments, 38%). In contrast, Nkx2.2 (2 experiments, 6%) was less commonly used.

Pooling the individual effect sizes (SMDs) of all 34 experiments investigating the effect of various therapies on OPC counts did not show a significant effect (SMD: 0.87, 95% CI: [−0.08, 1.83], Table 3). The overall heterogeneity was very high (I2 = 92%).

**Table 3:** subgroup analysis of the included studies for outcome oligodendrocyte precursor cell counts.

Subgroup	Comparisons	Effect estimate [95% CI]	I^2^
**Overall**	**34**	**0.87** [**−0.08, 1.83]**	**92%**
**Therapeutic regimen**			
therapeutically	29	1.31 [0.25, 2.37]	93%
**Method**			
PDGFRα	13	2.50 [0.51, 4.49]	94%

The effect estimate was displayed by the standardized mean difference (SMD) and 95% confidence intervals (CIs). No significant differences were found in the subgroup analyses.

**Figure 7:**
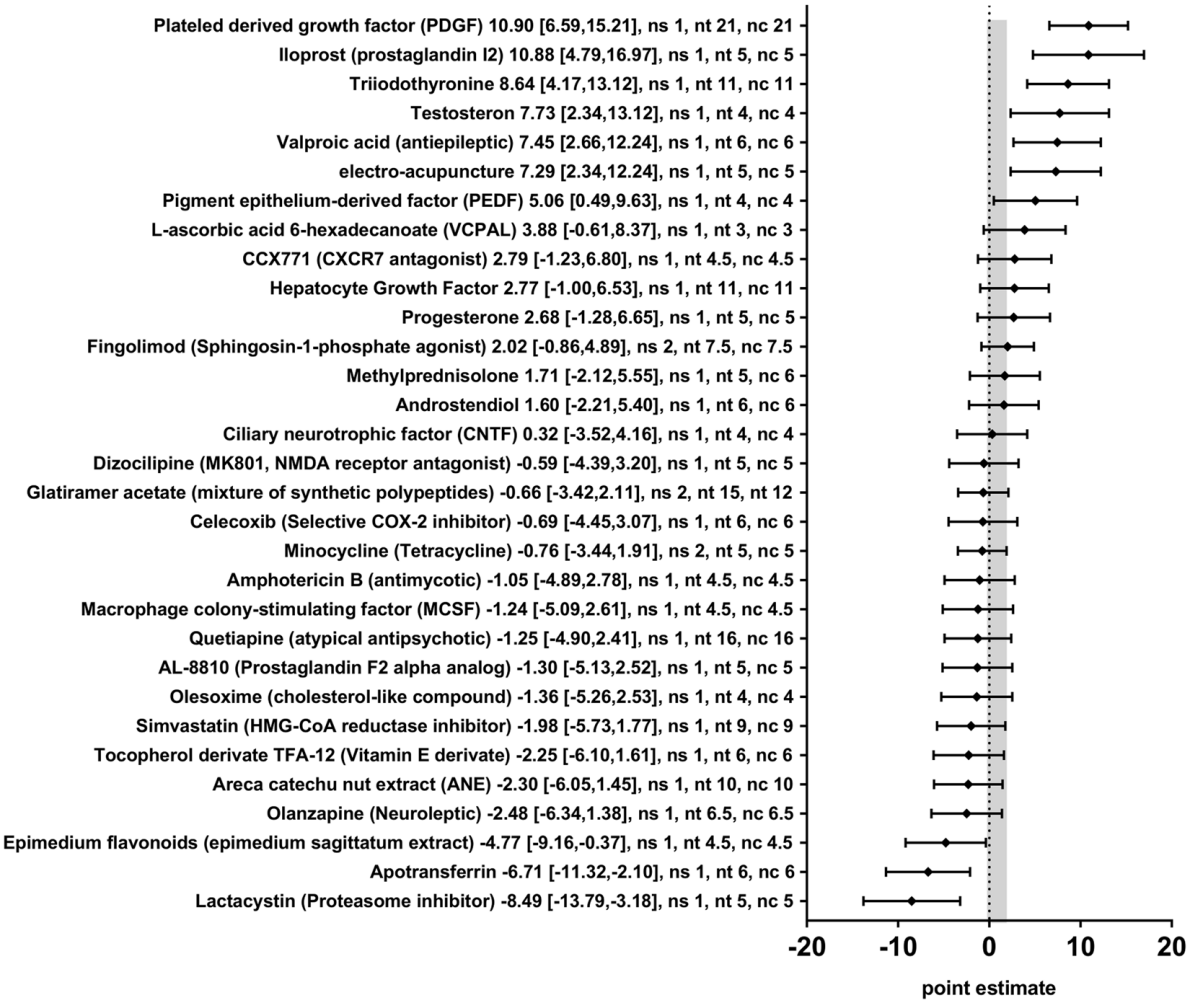
Forest plot of the included studies for outcome oligodendrocyte precursor cell (OPC) count. The diamond indicates the global estimate and its 95% confidence interval (CI). The gray bar indicates the 95% CI of the overall effect size. The numbers listed after a certain intervention are: the exact effect size with its 95% CI, the number of included studies for a certain intervention (ns), the total number of treated animals (nt) or control animals (nc). References are given in the Supplementary Information.

Thirty-one different therapeutic approaches in 34 different comparisons were tested for their impact on OPC counts as measured by cell counts of different immune-staining methods (i.e. NG2, PDGFRα, or Nkx2.2). The effectsizes (SMDs) and 95% CIs indicated that 7 therapeutic approaches significantly increased the OPC count (Electro acupuncture, Iloprost, Pigment epithelium-derived factor (PEDF), Platelet-derived growth factor (PDGF), Testosterone, Triiodothyronine, and Valproic acid) and 3 therapies significantly decreased the OPC count after experimental demyelination (Apotransferrin, Epimedium flavonoids, and Lactacystin) (Fig. 7). For the remaining 21 therapeutic approaches, no statistically significant effects were found. In many cases, only one or a few studies were available per therapeutic approach. No statistically significant differences between subgroups could be identified for type of model used or timing of the intervention (the subgroup of prophylactic administration was too small).

#### Oligodendrocytes as outcome

In the studies visualizing oligodendrocytes, different methods were used in the 55 comparisons: APC (CC1) was the most commonly used marker for oligodendrocytes (20 experiments, 36%), followed by GST-pi (11 experiments, 20%), and Nogo-A (9 experiments, 16%). In contrast, CA2 (5 experiments, 9%), CNP (4 experiments, 7%), O4 (3 experiments, 5%), and Olig2/APC (CC1) double staining (3 experiments, 5%), were used less commonly.

Meta analyses showed that the assessed therapies can increase oligodendrocyte cell counts (SMD: 2.09, 95% CI: [1. 52, 2.66], Table 4). The overall heterogeneity, however, was high (I2 = 87%), reflecting the anticipated differences between interventions, models used and study design.

**Table 4:** Subgroup analysis of the included studies for outcome oligodendrocytes cell counts.

Subgroup	Comparisons	Effect estimate [95% CI]	I^2^
**Overall**	**55**	**2.09** [**1.52, 2.66]**	**87%**
**Therapeutic regimen**			
therapeutically	49	2.10 [1.48, 2.71]	86%
**Method**			
APC*	20	2.43 [1.48, 3.38]	88%
GST-pi	11	2.14 [0.91, 3.37]	83%
Nogo-A*	9	0.12 [−1.16, 1.36]	72%
CA2	5	3.64 [1.71, 5. 57]	83%
O4	3	4.86 [2.31, 7.41]	96%
Olig2/APC	3	2.51 [0.13, 4.89]	48%
**MS model**			
Cuprizone***	42	1.77 [1.12, 2.41]	86%
Lysolecithin***	10	3.71 [2.27, 5.15]	91%

The effect estimate was displayed by the standardized mean difference (SMD) and 95% confidence intervals (CIs). *Significant subgroup analysis between Nogo-A and APC (adjusted p = 0.049), and Cuprizone and Lysolecithin.

**Figure 8:**
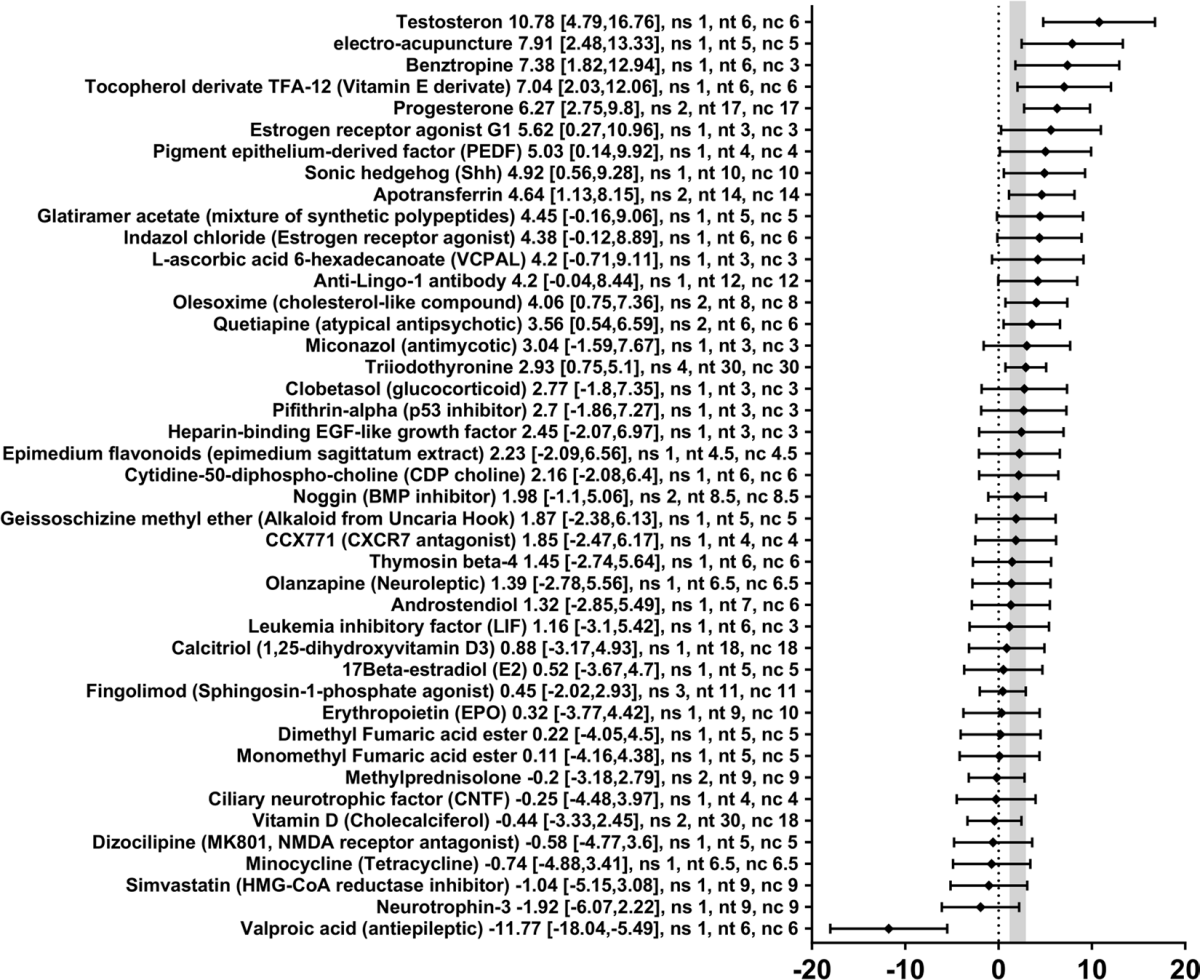
Forest plot of the included studies for outcome oligodendrocyte count. The diamond indicates the global estimate and its 95% confidence interval (CI). The gray bar indicates the 95% CI of the overall effect size. The numbers listed after a certain intervention are: the exact effect size with its 95% CI, the number of included studies for a certain intervention (ns), the total number of treated animals (nt) or control animals (nc). References are given in the Supplementary Information.

Subsequently, we investigated the effect of each therapy separately on oligodendrocytes cell counts. Forty-three different therapies in 55 different comparisons were tested for their impact on oligodendrocyte cell counts as measured by cell counts of different immune-staining methods (e.g. APC, CA2, Nogo-A, or others). The SMDs and 95% CIs indicated that 12 therapies significantly increased the oligodendrocyte cell counts (Apotransferrin, Benztropine, Electro acupuncture, Estrogen receptor agonist G1, Olesoxime, Pigment epithelium derived factor, Progesterone, Quetiapine, Sonic hedgehog, Testosterone, Tocopherol derivate TFA-12, and Triiodothyronine) and one therapy significantly decreased the oligodendrocyte cell counts after experimental demyelination (valproic acid, SMD −11.77, 95%-CI [−18.04, −5.49], 1 comparison) (Fig. 8). For the remaining 30 therapeutic approaches, no statistically significant results were found.

The number of independent comparisons per therapeutic approach were very low and varied between 1 and 4. No differences between subgroups were found for timing of the intervention. However, subgroup analyses for type of model used revealed that the type of model used to measure oligodendrocytes cell counts partly explains the observed heterogeneity between the studies. use of the LPC model significantly yielded more oligodendrocyte cell counts compared to the cuprizone model (p < 0.001).

### Publication bias

**Figure 9:**
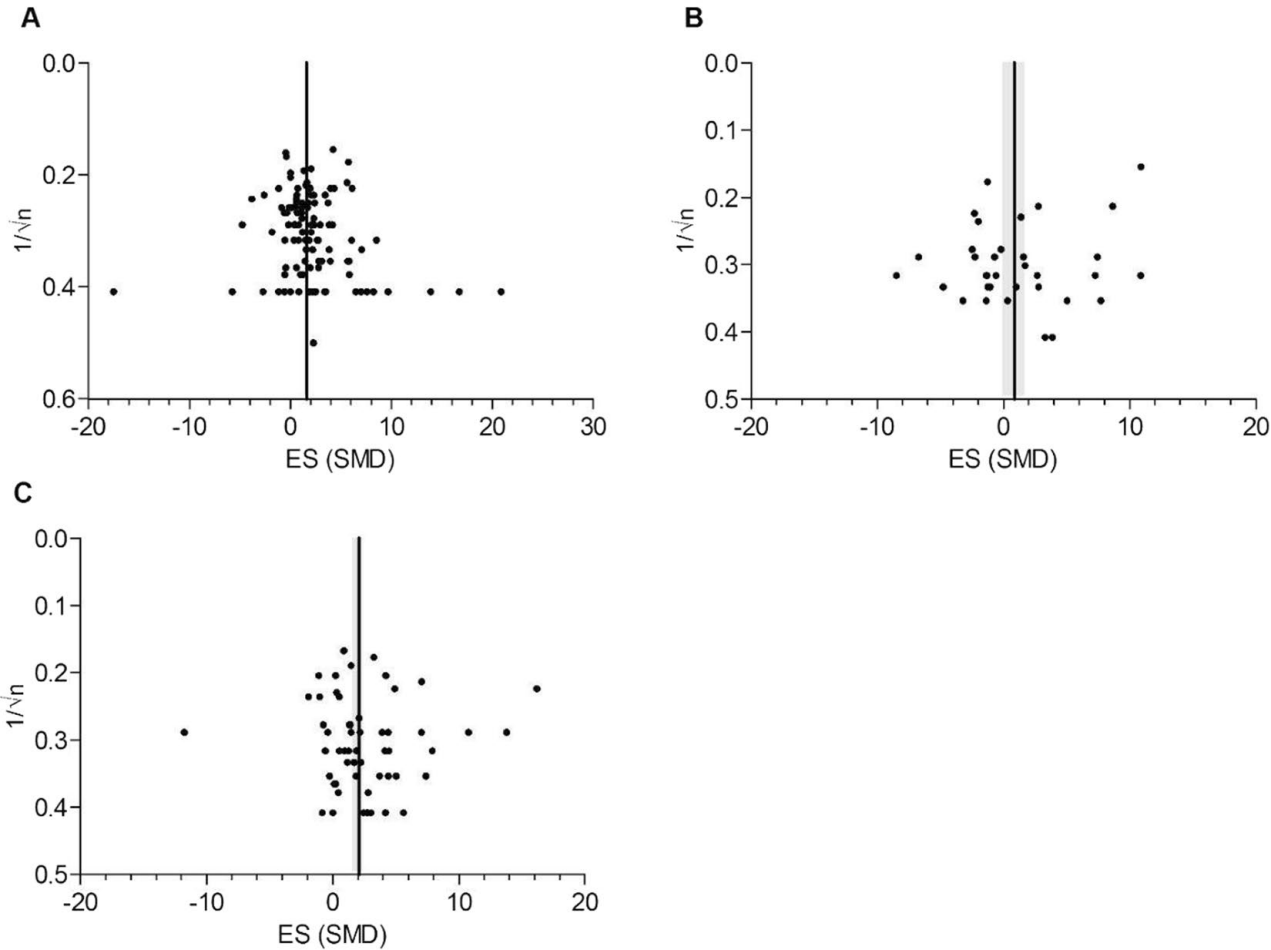
Evaluation of publication bias. Panel A, B and C represent the funnel plots for the outcomes remyelination, oligodendrocyte precursor cells (OPC) and oligodendrocyte count. The line represents the SMD of the summary effect, the grey bands represent global 95% confidence intervals.

Both visual inspection of the funnel plot and Egger regression did not suggest the presence of publication bias for remyelination, OPCs, and oligodendrocyte data. The funnel plots show no significant asymmetry, and no trimming could be performed (Fig. 9). Egger regression also showed no evidence for small study effects (remyelination p = 0.153, OPCs p = 0.063, oligodendrocytes p = 0.99).

### Synopsis of the results

Table 5 compares the therapies with a significant result in the meta-analysis and their impact on the outcomes remyelination, OPC count, and oligodendrocyte count and indicates whether a therapy has already entered a clinical trial. Most of the therapies that entered a clinical trial showed a beneficial effect in at least one of the three outcomes, except for Simvastatin (2 comparisons for remyelination, 1 for each OPC and oligodendrocyte counts). Simvastatin significantly decreased remyelination in experimental animal models and showed no effect on either OPC and oligodendrocyte numbers, but is already being tested in a phase 4 clinical trial for progressive MS.

**Table 5:** Summary of meta-analysis results for beneficial/harmful therapies on the outcomes remyelination, oligodendrocyte precursor cell (OPC) count, and oligodendrocyte count.

Therapy	remyelination	OPCs	Oligodendrocytes	Tested in clinical trial
Anti-Lingo-1-antibodies	Increase (6)	Not tested	Not significant (1)	Yes (II)
Apotransferrin	Not significant (1)	Decrease (1)	Increase (2)	No
Benztropine	Not significant (1)	Not tested	Increase (1)	No
Dizocilipine (MK801, MNDA receptor antagonist)	Decrease (1)	Not significant (1)	Not significant (1)	No
Ebselen (glutaminase inhibitor)	Increase (1)	Not tested	Not tested	Yes (II)
Electro acupuncture	Increase (1)	Increase (1)	Increase (1)	Yes (II)
Electromagnetic field stimulation	Increase (2)	Not tested	Not tested	Yes (II)
Epimedium flavonoids (extract of epimedium sagittatum)	Increase (1)	Decrease (1)	Not significant (1)	No
Estrogen receptor agonist G1	Not significant (1)	Not tested	Increase (1)	No
Glatiramer Acetate (mixture of synthetic polypeptides)	Increase (2)	Not significant (2)	Not significant (1)	Yes (IV)
Iloprost (prostaglandin I2)	Not significant (2)	Increase (1)	Not tested	No
Indazol chloride (Estrogen receptor agonist)	Increase (1)	Not tested	Not significant (1)	No
Lactacystin (Proteasome inhibitor)	Not significant (1)	Decrease (1)	Not tested	No
N6-cyclohexyladenosine (Adenosine A1-receptor agonist)	Increase (1)	Not tested	Not tested	No
Neurotrophin 3	Increase (1)	Not tested	Not significant (1)	No
Ninjin’yoeito (extract from different medicinal herbs)	Increase (1)	Not tested	Not tested	No
Olesoxime (cholesterol-like compound)	Not significant (3)	Not significant (1)	Increase (2)	Yes (I)
Pigment epithelium-derived factor (PEDF)	Not tested	Increase (1)	Increase (1)	No
Plateled derived growth factor (PDGF)	Increase (1)	Increase (1)	Not tested	No
Progesterone	Increase (3)	Not significant (1)	Increase (2)	Yes (III)
Quetiapine (atypical antipsychotic)	Increase (3)	Not significant (1)	Increase (2)	Yes (II)
Sildenafil (phosphodiesterase inhibitor)	Increase (1)	Not tested	Not tested	No
Simvastatin (HMG-CoA reductase inhibitor)	Decrease (2)	Not significant (1)	Not significant (1)	Yes (IV)
siRNA against Nogo-receptor	Increase (1)	Not tested	Not tested	No
Sonic hedgehog	Not tested	Not tested	Increase (1)	No
Testosterone	Increase (1)	Increase (1)	Increase (1)	Yes (II)
Tocopherol derivate TFA-12 (Vitamin E derivate)	Increase (1)	Not significant (1)	Increase (1)	No
Trapidil (PDGF agonist)	Decrease (1)	Not tested	Not tested	No
Triiodothyronine	Increase (4)	Increase (1)	Increase (4)	Yes (I)
Valproic acid (antiepileptic)	Decrease (1)	Increase (1)	Decrease (1)	No
Vitamin E	Increase (3)	Not tested	Not tested	Yes (II)

The numers in brackets in the second to fourth columns indicate the number of comparisons for each outcome. The roman number in brackets in the last column indicates the latest phase of clinical trial the therapy has entered.

**Figure 10:**
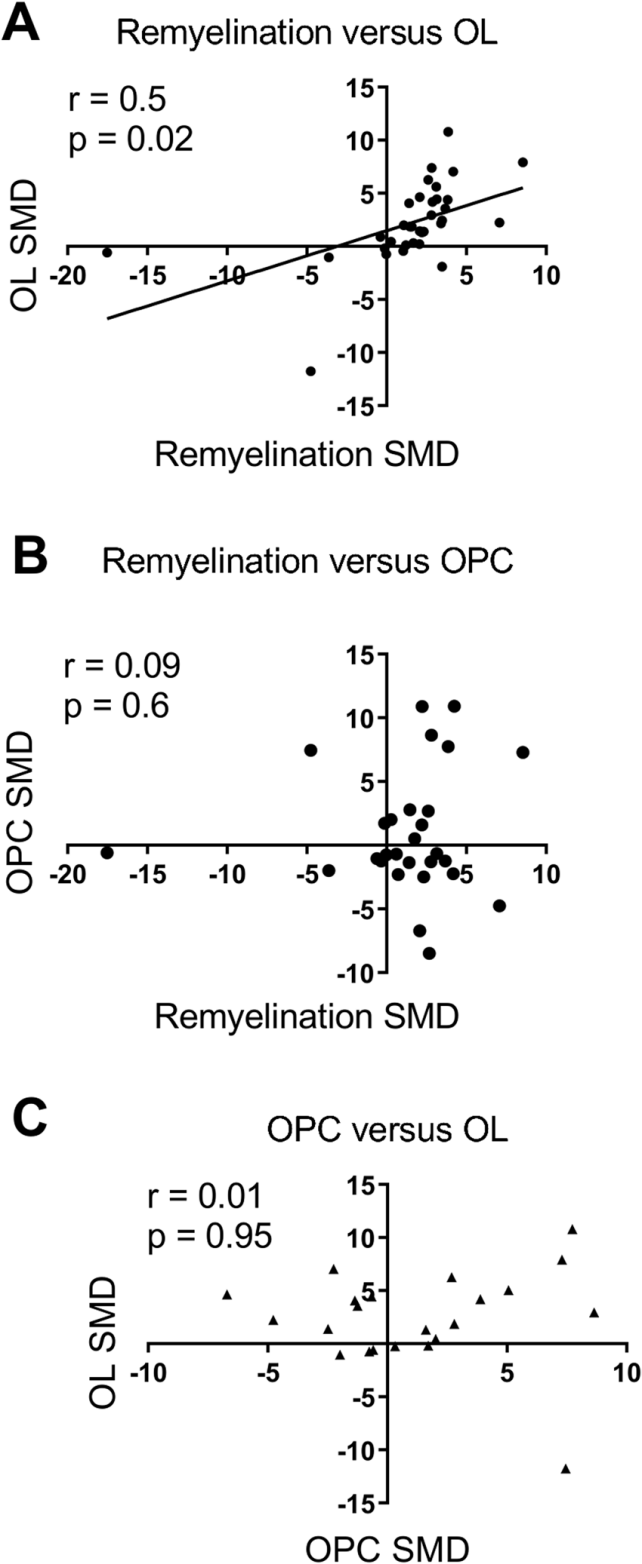
Scatter plot of standardized mean differences (SMD) from the present meta-analysis of therapies which were assessed in at least two outcomes (remyelination, oligodendrocyte cell count (OL), oligodendrocyte precursor cell count (OPC)). Remyelination versus OL (**A**) shows a significant positive correlation (Pearson’s r = 0.51, two-tailed p < 0.02). Neither remyelination versus OPC (**B**) nor OPC versus OL (**C**) show a significant correlation.

Sixteen therapeutic strategies that have not yet been tested in clinical trials seem promising and scored at least one significant increase in one of our 3 remyelination outcomes and no negative effects: Apotransferrin, Benztropine, Epimedium flavonoids (an extract of epimedium sagittatum), Estrogen receptor agonist G1, Iloprost (prostaglandin I2), Indazol chloride (an Estrogen receptor agonist), N6-cyclohexyladenosine (an Adenosine A1 receptor agonist), Neurotrophin 3, Ninjin’yoeito (an extract from different medicinal herbs), Olesoxime (a cholesterol-like compound), Pigment epithelium-derived factor (PEDF), Plateled derived growth factor (PDGF), Sildenafil (a phosphodiesterase inhibitor), siRNA against Nogo-receptor, Sonic hedgehog, and Tocopherol derivate TFA-12 (a Vitamin E derivate). For most therapies, however, only one comparison was available for our meta-analysis. Three of these possible therapies seem very promising as they scored a significant increase in 2 of the 3 outcome measures assessed and no negative effects in the remaining outcome: PDGF, PEDF, and Tocopherol derivate TFA-12 (each with one comparison). In Fig. 10, the meta-analysis outcomes remyelination, oligodendrocyte cell/OPC cell counts are plotted against each other for each therapy, which has been tested in at least two outcomes. The scatter plots demonstrate a good correlation (Pearson’s r = 0.51, two-tailed p = 0.02) between the outcome remyelination and oligodendrocytes.

### Clinical trials

Table 6 summarizes the therapies that were tested in toxic demyelination models that have entered clinical trials (phase 1 to 4). Out of 88 interventions identified by our systematic review, 25 (28%) have already entered clinical trials. Five of these reached FDA-approval (Fingolimod, Fumaric acid ester, Glatiramer acetate, Methotrexate, Methylprednisolone).

**Table 6:** Clinical trials for therapies potentially promoting remyelination with trial information and NCT (if available).

Phase	Count	Trial information	NCT (if available)
1	4		
	Olesoxime	Add-on therapy for Interferon β1	NCT01808885
	Serum-derived human monoclonal IgM22	—	NCT01803867
	Thymosin beta4	—	planned according to RegeneRX
	Triiodothyronine	For RRMS, SPMS, and PPMS	NCT02506751, NCT02760056
**2**	10		
	Anti-Lingo-1 antibody	Add-on therapy for Interferon β1	NCT01864148
	Cyclosporin^61^		
	Electro-acupuncture^62^		
	Electromagnetic field stimulation^63^		
	Erythropoietin	PPMS or SPMS	NCT01144117
	Melatonin	RRMS	NCT01279876
	Quetiapine	For RRMS and SPMS	NCT02087631
	Testosteron	For male patients	NCT00405353
	Vitamin E	Combined with Selenium	NCT00010842
	Ebselen	Combined with Vitamin E	NCT00010842
**3**	3		
	Laquinimod	For RRMS	NCT01047319
	Minocycline	For clinically isolated syndrome	NCT00666887
	Progesterone	To prevent postpartum relapses	NCT00127075
**4**	8		
	Fingolimod	FDA-approved for RRMS	
	Fumaric acid ester	FDA-approved for RRMS	
	Glatiramer acetate	FDA-approved for RRMS	
	Methotrexat	FDA-approved for RRMS	
	Methylprednisolone	FDA-approved for RRMS	
	Omega-3 poly unsaturated fatty acid (PUFA) diet	For RRMS	NCT01842191
	Simvastatin	Add-on therapy for Interferon β1 (and phase 3 for SPMS)	NCT00492765
	Vitamin D	Add-on therapy for Interferon β1	NCT01005095
**Total**	25 (30% of the 84 different therapies from our screening)		

Abbreviations: MS, multiple sclerosis; PP, primary progressive; SP, secondary progressive; RR, relapsing remitting.

## Discussion

Finding remyelinating therapies for MS is an urgent and unmet medical need because no such therapies are currently available. Regeneration of myelin sheaths would re-enable efficient electrical conduction along axons and furthermore protect axons from neurodegeneration^4^. Many such potential approaches have been pre-clinically tested so far. In order to obtain an overview of possible promising remyelinating therapies, we systematically reviewed pre-clinical studies assessing therapies aiming at improving myelin repair. Our review provides evidence that pre-clinically tested therapies significantly increase remyelination and oligodendrocyte cell counts overall. In addition, we show that there are numerous other therapies that seem promising based on our meta-analysis that are not yet tested in clinical trials.

Overall meta-analysis of the outcome remyelination showed that 19 out of 78 therapies (24%) enhanced remyelination. Four therapies dampened the remyelination, among them valproic acid and Simvastatin (1 and 2 comparisons included, respectively). Nevertheless, Simvastatin was, based on experimental studies conducted in EAE, already tested in several clinical trials due to its presumptive immunomodulatory and neuroprotective effects^24^. It appeared to be successful in a recent phase 2 trial in secondary progressive MS patients^25,26^. It failed, however, in a phase 4 trial in relapsing remitting MS^27^. This demonstrates that neuroprotective properties rather than immunomodulation might be its primary mode of action^24^. Besides that, Simvastatin, a blood-brain barrier permeable statin, is an inhibitor of cholesterol synthesis. It has been shown *in vitro* and *in vivo* to dampen the maturation/differentiation of OPCs to myelinating oligodendrocytes via inhibition of the Rho kinase (ROCK) downstream pathway^28,29^. Another statin, Lovastatin has also been shown to affect OPC maturation *in vitro*, namely by inhibition of the cholesterol synthesis thereby affecting the proteolipid protein (PLP) transport towards the myelin sheaths^30^. It seems therefore plausible that one should carefully monitor the effect of statins in the therapy of MS and other demyelinating diseases^29,30^.

Meta-analysis of the outcome oligodendrocyte cell counts demonstrated that 12 out of 43 (28%) therapies increased oligodendrocyte cell counts. Valproic acid was the only intervention significantly decreasing the oligodendrocyte cell count. Together with its increase in the OPC count, oligodendrocyte differentiation blocking properties have been revealed (via inhibition of histone deacetylases)^31^. Intriguingly, this antiepileptic drug has repeatedly shown beneficial effects in EAE and optic neuritis animal models^32,33^. Data from a retrospective cohort study in MS patients were negative, however^34^. This emphasizes that a good pre-clinical scientific fundament for a presumptive remyelinating therapy using also toxic demyelination models can help to decide whether a therapy should be tested in a clinical trial.

Meta-analysis of the outcome OPC count revealed that 7 out of 31 therapies (23%) increased the OPC count whereas 3 decreased it (Apotransferrin, Epimedium flavonoids, Lactacystin). Overall analysis of the outcome OPC count revealed no statistically significant effect. Assessing OPC count as the single outcome for a presumptive remyelinating therapy is not ideal because increased OPC counts can be at the cost of decreased oligodendrocyte counts (cell differentiation block), as shown by the absence of correlation between OPC counts and remyelination in our analysis. This fits also to the extensive evidence showing that OPCs can be present at the border or even inside experimental demyelinated lesions^35,36^ and chronic demyelinated MS lesions^37–42^ without differentiating to oligodendrocytes. Proof of this OPC differentiation block with resulting remyelination failure has led to a paradigm shift in the remyelination field, focusing more on ways to foster OPC differentiation than OPC proliferation^43,44^ (reviewed in^45^).

Interestingly, the meta-analysis outcomes remyelination and oligodendrocyte cell counts showed a significant positive correlation when plotted against each other. This possibly shows that remyelination alone might be a sufficient outcome measure for future pre-clinical trials.

### Clinical relevance and recommendations

Out of 88 included therapies, at least 25 (28%) entered one or more clinical trials and/or reached clinical approval. Our meta-analysis resulted in another 16 therapeutic strategies that have not yet been tested in clinical trials but seem promising (i.e. Apotransferrin, Benztropine, Epimedium flavonoids (extract of epimedium sagittatum), Estrogen receptor agonist G1, Iloprost (prostaglandin I2), Indazol chloride (an Estrogen receptor agonist), N6-cyclohexyladenosine (Adenosine A1 receptor agonist), Neurotrophin 3, Ninjin’yoeito (extract from different medicinal herbs), Olesoxime (cholesterol-like compound), Pigment epithelium-derived factor (PEDF), Plateled derived growth factor (PDGF), Sildenafil (phosphodiesterase inhibitor), siRNA against Nogo-receptor, Sonic hedgehog, and Tocopherol derivate TFA-12 (Vitamin E derivate)). Our results suggest that treatment with PDGF, PEDF, or Tocopherol derivate TFA-12 are particularly interesting for further investigations because treatment with these drugs showed favourable outcomes in two out of three of our remyelinating outcomes.

Following this, it would also be very useful to investigate whether the results of the currently conducted clinical trials show comparable results with our review results.

### Correlation with therapies which have entered clinical trials

For some of the clinical trials the results are already published. Whereas some pre-clinically assessed therapies therapies already reached market approval, the clinical translation is sometimes hampered. A good example are anti-Lingo-1 antibodies. These antibodies effectively and consistently enhanced remyelination in different MS animal models. However, the failure of anti-Lingo-1-antibodies in a phase 2 trial to meet the primary outcome in relapsing/progressive MS patients, a multicomponent analysis evaluating motor and cognitive function as well as disability, were sobering (http://media.biogen.com/press-release/investor-relations/biogen-reports-top-line-results-phase-2-study-opicinumab-anti-lingo). In our meta-analysis, anti-Lingo-1-antibodies did only show a significant increase in remyelination but not in oligodendrocyte cell counts. Cyclosporin is another therapy that showed beneficial effects in the ethidium bromide model^46^ (although not in our meta-analysis) but failed in clinical trials^47^. Of course, some of these therapies made it into clinical trials not because of their results in toxic demyelination models assessed here, but because of their results in neuro-inflammatory animal models such as EAE^48^.

### Limitations

An important limitation of this review, as in many other systematic reviews of animal studies^49^ is that many of the essential methodological details of animal studies included in our review were poorly reported. This seriously hampers reliable risk of bias assessment because in order to assess whether or not results can be influenced by systematic errors and deviates from the truth, all important details regarding a specific type of bias need to be reported. As a consequence, we can not reliably estimate how valid the results of the included studies are. We nevertheless included the poorly reported papers in this review because papers that do not report essential details are not necessarily methodologically impaired^50^. Nevertheless, it is important to emphasize that consistent reporting of essential details regarding experimental design for future animal experiments, as described for example in the ARRIVE guidelines^51^, is urgently needed.

Secondly, in this review we focused on models of toxic demyelination, namely anti-galactocerebroside antibodies, cuprizone, ethidium bromide, and lysolecithin. These models are believed to be more suited to assess potential remyelinating interventions due to a clear temporal separation of de- and remyelination and only minimal overlying inflammatory processes^11^. This is in contrast to viral MS animal models or EAE, the most widely used MS animal model, being yet more suitable to study (neuro-)inflammatory processes of MS^52^. A recent meta-analysis revealed that Cyclosporin, Fingolimod, Glatiramer Acetate, Minocycline, and Vitamin D among others improved neuro-behavioral outcome in EAE^21^. Glatiramer Acetate also had a beneficial effect on remyelination in our meta-analysis. Fingolimod, on the other hand, did not significantly enhance remyelination in our analysis. Both drugs are FDA-approved for relapsing MS. Interestingly, Methylprednisolone showed neither a beneficial effect on neurobehavioral recovery nor on remyelination in the meta-analysis even though being commonly used as treatment for acute MS relapses. This emphasizes that despite the usefulness of MS animal models in developing novel MS therapies, findings cannot be strictly translated to MS.

Thirdly, for many analyses the number of studies was low and the variation between the studies high. This influences the reliability of the conclusions drawn from this systematic review. For example, in this review we pooled the different toxic demyelination models as well as the different methods to assess the outcomes, neglecting strengths and weaknesses of various models and outcomes individually. For example, the gold standard of assessing remyelination is the evidence of thinly myelinated axons in electron microscopy. Thus, the most commonly used readout in the included studies to assess remyelination was the evidence of thinly myelinated axons in electron microscopy (29%). Whereas methods demonstrating thinly myelinated axons are an acceptable compromise to show remyelination (e.g. semithin sections, MBP^53^, or sudan black^54^), other methods are less sensitive and/or specific. LFB was used by 19% of the studies to investigate remyelination upon therapy even though it does not reliably demonstrate single axon/myelin sheath resolution. For now, we have accounted for anticipated heterogeneity by using a random rather than fixed effects meta-analysis.

Furthermore, it is important to realize that no animal model is a perfect match to represent the full clinical situation. The results coming from animal studies are therefore always indirect^55,56^.

When the results of this review will be used to inform the clinical field, the applicability and feasibility of the promising therapeutic strategies mentioned in this paper need to be carefully assessed as well. In addition, potential adverse side effects of therapies in both animals and humans is another important determinant for clinical translation. Safety profile is completely neglected by our analysis (as it is in most pre-clinical studies, however) but needs to be included for clinical translation.

Lastly, we did not perform post-hoc power-calculations due to their limited validity. The sample sizes, however, were small in most of the included studies. Whereas we cannot draw direct conclusions on the power of the here included studies, it has been shown that the average statistical power of studies in neurosciences is very low^57^. With lower power, the likelihood that a statistically significant result reflects a true effect decreases^58^. This has important implications for successful clinical translation of pre-clinical evidence in animals because effects of a potential intervention might be overestimated in underpowered animal studies^57^.

## Conclusions

We identified in this meta-analysis 16 potential therapies to improve remyelination in MS patients that have not yet been tested in clinical trials: Apotransferrin, Benztropine, Epimedium flavonoids, Estrogen receptor agonist G1, Iloprost, Indazol chloride, N6-cyclohexyladenosine, Neurotrophin 3, Ninjin’yoeito, Olesoxime, PEDF, PDGF, Sildenafil, siRNA against Nogo-receptor, Sonic hedgehog, and Tocopherol derivate TFA-12. However, before these therapies should be tested in clinical trials, the safety, applicability, and feasibility of the promising therapies need to be carefully assessed as well. The cross-species translation to humans in clinical trials so far was, however, less successful in some cases. Improving the reporting and possibly the methodological quality of animal studies can help to smoothen this important translation of remyelinating therapies to clinical use. This can also help to reduce animal numbers by making animal research more reliable.

## Materials and Methods

This systematic review investigates the effects of remyelination promoting therapies in multiple sclerosis animal models. The inclusion criteria and method of analysis were specified in advance and documented in a protocol (Ineichen *et al*. (2017) and put online on the SYRCLE website in the section protocols (www.syrcle.nl).

### Search strategy and paper selection

A comprehensive search string to identify studies assessing potentially remyelinating interventions in an animal model of toxic demyelination (Anti-galactocerebroside antibodies, ethidium bromide, cuprizone, and/or lysolecithin) was generated. The following data bases were searched for matches: EMBASE, go3R, Medline, Pubmed, Scopus, and Web of Sciences (last search 04^th^ of July, 2016). See supplementary file for exact search strings (Supplementary Information). All animal species, publication dates, and languages were included to the data base search.

Studies were included in this systematic review when they met the following inclusion criteria: (1) the study was an original peer reviewed full paper that published unique data, (2) the study included an appropriate control group (vehicle only treatment), (3) an animal model of toxic demyelination (Anti-galactocerebroside antibodies, ethidium bromide, cuprizone, and/or lysolecithin) was used, (4) An outcome measure related to remyelination or (pre-)myelinating cell count was used.

Papers were screened for relevance by two independent reviewers (AG and BVI for anti-galactocerebroside antibodies, ethidium bromide, and cuprizone; GG and LB for lysolecithin). Reviews were excluded but retained as a source for potential studies and for discussion.

### Data extraction

The following study characteristics were extracted from the full-texts: type of demyelination model, tested intervention, application regimen, species, strain, sex, and number of animals per group. As study outcomes measures, we extracted the mean and variance (Standard deviation (SD) or standard error of the mean (SEM)) of the outcome most closely related to remyelination. Since certain remyelination outcomes more reliably measure remyelination than others (e.g electron microscopy is more reliable than quantifying remyelination by measuring optical density of an LFB stained lesion), the following extraction priority was used for the remyelination outcomes: (1) Electron microscopy analysis of remyelination, (2) Toluidine blue/semithin section analysis of remyelination, (3) Analysis of other stainings that allow measurement of remyelination (i.e. disproportionally thinly myelinated myelin sheets surrounding axons, e.g. MBP staining^53^, sudan black staining^54^), (4) Analysis of lesion volume/area in other stainings (e.g. MBP or LFB), (5) Analysis of lesion volume/area in MRI, and (6) Analysis of optical intensity in other stainings (e.g. MBP or LFB). Additionally, mean counts and variance (SD or SEM) of OPCs and/or oligodendrocytes were extracted if available. AG and BVI extracted the data for anti-galactocerebroside-antibodies, ethidium bromide, and cuprizone models; GG and LB extracted the data for all lysolecithin models). BVI checked if all data were extracted correctly. Disagreement between two extractors was solved by checking the data in the publications together. The interrater agreement was 60% for remyelination and oligodendrocytes, and 84% for OPCs.

In first priority, data was extracted from text or tables, in second priority from graphs using universal desktop ruler software (AVP Software Development, USA). In case of missing data, corresponding authors were contacted with maximally one email.

When the group size was reported as a range (e.g., 6–9), the mean number of animals was used in our analysis.

### Quality assessment

To estimate risk of bias (RoB) of each study, we judged the study using the SYRCLE RoB-assessment tool, which is an adapted version of the Cochrane RoB tool^20^. Two independent reviewers assessed the risk of bias in each included paper (AG and BVI for anti-galactocerebroside antibodies, ethidium bromide, and cuprizone; GG and LB for lysolecithin). A ‘yes’ score indicates low risk of bias; a ‘no’ score indicates high risk of bias; and a ‘?’ score indicates unknown risk of bias.

To overcome the problem of judging too many items as “unclear risk of bias” because reporting of experimental details on animals, methods and materials is very poor, we added two items on reporting: reporting of any measure of randomization, reporting of any measure of blinding.

Furthermore, we looked at 3 more items to measure overall quality of the included papers according to the consensus statement *good laboratory practice* in the modelling of stroke^59^: sample size calculations provided, reporting of animal welfare, and statement of a potential conflict of interest. For these three items, a ‘yes’ score indicates ‘reported’, and a ‘no’ score indicates ‘unreported’.

### Meta-analysis

Data were analyzed using Comprehensive Meta-Analysis (CMA version2.0). Because in this Meta-analysis different studies used different scales to measure the same outcome, we calculated the hedges g standardized mean difference (SMD) instead of the raw mean difference (the mean of the experimental group minus the mean of the control group divided by the pooled SDs of the two groups).

When a control group served more than one experimental group, the number of observations in that control group was, for the purpose of the meta-analysis, divided by the number of experimental groups served, to make sure that the experiment does not receive too much weight in the meta analyses.

Individual effect sizes (in this case SMDs) were subsequently pooled to obtain an overall SMD and 95% confidence interval. We used the random effects model [14], which takes into account the precision of individual studies and the variation between studies and weighs each study accordingly.

Sources for heterogeneity were explored with predefined subgroup analyses for intervention type, timing of the intervention (prophylactic or therapeutic) and type of MS model used (cuprizone, lysolecithin, ethidium bromide and anti-galactocerebroside antibodies).

We expected the variance to be comparable within the subgroups; therefore, we assumed a common among-study variance across subgroups. Statistical differences between subgroups were only assessed when subgroups contained at least 5 independent experiments per subgroup.

For those subgroup analyses, we adjusted our significance level according to the conservative Bonferroni method to account for multiple analyses (p* number of comparisons). However, differences between subgroups should be interpreted with caution and should only be used for constructing new hypotheses rather than for drawing final conclusions. No sub-subgroup analyses were calculated due to low number of experiments per therapeutic approach.

We used funnel plots, Egger regression and Trim and Fill analysis to search for evidence of publication bias. Because SMDs may cause funnelplot distortion we plotted the SMD against a sample size-based precision estimate (1/√(n))^60^.

## Electronic supplementary material

Supplementary Dataset 1
