# Supplementary material for: Remyelination promoting therapies in multiple sclerosis animal models: a systematic review and meta-analysis
Source: Sci Rep. 2019 Jan 29;9:822. doi: 10.1038/s41598-018-35734-4 (PMC6351564; doi:10.1038/s41598-018-35734-4)
Supplement: Supplementary file 1 — Supplementary Dataset 1 [file 41598_2018_35734_MOESM1_ESM.pdf]

Remyelination promoting therapies in multiple sclerosis animal models: a systematic review and meta-analysis

Carlijn R. Hooijmans[2]*, Martin Hlavica[1]*, Florian A. F. Schuler[3%], Nicolas Good[1], Andrin Good[1], Lisa Baumgartner[1], Gianluca Galeno[1], Marc P. Schneider[1], Tarzis Jung[4], Rob de Vries[2], Benjamin V. Ineichen[1&#]

[1] Brain Research Institute, University of Zurich and Department of Health Sciences and Technology, ETH Zurich, 8057 Zurich, Switzerland

[2]Systematic Review Centre for Laboratory Animal Experimentation (SYRCLE), Department of Health Evidence, Radboud University Medical Center, Nijmegen, The Netherlands

[3]Institute of Neuroinformatics, University of Zurich and ETH Zurich, Switzerland

[4]Waidspital Zurich, Department of Radiology, Tiechestrasse 99, 8037 Zurich, Switzerland

[&]Current address: Waidspital Zurich, Department of Radiology, Tiechestrasse 99, 8037 Zurich, Switzerland

[%]Current address: Department of Neurology, Inselspital, Bern University Hospital, University of Bern, Switzerland

*CRH and MH contributed equally and share the first authorship.

Supplementary information

Search strings

EMBASE

<u>Cuprizone</u>

('cuprizone'/exp OR (Cuprizone OR Cuprizon OR Cuprizane OR Cupferazone OR biscycloxaldihydrazone):ab,ti OR (bicyclohexanone NEAR/3 oxalyldihydrazone):ab,ti) AND ('myelin'/exp OR 'demyelinating disease'/exp OR 'remyelinization'/exp OR 'oligodendroglia'/exp OR 'central nervous system'/exp OR (myelin OR (myelinic AND body) OR remyelination OR demyelination OR oligodendrocyte* OR oligodendroglia OR CNS OR central nervous system):ab,ti OR (cerebrospinal NEAR/3 axi*):ab,ti OR (spinal* NEAR/3 (cord* OR medulla OR marrow)):ab,ti OR spinalcord*:ab,ti) AND ('animal experiment'/exp OR 'animal model'/exp OR 'experimental animal'/exp OR 'transgenic animal'/exp OR 'male animal'/exp OR 'female animal'/exp OR 'juvenile animal'/exp OR animal/de OR chordata/de OR vertebrate/de OR tetrapod/de OR fish/exp OR amniote/de OR amphibia/exp OR mammal/de OR reptile/exp OR sauropsid/exp OR therian/de OR monotremate/exp OR 'placental mammals'/de OR marsupial/exp OR Euarchontoglires/de OR Afrotheria/exp OR Boreoeutheria/exp OR Laurasiatheria/exp OR Xenarthra/exp OR primate/de OR Dermoptera/exp OR Glires/exp OR Scandentia/exp OR Haplorhini/de OR prosimian/exp OR simian/de OR tarsiiform/exp OR Catarrhini/de OR Platyrrhini/exp OR ape/de OR Cercopithecidae/exp OR hominid/de OR hylobatidae/exp OR chimpanzee/exp OR gorilla/exp OR 'orang utan'/exp OR (animal OR animals OR pisces OR fish OR fishes OR catfish OR catfishes OR sheatfish OR silurus OR arius OR heteropneustes OR clarias OR gariepinus OR "fathead minnow" OR "fathead minnows" OR pimephales OR promelas OR cichlidae OR trout OR trouts OR char OR chars OR salvelinus OR salmo OR oncorhynchus OR guppy OR guppies OR millionfish OR poecilia OR goldfish OR goldfishes OR carassius OR auratus OR mullet OR mullets OR mugil OR curema OR shark OR sharks OR cod OR cods OR gadus OR morhua OR carp OR carps OR cyprinus OR carpio OR killifish OR eel OR eels OR anguilla OR zander OR sander OR lucioperca OR stizostedion OR turbot OR turbots OR psetta OR flatfish OR flatfishes OR plaice OR pleuronectes OR platessa OR tilapia OR tilapias OR oreochromis OR sarotherodon OR "common sole" OR "dover sole" OR solea OR zebrafish OR zebrafishes OR danio OR rerio OR seabass OR dicentrarchus OR labrax OR morone OR lamprey OR lampreys OR petromyzon OR pumpkinseed OR pumpkinseeds OR lepomis OR gibbosus OR herring OR clupea OR harengus OR amphibia OR amphibian OR amphibians OR anura OR salientia OR frog OR frogs OR rana OR toad OR toads OR bufo OR xenopus OR laevis OR bombina OR epidalea OR calamita OR salamander OR salamanders OR newt OR newts OR triturus OR reptilia OR reptile OR reptiles OR "bearded dragon" OR pogona OR vitticeps OR iguana OR iguanas OR lizard OR lizards OR "anguis fragilis" OR turtle OR turtles OR snakes OR snake OR aves OR bird OR birds OR quail OR quails OR coturnix OR bobwhite OR colinus OR virginianus OR poultry OR poultries OR fowl OR fowls OR chicken OR chickens OR gallus OR "zebra finch" OR taeniopygia OR guttata OR canary OR canaries OR serinus OR canaria OR parakeet OR parakeets OR grasskeet OR parrot OR parrots OR psittacine OR psittacines OR shelduck OR tadorna OR goose OR geese OR branta OR leucopsis OR woodlark OR lullula OR flycatcher OR ficedula OR hypoleuca OR dove OR doves OR geopelia OR cuneata OR duck OR ducks OR greylag OR graylag OR anser OR harrier OR "circus pygargus" OR "red knot" OR "great knot" OR calidris OR canutus OR godwit OR limosa OR lapponica OR meleagris OR gallopavo OR jackdaw OR corvus OR monedula OR ruff OR philomachus OR pugnax OR lapwing OR peewit OR plover OR vanellus OR swan OR cygnus OR columbianus OR bewickii OR gull OR chroicocephalus OR ridibundus OR albifrons OR "great tit" OR parus OR aythya OR fuligula OR streptopelia OR risoria OR spoonbill OR platalea OR leucorodia OR blackbird OR turdus OR merula OR "blue tit" OR cyanistes OR pigeon OR pigeons OR columba OR

pintail OR anas OR starling OR sturnus OR owl OR "athene noctua" OR pochard OR ferina OR cockatiel OR nymphicus OR hollandicus OR skylark OR alauda OR tern OR sterna OR teal OR crecca OR oystercatcher OR haematopus OR ostralegus OR shrew OR shrews OR sorex OR araneus OR crocidura OR russula OR "european mole" OR talpa OR chiroptera OR bat OR bats OR eptesicus OR serotinus OR myotis OR dasycneme OR daubentonii OR pipistrelle OR pipistrellus OR cat OR cats OR felis OR catus OR feline OR dog OR dogs OR canis OR canine OR canines OR otter OR otters OR lutra OR badger OR badgers OR meles OR fitchew OR fitch OR foumart or foulmart OR ferrets OR ferret OR polecat OR polecats OR mustela OR putorius OR weasel OR weasels OR fox OR foxes OR vulpes OR "common seal" OR phoca OR vitulina OR "grey seal" OR halichoerus OR horse OR horses OR equus OR equine OR equidae OR donkey OR donkeys OR mule OR mules OR pig OR pigs OR swine OR swines OR hog OR hogs OR boar OR boars OR porcine OR piglet OR piglets OR sus OR scrofa OR llama OR llamas OR lama OR glama OR deer OR deers OR cervus OR elaphus OR cow OR cows OR "bos taurus" OR "bos indicus" OR bovine OR bull OR bulls OR cattle OR bison OR bisons OR sheep OR sheeps OR "ovis aries" OR ovine OR lamb OR lambs OR mouflon OR mouflons OR goat OR goats OR capra OR caprine OR chamois OR rupicapra OR leporidae OR lagomorpha OR lagomorph OR rabbit OR rabbits OR oryctolagus OR cuniculus OR laprine OR hares OR lepus OR rodentia OR rodent OR rodents OR murinae OR mouse OR mice OR mus OR musculus OR murine OR woodmouse OR apodemus OR rat OR rats OR rattus OR norvegicus OR "guinea pig" OR "guinea pigs" OR cavia OR porcellus OR hamster OR hamsters OR mesocricetus OR cricetulus OR cricetus OR gerbil OR gerbils OR jird OR jirds OR meriones OR unguiculatus OR jerboa OR jerboas OR jaculus OR chinchilla OR chinchillas OR beaver OR beavers OR "castor fiber" OR "castor canadensis" OR sciuridae OR squirrel OR squirrels OR sciurus OR chipmunk OR chipmunks OR marmot OR marmots OR marmota OR suslik OR susliks OR spermophilus OR cynomys OR cottonrat OR cottonrats OR sigmodon OR vole OR voles OR microtus OR myodes OR glareolus OR primate OR primates OR prosimian OR prosimians OR lemur OR lemurs OR lemuridae OR loris OR "bush baby" OR "bush babies" OR bushbaby OR bushbabies OR galago OR galagos OR anthropoidea OR anthropoids OR simian OR simians OR monkey OR monkeys OR marmoset OR marmosets OR callithrix OR cebuella OR tamarin OR tamarins OR saguinus OR leontopithecus OR "squirrel monkey" OR "squirrel monkeys" OR saimiri OR "night monkey" OR "night monkeys" OR "owl monkey" OR "owl monkeys" OR douroucoulis OR aotus OR "spider monkey" OR "spider monkeys" OR ateles OR baboon OR baboons OR papio OR "rhesus monkey" OR macaque OR macaca OR mulatta OR cynomolgus OR fascicularis OR "green monkey" OR "green monkeys" OR chlorocebus OR vervet OR vervets OR pygerythrus OR hominoidea OR ape OR apes OR hylobatidae OR gibbon OR gibbons OR siamang OR siamangs OR nomascus OR symphalangus OR hominidae OR orangutan OR orangutans OR pongo OR chimpanzee OR chimpanzees OR "pan troglodytes" OR bonobo OR bonobos OR "pan paniscus" OR gorilla OR gorillas OR troglodytes):ab,ti)

<u>Ethidium bromide</u>

('ethidium bromide'/exp OR etbr:ab,ti OR homidium:ab,ti OR ((ethidium OR homidium OR novidium) NEAR/3 (hydrobromide OR bromide)):ab,ti) AND ('myelin'/exp OR 'demyelinating disease'/exp OR 'remyelinization'/exp OR 'oligodendroglia'/exp OR 'central nervous system'/exp OR (myelin OR (myelinic AND body) OR remyelination OR demyelination OR oligodendrocyte* OR oligodendroglia OR CNS OR central nervous system):ab,ti OR (cerebrospinal NEAR/3 axi*):ab,ti OR (spinal* NEAR/3 (cord* OR medulla OR marrow)):ab,ti OR spinalcord*:ab,ti) AND ('animal experiment'/exp OR 'animal model'/exp OR 'experimental animal'/exp OR 'transgenic animal'/exp OR 'male animal'/exp OR 'female animal'/exp OR 'juvenile animal'/exp OR animal/de OR chordata/de OR vertebrate/de OR tetrapod/de OR fish/exp OR amniote/de OR amphibia/exp OR mammal/de OR reptile/exp OR sauropsid/exp OR therian/de OR monotremate/exp OR 'placental mammals'/de OR marsupial/exp OR Euarchontoglires/de OR Afrotheria/exp OR Boreoeutheria/exp OR Laurasiatheria/exp OR

Xenarthra/exp OR primate/de OR Dermoptera/exp OR Glires/exp OR Scandentia/exp OR Haplorhini/de OR prosimian/exp OR simian/de OR tarsiiform/exp OR Catarrhini/de OR Platyrrhini/exp OR ape/de OR Cercopithecidae/exp OR hominid/de OR hylobatidae/exp OR chimpanzee/exp OR gorilla/exp OR 'orang utan'/exp OR (animal OR animals OR pisces OR fish OR fishes OR catfish OR catfishes OR sheatfish OR silurus OR arius OR heteropneustes OR clarias OR gariepinus OR "fathead minnow" OR "fathead minnows" OR pimephales OR promelas OR cichlidae OR trout OR trouts OR char OR chars OR salvelinus OR salmo OR oncorhynchus OR guppy OR guppies OR millionfish OR poecilia OR goldfish OR goldfishes OR carassius OR auratus OR mullet OR mullets OR mugil OR curema OR shark OR sharks OR cod OR cods OR gadus OR morhua OR carp OR carps OR cyprinus OR carpio OR killifish OR eel OR eels OR anguilla OR zander OR sander OR lucioperca OR stizostedion OR turbot OR turbots OR psetta OR flatfish OR flatfishes OR plaice OR pleuronectes OR platessa OR tilapia OR tilapias OR oreochromis OR sarotherodon OR "common sole" OR "dover sole" OR solea OR zebrafish OR zebrafishes OR danio OR rerio OR seabass OR dicentrarchus OR labrax OR morone OR lamprey OR lampreys OR petromyzon OR pumpkinseed OR pumpkinseeds OR lepomis OR gibbosus OR herring OR clupea OR harengus OR amphibia OR amphibian OR amphibians OR anura OR salientia OR frog OR frogs OR rana OR toad OR toads OR bufo OR xenopus OR laevis OR bombina OR epidalea OR calamita OR salamander OR salamanders OR newt OR newts OR triturus OR reptilia OR reptile OR reptiles OR "bearded dragon" OR pogona OR vitticeps OR iguana OR iguanas OR lizard OR lizards OR "anguis fragilis" OR turtle OR turtles OR snakes OR snake OR aves OR bird OR birds OR quail OR quails OR coturnix OR bobwhite OR colinus OR virginianus OR poultry OR poultries OR fowl OR fowls OR chicken OR chickens OR gallus OR "zebra finch" OR taeniopygia OR guttata OR canary OR canaries OR serinus OR canaria OR parakeet OR parakeets OR grasskeet OR parrot OR parrots OR psittacine OR psittacines OR shelduck OR tadorna OR goose OR geese OR branta OR leucopsis OR woodlark OR lullula OR flycatcher OR ficedula OR hypoleuca OR dove OR doves OR geopelia OR cuneata OR duck OR ducks OR greylag OR graylag OR anser OR harrier OR "circus pygargus" OR "red knot" OR "great knot" OR calidris OR canutus OR godwit OR limosa OR lapponica OR meleagris OR gallopavo OR jackdaw OR corvus OR monedula OR ruff OR philomachus OR pugnax OR lapwing OR peewit OR plover OR vanellus OR swan OR cygnus OR columbianus OR bewickii OR gull OR chroicocephalus OR ridibundus OR albifrons OR "great tit" OR parus OR aythya OR fuligula OR streptopelia OR risoria OR spoonbill OR platalea OR leucorodia OR blackbird OR turdus OR merula OR "blue tit" OR cyanistes OR pigeon OR pigeons OR columba OR pintail OR anas OR starling OR sturnus OR owl OR "athene noctua" OR pochard OR ferina OR cockatiel OR nymphicus OR hollandicus OR skylark OR alauda OR tern OR sterna OR teal OR crecca OR oystercatcher OR haematopus OR ostralegus OR shrew OR shrews OR sorex OR araneus OR crocidura OR russula OR "european mole" OR talpa OR chiroptera OR bat OR bats OR eptesicus OR serotinus OR myotis OR dasycneme OR daubentonii OR pipistrelle OR pipistrellus OR cat OR cats OR felis OR catus OR feline OR dog OR dogs OR canis OR canine OR canines OR otter OR otters OR lutra OR badger OR badgers OR meles OR fitchew OR fitch OR foumart or foulmart OR ferrets OR ferret OR polecat OR polecats OR mustela OR putorius OR weasel OR weasels OR fox OR foxes OR vulpes OR "common seal" OR phoca OR vitulina OR "grey seal" OR halichoerus OR horse OR horses OR equus OR equine OR equidae OR donkey OR donkeys OR mule OR mules OR pig OR pigs OR swine OR swines OR hog OR hogs OR boar OR boars OR porcine OR piglet OR piglets OR sus OR scrofa OR llama OR llamas OR lama OR glama OR deer OR deers OR cervus OR elaphus OR cow OR cows OR "bos taurus" OR "bos indicus" OR bovine OR bull OR bulls OR cattle OR bison OR bisons OR sheep OR sheeps OR "ovis aries" OR ovine OR lamb OR lambs OR mouflon OR mouflons OR goat OR goats OR capra OR caprine OR chamois OR rupicapra OR leporidae OR lagomorpha OR lagomorph OR rabbit OR rabbits OR oryctolagus OR cuniculus OR laprine OR hares OR lepus OR rodentia OR rodent OR rodents OR murinae OR mouse OR mice OR mus OR musculus OR murine OR woodmouse OR apodemus OR rat OR rats OR rattus OR norvegicus OR "guinea pig" OR "guinea pigs" OR cavia OR porcellus OR hamster OR hamsters OR mesocricetus

OR cricetulus OR cricetus OR gerbil OR gerbils OR jird OR jirds OR meriones OR unguiculatus OR jerboa OR jerboas OR jaculus OR chinchilla OR chinchillas OR beaver OR beavers OR "castor fiber" OR "castor canadensis" OR sciuridae OR squirrel OR squirrels OR sciurus OR chipmunk OR chipmunks OR marmot OR marmots OR marmota OR suslik OR susliks OR spermophilus OR cynomys OR cottonrat OR cottonrats OR sigmodon OR vole OR voles OR microtus OR myodes OR glareolus OR primate OR primates OR prosimian OR prosimians OR lemur OR lemurs OR lemuridae OR loris OR "bush baby" OR "bush babies" OR bushbaby OR bushbabies OR galago OR galagos OR anthropoidea OR anthropoids OR simian OR simians OR monkey OR monkeys OR marmoset OR marmosets OR callithrix OR cebuella OR tamarin OR tamarins OR saguinus OR leontopithecus OR "squirrel monkey" OR "squirrel monkeys" OR saimiri OR "night monkey" OR "night monkeys" OR "owl monkey" OR "owl monkeys" OR douroucoulis OR aotus OR "spider monkey" OR "spider monkeys" OR ateles OR baboon OR baboons OR papio OR "rhesus monkey" OR macaque OR macaca OR mulatta OR cynomolgus OR fascicularis OR "green monkey" OR "green monkeys" OR chlorocebus OR vervet OR vervets OR pygerythrus OR hominoidea OR ape OR apes OR hylobatidae OR gibbon OR gibbons OR siamang OR siamangs OR nomascus OR symphalangus OR hominidae OR orangutan OR orangutans OR pongo OR chimpanzee OR chimpanzees OR "pan troglodytes" OR bonobo OR bonobos OR "pan paniscus" OR gorilla OR gorillas OR troglodytes):ab,ti)

<u>Lysolecithin</u>

('lysophosphatidylcholine'/exp OR lysophosphatidylcholine*:ab,ti OR lysolecithin*:ab,ti OR (choline NEAR/3 lysophosphatid*):ab,ti OR (phosphatidylcholine NEAR/3 lyso):ab,ti) AND ('myelin'/exp OR 'demyelinating disease'/exp OR 'remyelinization'/exp OR 'oligodendroglia'/exp OR 'central nervous system'/exp OR (myelin OR (myelinic AND body) OR remyelination OR demyelination OR oligodendrocyte* OR oligodendroglia OR CNS OR central nervous system):ab,ti OR (cerebrospinal NEAR/3 axi*):ab,ti OR (spinal* NEAR/3 (cord* OR medulla OR marrow)):ab,ti OR spinalcord*:ab,ti) AND ('animal experiment'/exp OR 'animal model'/exp OR 'experimental animal'/exp OR 'transgenic animal'/exp OR 'male animal'/exp OR 'female animal'/exp OR 'juvenile animal'/exp OR animal/de OR chordata/de OR vertebrate/de OR tetrapod/de OR fish/exp OR amniote/de OR amphibia/exp OR mammal/de OR reptile/exp OR sauropsid/exp OR therian/de OR monotremate/exp OR 'placental mammals'/de OR marsupial/exp OR Euarchontoglires/de OR Afrotheria/exp OR Boreoeutheria/exp OR Laurasiatheria/exp OR Xenarthra/exp OR primate/de OR Dermoptera/exp OR Glires/exp OR Scandentia/exp OR Haplorhini/de OR prosimian/exp OR simian/de OR tarsiiform/exp OR Catarrhini/de OR Platyrrhini/exp OR ape/de OR Cercopithecidae/exp OR hominid/de OR hylobatidae/exp OR chimpanzee/exp OR gorilla/exp OR 'orang utan'/exp OR (animal OR animals OR pisces OR fish OR fishes OR catfish OR catfishes OR sheatfish OR silurus OR arius OR heteropneustes OR clarias OR gariepinus OR "fathead minnow" OR "fathead minnows" OR pimephales OR promelas OR cichlidae OR trout OR trouts OR char OR chars OR salvelinus OR salmo OR oncorhynchus OR guppy OR guppies OR millionfish OR poecilia OR goldfish OR goldfishes OR carassius OR auratus OR mullet OR mullets OR mugil OR curema OR shark OR sharks OR cod OR cods OR gadus OR morhua OR carp OR carps OR cyprinus OR carpio OR killifish OR eel OR eels OR anguilla OR zander OR sander OR lucioperca OR stizostedion OR turbot OR turbots OR psetta OR flatfish OR flatfishes OR plaice OR pleuronectes OR platessa OR tilapia OR tilapias OR oreochromis OR sarotherodon OR "common sole" OR "dover sole" OR solea OR zebrafish OR zebrafishes OR danio OR rerio OR seabass OR dicentrarchus OR labrax OR morone OR lamprey OR lampreys OR petromyzon OR pumpkinseed OR pumpkinseeds OR lepomis OR gibbosus OR herring OR clupea OR harengus OR amphibia OR amphibian OR amphibians OR anura OR salientia OR frog OR frogs OR rana OR toad OR toads OR bufo OR xenopus OR laevis OR bombina OR epidalea OR calamita OR salamander OR salamanders OR newt

OR newts OR triturus OR reptilia OR reptile OR reptiles OR "bearded dragon" OR pogona OR vitticeps OR iguana OR iguanas OR lizard OR lizards OR "anguis fragilis" OR turtle OR turtles OR snakes OR snake OR aves OR bird OR birds OR quail OR quails OR coturnix OR bobwhite OR colinus OR virginianus OR poultry OR poultries OR fowl OR fowls OR chicken OR chickens OR gallus OR "zebra finch" OR taeniopygia OR guttata OR canary OR canaries OR serinus OR canaria OR parakeet OR parakeets OR grasskeet OR parrot OR parrots OR psittacine OR psittacines OR shelduck OR tadorna OR goose OR geese OR branta OR leucopsis OR woodlark OR lullula OR flycatcher OR ficedula OR hypoleuca OR dove OR doves OR geopelia OR cuneata OR duck OR ducks OR greylag OR graylag OR anser OR harrier OR "circus pygargus" OR "red knot" OR "great knot" OR calidris OR canutus OR godwit OR limosa OR lapponica OR meleagris OR gallopavo OR jackdaw OR corvus OR monedula OR ruff OR philomachus OR pugnax OR lapwing OR peewit OR plover OR vanellus OR swan OR cygnus OR columbianus OR bewickii OR gull OR chroicocephalus OR ridibundus OR albifrons OR "great tit" OR parus OR aythya OR fuligula OR streptopelia OR risoria OR spoonbill OR platalea OR leucorodia OR blackbird OR turdus OR merula OR "blue tit" OR cyanistes OR pigeon OR pigeons OR columba OR pintail OR anas OR starling OR sturnus OR owl OR "athene noctua" OR pochard OR ferina OR cockatiel OR nymphicus OR hollandicus OR skylark OR alauda OR tern OR sterna OR teal OR crecca OR oystercatcher OR haematopus OR ostralegus OR shrew OR shrews OR sorex OR araneus OR crocidura OR russula OR "european mole" OR talpa OR chiroptera OR bat OR bats OR eptesicus OR serotinus OR myotis OR dasycneme OR daubentonii OR pipistrelle OR pipistrellus OR cat OR cats OR felis OR catus OR feline OR dog OR dogs OR canis OR canine OR canines OR otter OR otters OR lutra OR badger OR badgers OR meles OR fitchew OR fitch OR foumart or foulmart OR ferrets OR ferret OR polecat OR polecats OR mustela OR putorius OR weasel OR weasels OR fox OR foxes OR vulpes OR "common seal" OR phoca OR vitulina OR "grey seal" OR halichoerus OR horse OR horses OR equus OR equine OR equidae OR donkey OR donkeys OR mule OR mules OR pig OR pigs OR swine OR swines OR hog OR hogs OR boar OR boars OR porcine OR piglet OR piglets OR sus OR scrofa OR llama OR llamas OR lama OR glama OR deer OR deers OR cervus OR elaphus OR cow OR cows OR "bos taurus" OR "bos indicus" OR bovine OR bull OR bulls OR cattle OR bison OR bisons OR sheep OR sheeps OR "ovis aries" OR ovine OR lamb OR lambs OR mouflon OR mouflons OR goat OR goats OR capra OR caprine OR chamois OR rupicapra OR leporidae OR lagomorpha OR lagomorph OR rabbit OR rabbits OR oryctolagus OR cuniculus OR laprine OR hares OR lepus OR rodentia OR rodent OR rodents OR murinae OR mouse OR mice OR mus OR musculus OR murine OR woodmouse OR apodemus OR rat OR rats OR rattus OR norvegicus OR "guinea pig" OR "guinea pigs" OR cavia OR porcellus OR hamster OR hamsters OR mesocricetus OR cricetulus OR cricetus OR gerbil OR gerbils OR jird OR jirds OR meriones OR unguiculatus OR jerboa OR jerboas OR jaculus OR chinchilla OR chinchillas OR beaver OR beavers OR "castor fiber" OR "castor canadensis" OR sciuridae OR squirrel OR squirrels OR sciurus OR chipmunk OR chipmunks OR marmot OR marmots OR marmota OR suslik OR susliks OR spermophilus OR cynomys OR cottonrat OR cottonrats OR sigmodon OR vole OR voles OR microtus OR myodes OR glareolus OR primate OR primates OR prosimian OR prosimians OR lemur OR lemurs OR lemuridae OR loris OR "bush baby" OR "bush babies" OR bushbaby OR bushbabies OR galago OR galagos OR anthropoidea OR anthropoids OR simian OR simians OR monkey OR monkeys OR marmoset OR marmosets OR callithrix OR cebuella OR tamarin OR tamarins OR saguinus OR leontopithecus OR "squirrel monkey" OR "squirrel monkeys" OR saimiri OR "night monkey" OR "night monkeys" OR "owl monkey" OR "owl monkeys" OR douroucoulis OR aotus OR "spider monkey" OR "spider monkeys" OR ateles OR baboon OR baboons OR papio OR "rhesus monkey" OR macaque OR macaca OR mulatta OR cynomolgus OR fascicularis OR "green monkey" OR "green monkeys" OR chlorocebus OR vervet OR vervets OR pygerythrus OR hominoidea OR ape OR apes OR hylobatidae OR gibbon OR gibbons OR siamang OR siamangs OR nomascus OR symphalangus OR hominidae OR orangutan OR orangutans OR pongo OR chimpanzee OR chimpanzees OR "pan troglodytes" OR bonobo OR bonobos OR "pan paniscus" OR gorilla OR gorillas OR troglodytes):ab,ti)

Anti-galactocerebroside antibodies/complement

("galactocerebroside antibody":de OR (anti-galactocerebroside* OR antigalactocerebroside* OR "anti galactocerebroside" OR "galactocerebroside antibody" OR "galactocerebroside antibodies" OR anti-GalC OR "anti-GalC" OR "GalC antibody" OR "GalC antibodies" OR anti-galactosylceramide* OR "anti galactosylceramide" OR "anti galactosylceramide*"):ti,ab OR (anti* NEAR/6 ("acylsphingosine galactoside" OR "ceramide galactoside" OR cerebrogalactoside* OR galactocerebroside* OR "galactosyl acylsphingosine" OR "galactosyl ceramide" OR galactosylceramide*)):ti,ab) AND ('myelin'/exp OR 'demyelinating disease'/exp OR 'remyelinization'/exp OR 'oligodendroglia'/exp OR 'central nervous system'/exp OR (myelin OR (myelinic AND body) OR remyelination OR demyelination OR oligodendrocyte* OR oligodendroglia OR CNS OR central nervous system):ab,ti OR (cerebrospinal NEAR/3 axi*):ab,ti OR (spinal* NEAR/3 (cord* OR medulla OR marrow)):ab,ti OR spinalcord*:ab,ti) AND ('animal experiment'/exp OR 'animal model'/exp OR 'experimental animal'/exp OR 'transgenic animal'/exp OR 'male animal'/exp OR 'female animal'/exp OR 'juvenile animal'/exp OR animal/de OR chordata/de OR vertebrate/de OR tetrapod/de OR fish/exp OR amniote/de OR amphibia/exp OR mammal/de OR reptile/exp OR sauropsid/exp OR therian/de OR monotremate/exp OR 'placental mammals'/de OR marsupial/exp OR Euarchontoglires/de OR Afrotheria/exp OR Boreoeutheria/exp OR Laurasiatheria/exp OR Xenarthra/exp OR primate/de OR Dermoptera/exp OR Glires/exp OR Scandentia/exp OR Haplorhini/de OR prosimian/exp OR simian/de OR tarsiiform/exp OR Catarrhini/de OR Platyrrhini/exp OR ape/de OR Cercopithecidae/exp OR hominid/de OR hylobatidae/exp OR chimpanzee/exp OR gorilla/exp OR 'orang utan'/exp OR (animal OR animals OR pisces OR fish OR fishes OR catfish OR catfishes OR sheatfish OR silurus OR arius OR heteropneustes OR clarias OR gariepinus OR "fathead minnow" OR "fathead minnows" OR pimephales OR promelas OR cichlidae OR trout OR trouts OR char OR chars OR salvelinus OR salmo OR oncorhynchus OR guppy OR guppies OR millionfish OR poecilia OR goldfish OR goldfishes OR carassius OR auratus OR mullet OR mullets OR mugil OR curema OR shark OR sharks OR cod OR cods OR gadus OR morhua OR carp OR carps OR cyprinus OR carpio OR killifish OR eel OR eels OR anguilla OR zander OR sander OR lucioperca OR stizostedion OR turbot OR turbots OR psetta OR flatfish OR flatfishes OR plaice OR pleuronectes OR platessa OR tilapia OR tilapias OR oreochromis OR sarotherodon OR "common sole" OR "dover sole" OR solea OR zebrafish OR zebrafishes OR danio OR rerio OR seabass OR dicentrarchus OR labrax OR morone OR lamprey OR lampreys OR petromyzon OR pumpkinseed OR pumpkinseeds OR lepomis OR gibbosus OR herring OR clupea OR harengus OR amphibia OR amphibian OR amphibians OR anura OR salientia OR frog OR frogs OR rana OR toad OR toads OR bufo OR xenopus OR laevis OR bombina OR epidalea OR calamita OR salamander OR salamanders OR newt OR newts OR triturus OR reptilia OR reptile OR reptiles OR "bearded dragon" OR pogona OR vitticeps OR iguana OR iguanas OR lizard OR lizards OR "anguis fragilis" OR turtle OR turtles OR snakes OR snake OR aves OR bird OR birds OR quail OR quails OR coturnix OR bobwhite OR colinus OR virginianus OR poultry OR poultries OR fowl OR fowls OR chicken OR chickens OR gallus OR "zebra finch" OR taeniopygia OR guttata OR canary OR canaries OR serinus OR canaria OR parakeet OR parakeets OR grasskeet OR parrot OR parrots OR psittacine OR psittacines OR shelduck OR tadorna OR goose OR geese OR branta OR leucopsis OR woodlark OR lullula OR flycatcher OR ficedula OR hypoleuca OR dove OR doves OR geopelia OR cuneata OR duck OR ducks OR greylag OR graylag OR anser OR harrier OR "circus pygargus" OR "red knot" OR "great knot" OR calidris OR canutus OR godwit OR limosa OR lapponica OR meleagris OR gallopavo OR jackdaw OR corvus OR monedula OR ruff OR philomachus OR pugnax OR lapwing OR peewit OR plover OR vanellus OR swan OR cygnus OR columbianus OR bewickii OR gull OR chroicocephalus OR ridibundus OR albifrons OR "great tit" OR parus OR aythya OR fuligula OR streptopelia OR risoria OR spoonbill OR platalea OR leucorodia OR blackbird OR turdus OR merula OR "blue tit" OR cyanistes OR pigeon OR pigeons OR columba OR

pintail OR anas OR starling OR sturnus OR owl OR "athene noctua" OR pochard OR ferina OR cockatiel OR nymphicus OR hollandicus OR skylark OR alauda OR tern OR sterna OR teal OR crecca OR oystercatcher OR haematopus OR ostralegus OR shrew OR shrews OR sorex OR araneus OR crocidura OR russula OR "european mole" OR talpa OR chiroptera OR bat OR bats OR eptesicus OR serotinus OR myotis OR dasycneme OR daubentonii OR pipistrelle OR pipistrellus OR cat OR cats OR felis OR catus OR feline OR dog OR dogs OR canis OR canine OR canines OR otter OR otters OR lutra OR badger OR badgers OR meles OR fitchew OR fitch OR foumart or foulmart OR ferrets OR ferret OR polecat OR polecats OR mustela OR putorius OR weasel OR weasels OR fox OR foxes OR vulpes OR "common seal" OR phoca OR vitulina OR "grey seal" OR halichoerus OR horse OR horses OR equus OR equine OR equidae OR donkey OR donkeys OR mule OR mules OR pig OR pigs OR swine OR swines OR hog OR hogs OR boar OR boars OR porcine OR piglet OR piglets OR sus OR scrofa OR llama OR llamas OR lama OR glama OR deer OR deers OR cervus OR elaphus OR cow OR cows OR "bos taurus" OR "bos indicus" OR bovine OR bull OR bulls OR cattle OR bison OR bisons OR sheep OR sheeps OR "ovis aries" OR ovine OR lamb OR lambs OR mouflon OR mouflons OR goat OR goats OR capra OR caprine OR chamois OR rupicapra OR leporidae OR lagomorpha OR lagomorph OR rabbit OR rabbits OR oryctolagus OR cuniculus OR laprine OR hares OR lepus OR rodentia OR rodent OR rodents OR murinae OR mouse OR mice OR mus OR musculus OR murine OR woodmouse OR apodemus OR rat OR rats OR rattus OR norvegicus OR "guinea pig" OR "guinea pigs" OR cavia OR porcellus OR hamster OR hamsters OR mesocricetus OR cricetulus OR cricetus OR gerbil OR gerbils OR jird OR jirds OR meriones OR unguiculatus OR jerboa OR jerboas OR jaculus OR chinchilla OR chinchillas OR beaver OR beavers OR "castor fiber" OR "castor canadensis" OR sciuridae OR squirrel OR squirrels OR sciurus OR chipmunk OR chipmunks OR marmot OR marmots OR marmota OR suslik OR susliks OR spermophilus OR cynomys OR cottonrat OR cottonrats OR sigmodon OR vole OR voles OR microtus OR myodes OR glareolus OR primate OR primates OR prosimian OR prosimians OR lemur OR lemurs OR lemuridae OR loris OR "bush baby" OR "bush babies" OR bushbaby OR bushbabies OR galago OR galagos OR anthropoidea OR anthropoids OR simian OR simians OR monkey OR monkeys OR marmoset OR marmosets OR callithrix OR cebuella OR tamarin OR tamarins OR saguinus OR leontopithecus OR "squirrel monkey" OR "squirrel monkeys" OR saimiri OR "night monkey" OR "night monkeys" OR "owl monkey" OR "owl monkeys" OR douroucoulis OR aotus OR "spider monkey" OR "spider monkeys" OR ateles OR baboon OR baboons OR papio OR "rhesus monkey" OR macaque OR macaca OR mulatta OR cynomolgus OR fascicularis OR "green monkey" OR "green monkeys" OR chlorocebus OR vervet OR vervets OR pygerythrus OR hominoidea OR ape OR apes OR hylobatidae OR gibbon OR gibbons OR siamang OR siamangs OR nomascus OR symphalangus OR hominidae OR orangutan OR orangutans OR pongo OR chimpanzee OR chimpanzees OR "pan troglodytes" OR bonobo OR bonobos OR "pan paniscus" OR gorilla OR gorillas OR troglodytes):ab,ti)

Medline

<u>Cuprizone</u>

(Cuprizone/ or (Cuprizone or Cuprizon or Cuprizane or Cupferazone or biscycloxaldihydrazone).ab,ti. or (bicyclohexanone adj3 oxalyldihydrazone).ab,ti.) and (Myelin Sheath/ or exp Demyelinating Diseases/ or exp Oligodendroglia/ or exp Central Nervous System/ or (myelin or myelinic body or remyelination or demyelination or oligodendrocyte* or oligodendroglia or CNS or central nervous system).ab,ti. or (cerebrospinal adj3 axi*).ab,ti. or (spinal* adj3 (cord* or medulla or marrow)).ab,ti. or spinalcord*.ab,ti.) AND exp animal experimentation/ or exp models, animal/ or exp invertebrates/ or Animals/ or exp animal population groups/ or chordata/ or exp chordata, nonvertebrate/ or vertebrates/ or exp amphibians/ or exp birds/ or exp fishes/ or exp reptiles/ or mammals/ or primates/ or exp artiodactyla/ or exp carnivora/ or exp cetacea/ or exp chiroptera/ or exp elephants/ or exp

hyraxes/ or exp insectivora/ or exp lagomorpha/ or exp marsupialia/ or exp monotremata/ or exp perissodactyla/ or exp rodentia/ or exp scandentia/ or exp sirenia/ or exp xenarthra/ or haplorhini/ or exp strepsirhini/ or exp platyrrhini/ or exp tarsii/ or catarrhini/ or exp cercopithecidae/ or exp hylobatidae/ or hominidae/ or exp gorilla gorilla/ or exp pan paniscus/ or exp pan troglodytes/ or exp pongo pygmaeus/ or ((animals or animal or mice or mus or mouse or murine or woodmouse or rats or rat or murinae or muridae or cottonrat or cottonrats or hamster or hamsters or cricetinae or rodentia or rodent or rodents or pigs or pig or swine or swines or piglets or piglet or boar or boars or "sus scrofa" or ferrets or ferret or polecat or polecats or "mustela putorius" or "guinea pigs" or "guinea pig" or cavia or callithrix or marmoset or marmosets or cebuella or hapale or octodon or chinchilla or chinchillas or gerbillinae or gerbil or gerbils or jird or jirds or merione or meriones or rabbits or rabbit or hares or hare or diptera or flies or fly or dipteral or drosophila or drosophilidae or cats or cat or carus or felis or nematoda or nematode or nematodes or sipunculida or dogs or dog or canine or canines or canis or sheep or sheeps or mouflon or mouflons or ovis or goats or goat or capra or capras or rupicapra or rupicapras or chamois or haplorhini or monkey or monkeys or anthropoidea or anthropoids or saguinus or tamarin or tamarins or leontopithecus or hominidae or ape or apes or "pan paniscus" or bonobo or bonobos or "pan troglodytes" or gibbon or gibbons or siamang or siamangs or nomascus or symphalangus or chimpanzee or chimpanzees or prosimian or prosimians or "bush baby" or bush babies or galagos or galago or pongidae or gorilla or gorillas or "pongo pygmaeus" or orangutan or orangutans or lemur or lemurs or lemuridae or horse or horses or equus or cow or calf or bull or chicken or chickens or gallus or quail or bird or birds or quails or poultry or poultries or fowl or fowls or reptile or reptilia or reptiles or snakes or snake or lizard or lizards or alligator or alligators or crocodile or crocodiles or turtle or turtles or amphibian or amphibians or amphibia or frog or frogs or bombina or salientia or toad or toads or "epidalea calamita" or salamander or salamanders or eel or eels or fish or fishes or pisces or catfish or catfishes or siluriformes or arius or heteropneustes or sheatfish or perch or perches or percidae or perca or trout or trouts or char or chars or salvelinus or minnow or cyprinidae or carps or carp or zebrafish or zebrafishes or goldfish or goldfishes or guppy or guppies or chub or chubs or tinca or barbels or barbus or pimephales or promelas or "poecilia reticulata" or mullet or mullets or eel or eels or seahorse or seahorses or mugil curema or atlantic cod or shark or sharks or catshark or anguilla or salmonid or salmonids or whitefish or whitefishes or salmon or salmons or sole or solea or lamprey or lampreys or pumpkinseed or sunfish or sunfishes or tilapia or tilapias or turbot or turbots or flatfish or flatfishes or sciuridae or squirrel or squirrels or chipmunk or chipmunks or suslik or susliks or vole or voles or lemming or lemmings or muskrat or muskrats or lemmus or otter or otters or marten or martens or martes or weasel or badger or badgers or ermine or mink or minks or sable or sables or gulo or gulos or wolverine or wolverines or mustela or llama or llamas or alpaca or alpacas or camelid or camelids or guanaco or guanacos or chiroptera or chiropteras or bat or bats or fox or foxes or iguana or iguanas or xenopus laevis or parakeet or parakeets or parrot or parrots or donkey or donkeys or mule or mules or zebra or zebras or shrew or shrews or bison or bisons or buffalo or buffaloes or deer or deers or bear or bears or panda or pandas or "wild hog" or "wild boar" or fitchew or fitch or beaver or beavers or jerboa or jerboas or capybara or capybaras).ti,ab. not medline.st.)

Ethidium bromide

(Ethidium/ or etbr.ab,ti. or homidium.ab,ti. or ((ethidium or homidium or novidium) adj3 (hydrobromide or bromide)).ab,ti.) and (Myelin Sheath/ or exp Demyelinating Diseases/ or exp Oligodendroglia/ or exp Central Nervous System/ or (myelin or myelinic body or remyelination or demyelination or oligodendrocyte* or oligodendroglia or CNS or central nervous system).ab,ti. or (cerebrospinal adj3 axi*).ab,ti. or (spinal* adj3 (cord* or medulla or marrow)).ab,ti. or spinalcord*.ab,ti.) AND exp animal experimentation/ or exp models, animal/ or exp invertebrates/ or

Animals/ or exp animal population groups/ or chordata/ or exp chordata, nonvertebrate/ or vertebrates/ or exp amphibians/ or exp birds/ or exp fishes/ or exp reptiles/ or mammals/ or primates/ or exp artiodactyla/ or exp carnivora/ or exp cetacea/ or exp chiroptera/ or exp elephants/ or exp hyraxes/ or exp insectivora/ or exp lagomorpha/ or exp marsupialia/ or exp monotremata/ or exp perissodactyla/ or exp rodentia/ or exp scandentia/ or exp sirenia/ or exp xenarthra/ or haplorhini/ or exp strepsirhini/ or exp platyrrhini/ or exp tarsii/ or catarrhini/ or exp cercopithecidae/ or exp hylobatidae/ or hominidae/ or exp gorilla gorilla/ or exp pan paniscus/ or exp pan troglodytes/ or exp pongo pygmaeus/ or ((animals or animal or mice or mus or mouse or murine or woodmouse or rats or rat or murinae or muridae or cottonrat or cottonrats or hamster or hamsters or cricetinae or rodentia or rodent or rodents or pigs or pig or swine or swines or piglets or piglet or boar or boars or "sus scrofa" or ferrets or ferret or polecat or polecats or "mustela putorius" or "guinea pigs" or "guinea pig" or cavia or callithrix or marmoset or marmosets or cebuella or hapale or octodon or chinchilla or chinchillas or gerbillinae or gerbil or gerbils or jird or jirds or merione or meriones or rabbits or rabbit or hares or hare or diptera or flies or fly or dipteral or drosophila or drosophilidae or cats or cat or carus or felis or nematoda or nematode or nematodes or sipunculida or dogs or dog or canine or canines or canis or sheep or sheeps or mouflon or mouflons or ovis or goats or goat or capra or capras or rupicapra or rupicapras or chamois or haplorhini or monkey or monkeys or anthropoidea or anthropoids or saguinus or tamarin or tamarins or leontopithecus or hominidae or ape or apes or "pan paniscus" or bonobo or bonobos or "pan troglodytes" or gibbon or gibbons or siamang or siamangs or nomascus or symphalangus or chimpanzee or chimpanzees or prosimian or prosimians or "bush baby" or bush babies or galagos or galago or pongidae or gorilla or gorillas or "pongo pygmaeus" or orangutan or orangutans or lemur or lemurs or lemuridae or horse or horses or equus or cow or calf or bull or chicken or chickens or gallus or quail or bird or birds or quails or poultry or poultries or fowl or fowls or reptile or reptilia or reptiles or snakes or snake or lizard or lizards or alligator or alligators or crocodile or crocodiles or turtle or turtles or amphibian or amphibians or amphibia or frog or frogs or bombina or salientia or toad or toads or "epidalea calamita" or salamander or salamanders or eel or eels or fish or fishes or pisces or catfish or catfishes or siluriformes or arius or heteropneustes or sheatfish or perch or perches or percidae or perca or trout or trouts or char or chars or salvelinus or minnow or cyprinidae or carps or carp or zebrafish or zebrafishes or goldfish or goldfishes or guppy or guppies or chub or chubs or tinca or barbels or barbus or pimephales or promelas or "poecilia reticulata" or mullet or mullets or eel or eels or seahorse or seahorses or mugil curema or atlantic cod or shark or sharks or catshark or anguilla or salmonid or salmonids or whitefish or whitefishes or salmon or salmons or sole or solea or lamprey or lampreys or pumpkinseed or sunfish or sunfishes or tilapia or tilapias or turbot or turbots or flatfish or flatfishes or sciuridae or squirrel or squirrels or chipmunk or chipmunks or suslik or susliks or vole or voles or lemming or lemmings or muskrat or muskrats or lemmus or otter or otters or marten or martens or martes or weasel or badger or badgers or ermine or mink or minks or sable or sables or gulo or gulos or wolverine or wolverines or mustela or llama or llamas or alpaca or alpacas or camelid or camelids or guanaco or guanacos or chiroptera or chiropteras or bat or bats or fox or foxes or iguana or iguanas or xenopus laevis or parakeet or parakeets or parrot or parrots or donkey or donkeys or mule or mules or zebra or zebras or shrew or shrews or bison or bisons or buffalo or buffaloes or deer or deers or bear or bears or panda or pandas or "wild hog" or "wild boar" or fitchew or fitch or beaver or beavers or jerboa or jerboas or capybara or capybaras).ti,ab. not medline.st.)

<u>Lysolecithin</u>

(Lysophosphatidylcholines/ or lysophosphatidylcholine*.ab,ti. Or lysolecithin*.ab,ti. or (choline adj3 lysophosphatid*).ab,ti. Or (phosphatidylcholine adj3 lyso).ab,ti.) and (Myelin Sheath/ or exp Demyelinating Diseases/ or exp Oligodendroglia/ or exp Central Nervous System/ or (myelin or

myelinic body or remyelination or demyelination or oligodendrocyte* or oligodendroglia or CNS or central nervous system).ab,ti. or (cerebrospinal adj3 axi*).ab,ti. or (spinal* adj3 (cord* or medulla or marrow)).ab,ti. or spinalcord*.ab,ti.) AND exp animal experimentation/ or exp models, animal/ or exp invertebrates/ or Animals/ or exp animal population groups/ or chordata/ or exp chordata, nonvertebrate/ or vertebrates/ or exp amphibians/ or exp birds/ or exp fishes/ or exp reptiles/ or mammals/ or primates/ or exp artiodactyla/ or exp carnivora/ or exp cetacea/ or exp chiroptera/ or exp elephants/ or exp hyraxes/ or exp insectivora/ or exp lagomorpha/ or exp marsupialia/ or exp monotremata/ or exp perissodactyla/ or exp rodentia/ or exp scandentia/ or exp sirenia/ or exp xenarthra/ or haplorhini/ or exp strepsirhini/ or exp platyrrhini/ or exp tarsii/ or catarrhini/ or exp cercopithecidae/ or exp hylobatidae/ or hominidae/ or exp gorilla gorilla/ or exp pan paniscus/ or exp pan troglodytes/ or exp pongo pygmaeus/ or ((animals or animal or mice or mus or mouse or murine or woodmouse or rats or rat or murinae or muridae or cottonrat or cottonrats or hamster or hamsters or cricetinae or rodentia or rodent or rodents or pigs or pig or swine or swines or piglets or piglet or boar or boars or "sus scrofa" or ferrets or ferret or polecat or polecats or "mustela putorius" or "guinea pigs" or "guinea pig" or cavia or callithrix or marmoset or marmosets or cebuella or hapale or octodon or chinchilla or chinchillas or gerbillinae or gerbil or gerbils or jird or jirds or merione or meriones or rabbits or rabbit or hares or hare or diptera or flies or fly or dipteral or drosophila or drosophilidae or cats or cat or carus or felis or nematoda or nematode or nematodes or sipunculida or dogs or dog or canine or canines or canis or sheep or sheeps or mouflon or mouflons or ovis or goats or goat or capra or capras or rupicapra or rupicapras or chamois or haplorhini or monkey or monkeys or anthropoidea or anthropoids or saguinus or tamarin or tamarins or leontopithecus or hominidae or ape or apes or "pan paniscus" or bonobo or bonobos or "pan troglodytes" or gibbon or gibbons or siamang or siamangs or nomascus or symphalangus or chimpanzee or chimpanzees or prosimian or prosimians or "bush baby" or bush babies or galagos or galago or pongidae or gorilla or gorillas or "pongo pygmaeus" or orangutan or orangutans or lemur or lemurs or lemuridae or horse or horses or equus or cow or calf or bull or chicken or chickens or gallus or quail or bird or birds or quails or poultry or poultries or fowl or fowls or reptile or reptilia or reptiles or snakes or snake or lizard or lizards or alligator or alligators or crocodile or crocodiles or turtle or turtles or amphibian or amphibians or amphibia or frog or frogs or bombina or salientia or toad or toads or "epidalea calamita" or salamander or salamanders or eel or eels or fish or fishes or pisces or catfish or catfishes or siluriformes or arius or heteropneustes or sheatfish or perch or perches or percidae or perca or trout or trouts or char or chars or Salvelinus or minnow or cyprinidae or carps or carp or zebrafish or zebrafishes or goldfish or goldfishes or guppy or guppies or chub or chubs or tinca orbarbels or barbus or pimephales or promelas or "poecilia reticulata" or mullet or mullets or eel or eels or seahorse or seahorses or mugil curema or atlantic cod or shark or sharks or catshark or anguilla or salmonid or salmonids or whitefish or whitefishes or salmon or salmons or sole or solea or lamprey or lampreys or pumpkinseed or sunfish or sunfishes or tilapia or tilapias or turbot or turbots or flatfish or flatfishes or sciuridae or squirrel or squirrels or chipmunk or chipmunks or suslik or susliks or vole or voles or lemming or lemmings or muskrat or muskrats or lemmus or otter or otters or marten or martens or martes or weasel or badger or badgers or ermine or mink or minks or sable or sables or gulo or gulos or wolverine or wolverines or mustela or llama or llamas or alpaca or alpacas or camelid or camelids or guanaco or guanacos or chiroptera or chiropteras or bat or bats or fox or foxes or iguana or iguanas or xenopus laevis or parakeet or parakeets or parrot or parrots or donkey or donkeys or mule or mules or zebra or zebras or shrew or shrews or bison or bisons or buffalo or buffaloes or deer or deers or bear or bears or panda or pandas or "wild hog" or "wild boar" or fitchew or fitch or beaver or beavers or jerboa or jerboas or capybara or capybaras).ti,ab. not medline.st.)

Anti-galactocerebroside antibodies/complement

(anti-galactocerebroside* or antigalactocerebroside* or "anti galactocerebroside" or "galactocerebroside antibody" or "galactocerebroside antibodies" or anti-GalC or "anti-GalC" or "GalC antibody" or "GalC antibodies" or anti-galactosylceramide* or "anti galactosylceramide" or "anti galactosylceramides" or (anti* adj6 ("acylsphingosine galactoside" or "ceramide galactoside" or cerebrogalactoside* or galactocerebroside* or "galactosyl acylsphingosine" or "galactosyl ceramide" or galactosylceramide*))).ti,ab. and (Myelin Sheath/ or exp Demyelinating Diseases/ or exp Oligodendroglia/ or exp Central Nervous System/ or (myelin or myelinic body or remyelination or demyelination or oligodendrocyte* or oligodendroglia or CNS or central nervous system).ab,ti. or (cerebrospinal adj3 axi*).ab,ti. or (spinal* adj3 (cord* or medulla or marrow)).ab,ti. or spinalcord*.ab,ti.).af. AND (anti-galactocerebroside* or antigalactocerebroside* or "anti galactocerebroside" or "galactocerebroside antibody" or "galactocerebroside antibodies" or anti-GalC or "anti-GalC" or "GalC antibody" or "GalC antibodies" or anti-galactosylceramide* or "anti galactosylceramide" or "anti galactosylceramides" or (anti* adj6 ("acylsphingosine galactoside" or "ceramide galactoside" or cerebrogalactoside* or galactocerebroside* or "galactosyl acylsphingosine" or "galactosyl ceramide" or galactosylceramide*))).ti,ab. and (Myelin Sheath/ or exp Demyelinating Diseases/ or exp Oligodendroglia/ or exp Central Nervous System/ or (myelin or myelinic body or remyelination or demyelination or oligodendrocyte* or oligodendroglia or CNS or central nervous system).ab,ti. or (cerebrospinal adj3 axi*).ab,ti. or (spinal* adj3 (cord* or medulla or marrow)).ab,ti. or spinalcord*.ab,ti.).af.

PubMed

Cuprizone

Search (anti-galactocerebroside*[Tiab] OR antigalactocerebroside*[Tiab] OR "anti galactocerebroside"[Tiab] OR "galactocerebroside antibody"[Tiab] OR "galactocerebroside antibodies"[Tiab] OR anti-GalC[Tiab] OR "anti-GalC"[Tiab] OR "GalC antibody"[Tiab] OR "GalC antibodies"[Tiab] OR antigalactosylceramide[Tiab] OR "anti galactosylceramide"[Tiab] OR "anti galactosylceramide"[Tiab]) OR (("anti-"[Tiab] OR antibody[Tiab] OR antibodies[Tiab] OR antiserum[Tiab])) AND (("acylsphingosine galactoside"[Tiab] OR "ceramide galactoside"[Tiab] OR cerebrogalactoside*[Tiab] OR galactocerebroside*[Tiab] OR "galactosyl acylsphingosine"[Tiab] OR "galactosyl ceramide"[Tiab] OR galactosylceramide*[Tiab])) AND ((myelin[tiab] OR remyelination[tiab] OR demyelination[tiab] OR oligodendrocyte[tiab] OR oligodendrocytes[tiab] OR oligodendroglia[tiab] OR CNS[tiab] OR "central nervous system"[tiab]) OR (cerebrospinal[tiab] AND (axi[tiab] OR axis[tiab])) OR ((spinal[tiab] OR spinalis[tiab]) AND (cord[tiab] OR cords[tiab] OR medulla[tiab] OR marrow[tiab])) OR spinalcord[tiab] OR spinalcord[tiab])

Ethidium bromide

Search (((((etbr[tiab] OR homidium[tiab] OR lysophosphatidylcholines[tiab] OR ((ethidium[tiab] OR homidium[tiab] OR novidium[tiab]) AND (hydrobromide[tiab] OR bromide[tiab]))))) AND ((AND (myelin[tiab] OR remyelination[tiab] OR demyelination[tiab] OR oligodendrocyte[tiab] OR oligodendrocytes[tiab] OR oligodendroglia[tiab] OR CNS[tiab] OR "central nervous system" [tiab]) OR (cerebrospinal[tiab] AND (axi[tiab] OR axis[tiab])) OR ((spinal[tiab] OR spinalis[tiab]) AND (cord[tiab] OR cords[tiab] OR medulla[tiab] OR marrow[tiab])) OR spinalcord[tiab] OR spinalcord[tiab])))) AND (((inprocess[sb])) OR (publisher[sb] NOT pubstatusnihms NOT pubstatuspmcsd NOT pmcbook))

Lysolecithin

Search ((((inprocess[sb])) OR (publisher[sb] NOT pubstatusnihms NOT pubstatuspmcsd NOT pmcbook))) AND (((("animal experimentation"[MeSH Terms] OR "models, animal"[MeSH Terms] OR "invertebrates"[MeSH Terms] OR "Animals"[Mesh:noexp] OR "animal population groups"[MeSH Terms] OR "chordata"[MeSH Terms:noexp] OR "chordata, nonvertebrate"[MeSH Terms] OR "vertebrates"[MeSH Terms:noexp] OR "amphibians"[MeSH Terms] OR "birds" [MeSH Terms] OR "fishes"[MeSH Terms] OR "reptiles"[MeSH Terms] OR "mammals"[MeSH Terms:noexp] OR "primates"[MeSH Terms:noexp] OR "artiodactyla"[MeSH Terms] OR "carnivora"[MeSH Terms] OR "cetacea"[MeSH Terms] OR "chiroptera"[MeSH Terms] OR "elephants"[MeSH Terms] OR "hyraxes"[MeSH Terms] OR "insectivora"[MeSH Terms] OR "lagomorpha"[MeSH Terms] OR "marsupialia"[MeSH Terms] OR "monotremata"[MeSH Terms] OR "perissodactyla"[MeSH Terms] OR "rodentia"[MeSH Terms] OR "scandentia" [MeSH Terms] OR "sirenia"[MeSH Terms] OR "xenarthra"[MeSH Terms] OR "haplorhini"[MeSH Terms:noexp] OR "strepsirhini"[MeSH Terms] OR "platyrrhini" [MeSH Terms] OR "tarsii"[MeSH Terms] OR "catarrhini"[MeSH Terms:noexp] OR "cercopithecidae"[MeSH Terms] OR "hylobatidae"[MeSH Terms] OR "hominidae" [MeSH Terms:noexp] OR "gorilla gorilla"[MeSH Terms] OR "pan paniscus"[MeSH Terms] OR "pan troglodytes"[MeSH Terms] OR "pongo pygmaeus"[MeSH Terms]) OR ((animals[tiab] OR animal[tiab] OR mice[Tiab] OR mus[Tiab] OR mouse[Tiab] OR murine[Tiab] OR woodmouse[tiab] OR rats[Tiab] OR rat[Tiab] OR murinae[Tiab] OR muridae[Tiab] OR cottonrat[tiab] OR cottonrats[tiab] OR hamster[tiab] OR hamsters[tiab] OR cricetinae[tiab] OR rodentia[Tiab] OR rodent[Tiab] OR rodents[Tiab] OR pigs[Tiab] OR pig[Tiab] OR swine[tiab] OR swines[tiab] OR piglets[tiab] OR piglet[tiab] OR boar[tiab] OR boars[tiab] OR "sus scrofa"[tiab] OR ferrets[tiab] OR ferret[tiab] OR polecat[tiab] OR polecats[tiab] OR "mustela putorius"[tiab] OR "guinea pigs"[Tiab] OR "guinea pig"[Tiab] OR cavia[Tiab] OR callithrix[Tiab] OR marmoset[Tiab] OR marmosets[Tiab] OR cebuella[Tiab] OR hapale[Tiab] OR octodon[Tiab] OR chinchilla[Tiab] OR chinchillas[Tiab] OR gerbillinae[Tiab] OR gerbil[Tiab] OR gerbils[Tiab] OR jird[Tiab] OR jirds[Tiab] OR merione[Tiab] OR meriones[Tiab] OR rabbits[Tiab] OR rabbit[Tiab] OR hares[Tiab] OR hare[Tiab] OR diptera[Tiab] OR flies[Tiab] OR fly[Tiab] OR dipteral[Tiab] OR drosophila[Tiab] OR drosophilidae[Tiab] OR cats[Tiab] OR cat[Tiab] OR carus[Tiab] OR felis[Tiab] OR nematoda[Tiab] OR nematode[Tiab] OR nematodes[Tiab] OR sipunculida[Tiab] OR dogs[Tiab] OR dog[Tiab] OR canine[Tiab] OR canines[Tiab] OR canis[Tiab] OR sheep[Tiab] OR sheeps[Tiab] OR mouflon[Tiab] OR mouflons[Tiab] OR ovis[Tiab] OR goats[Tiab] OR goat[Tiab] OR capra[Tiab] OR capras[Tiab] OR rupicapra[Tiab] OR rupicapras[Tiab] OR chamois[Tiab] OR haplorhini[Tiab] OR monkey[Tiab] OR monkeys[Tiab] OR anthropoidea[Tiab] OR anthropoids[Tiab] OR saguinus[Tiab] OR tamarin[Tiab] OR tamarins[Tiab] OR leontopithecus[Tiab] OR hominidae[Tiab] OR ape[Tiab] OR apes[Tiab] OR "pan paniscus"[Tiab] OR bonobo[Tiab] OR bonobos[Tiab] OR "pan troglodytes"[Tiab] OR gibbon[Tiab] OR gibbons[Tiab] OR siamang[Tiab] OR siamangs[Tiab] OR nomascus[Tiab] OR symphalangus[Tiab] OR chimpanzee[Tiab] OR chimpanzees[Tiab] OR prosimian[Tiab] OR prosimians[Tiab] OR "bush baby"[Tiab] OR bush babies[Tiab] OR galagos[Tiab] OR galago[Tiab] OR pongidae[Tiab] OR gorilla[Tiab] OR gorillas[Tiab] OR "pongo pygmaeus"[Tiab] OR orangutan[Tiab] OR orangutans[Tiab] OR lemur[Tiab] OR lemurs[Tiab] OR lemuridae[Tiab] OR horse[Tiab] OR horses[Tiab] OR equus[Tiab] OR cow[Tiab] OR calf[Tiab] OR bull[Tiab] OR chicken[Tiab] OR chickens[Tiab] OR gallus[Tiab] OR quail[Tiab] OR bird[Tiab] OR birds[Tiab] OR quails[Tiab] OR poultry[Tiab] OR poultries[Tiab] OR fowl[Tiab] OR fowls[Tiab] OR reptile[Tiab] OR reptilia[Tiab] OR reptiles[Tiab] OR snakes[Tiab] OR snake[Tiab] OR lizard[Tiab] OR lizards[Tiab] OR alligator[Tiab] OR alligators[Tiab] OR crocodile[Tiab] OR crocodiles[Tiab] OR turtle[Tiab] OR turtles[Tiab] OR amphibian[Tiab] OR amphibians[Tiab] OR amphibia[Tiab] OR frog[Tiab] OR frogs[Tiab] OR bombina[Tiab] OR salientia[Tiab] OR toad[Tiab] OR toads[Tiab] OR "epidalea calamita"[Tiab] OR salamander[Tiab] OR salamanders[Tiab] OR eel[Tiab] OR eels[Tiab] OR fish[Tiab] OR fishes[Tiab] OR pisces[Tiab] OR catfish[Tiab] OR

catfishes[Tiab] OR siluriformes[Tiab] OR arius[Tiab] OR heteropneustes[Tiab] OR sheatfish[Tiab] OR perch[Tiab] OR perches[Tiab] OR percidae[Tiab] OR perca[Tiab] OR trout[Tiab] OR trouts[Tiab] OR char[Tiab] OR chars[Tiab] OR salvelinus[Tiab] OR minnow[Tiab] OR cyprinidae[Tiab] OR carps[Tiab] OR carp[Tiab] OR zebrafish[Tiab] OR zebrafishes[Tiab] OR goldfish[Tiab] OR goldfishes[Tiab] OR guppy[Tiab] OR guppies[Tiab] OR chub[Tiab] OR chubs[Tiab] OR tinca[Tiab] OR barbels[Tiab] OR barbus[Tiab] OR pimephales[Tiab] OR promelas[Tiab] OR "poecilia reticulata"[Tiab] OR mullet[Tiab] OR mullets[Tiab] OR eel[Tiab] OR eels[Tiab] OR seahorse[Tiab] OR seahorses[Tiab] OR mugil curema[Tiab] OR atlantic cod[Tiab] OR shark[Tiab] OR sharks[Tiab] OR catshark[Tiab] OR anguilla[Tiab] OR salmonid[Tiab] OR salmonids[Tiab] OR whitefish[Tiab] OR whitefishes[Tiab] OR salmon[Tiab] OR salmons[Tiab] OR sole[Tiab] OR solea[Tiab] OR lamprey[Tiab] OR lampreys[Tiab] OR pumpkinseed[Tiab] OR sunfish[Tiab] OR sunfishes[Tiab] OR tilapia[Tiab] OR tilapias[Tiab] OR turbot[Tiab] OR turbots[Tiab] OR flatfish[Tiab] OR flatfishes[Tiab] OR sciuridae[Tiab] OR squirrel[Tiab] OR squirrels[Tiab] OR chipmunk[Tiab] OR chipmunks[Tiab] OR suslik[Tiab] OR susliks[Tiab] OR vole[Tiab] OR voles[Tiab] OR lemming[Tiab] OR lemmings[Tiab] OR muskrat[Tiab] OR muskrats[Tiab] OR lemmus[Tiab] OR otter[Tiab] OR otters[Tiab] OR marten[Tiab] OR martens[Tiab] OR martes[Tiab] OR weasel[Tiab] OR badger[Tiab] OR badgers[Tiab] OR ermine[Tiab] OR mink[Tiab] OR minks[Tiab] OR sable[Tiab] OR sables[Tiab] OR gulo[Tiab] OR gulos[Tiab] OR wolverine[Tiab] OR wolverines[Tiab] OR mustela[Tiab] OR llama[Tiab] OR llamas[Tiab] OR alpaca[Tiab] OR alpacas[Tiab] OR camelid[Tiab] OR camelids[Tiab] OR guanaco[Tiab] OR guanacos[Tiab] OR chiroptera[Tiab] OR chiropteras[Tiab] OR bat[Tiab] OR bats[Tiab] OR fox[Tiab] OR foxes[Tiab] OR iguana[Tiab] OR iguanas[Tiab] OR xenopus laevis[Tiab] OR parakeet[Tiab] OR parakeets[Tiab] OR parrot[Tiab] OR parrots[Tiab] OR donkey[Tiab] OR donkeys[Tiab] OR mule[Tiab] OR mules[Tiab] OR zebra[Tiab] OR zebras[Tiab] OR shrew[Tiab] OR shrews[Tiab] OR bison[Tiab] OR bisons[Tiab] OR buffalo[Tiab] OR buffaloes[Tiab] OR deer[Tiab] OR deers[Tiab] OR bear[Tiab] OR bears[Tiab] OR panda[Tiab] OR pandas[Tiab] OR "wild hog"[Tiab] OR "wild boar"[Tiab] OR fitchew[Tiab] OR fitch[Tiab] OR beaver[Tiab] OR beavers[Tiab] OR jerboa[Tiab] OR jerboas[Tiab] OR capybara[Tiab] OR capybaras[Tiab]) NOT medline[sb]))) AND ((((lysophosphatidylcholine[tiab] OR lysolecithin[tiab] OR lysophosphatidylcholines[tiab] OR lysolecithine[tiab] OR (choline[tiab] AND (lysophosphatide[tiab] OR lysophosphatidal[tiab] OR lysophosphatidyl[tiab])) OR (phosphatidylcholine[tiab] AND lyso[tiab])))) AND ((myelin[tiab] OR "myelinic body"[tiab] OR remyelination[tiab] OR demyelination[tiab] OR oligodendrocyte[tiab] OR oligodendrocytes[tiab] OR oligodendroglia[tiab] OR CNS[tiab] OR "central nervous system"[tiab]) OR (cerebrospinal[tiab] AND (axi[tiab] OR axis[tiab])) OR ((spinal[tiab] OR spinalis[tiab]) AND (cord[tiab] OR cords[tiab] OR medulla[tiab] OR marrow[tiab])) OR spinalcord[tiab] OR spinalcord[tiab]))))

<u>Anti-galactocerebroside antibodies/complement</u>

Search (((anti-galactocerebroside*[Tiab] OR antigalactocerebroside*[Tiab] OR "anti galactocerebroside"[Tiab] OR "galactocerebroside antibody"[Tiab] OR "galactocerebroside antibodies"[Tiab] OR anti-GalC[Tiab] OR "anti-GalC"[Tiab] OR "GalC antibody"[Tiab] OR "GalC antibodies"[Tiab] OR antigalactosylceramide[Tiab] OR "anti galactosylceramide"[Tiab] OR "anti galactosylceramide"[Tiab]) OR (("anti-"[Tiab] OR antibody[Tiab] OR antibodies[Tiab] OR antiserum[Tiab])) AND (("acylsphingosine galactoside"[Tiab] OR "ceramide galactoside"[Tiab] OR cerebrogalactoside*[Tiab] OR galactocerebroside*[Tiab] OR "galactosyl acylsphingosine"[Tiab] OR "galactosyl ceramide"[Tiab] OR galactosylceramide*[Tiab]))) AND ((myelin[tiab] OR remyelination[tiab] OR demyelination[tiab] OR oligodendrocyte[tiab] OR oligodendrocytes[tiab] OR oligodendroglia[tiab] OR CNS[tiab] OR "central nervous system"[tiab]) OR (cerebrospinal[tiab] AND (axi[tiab] OR axis[tiab])) OR ((spinal[tiab] OR spinalis[tiab]) AND (cord[tiab] OR cords[tiab] OR medulla[tiab] OR marrow[tiab]))

OR spinalcord[tiab] OR spinalcord[tiab]))) AND ((((inprocess[sb])) OR (publisher[sb] NOT pubstatusnihms NOT pubstatuspmcsd NOT pmcbook)))

Scopus

Cuprizone

(TITLE-ABS-KEY ((cuprizone OR cuprizon OR cuprizane OR cupferazone OR biscycloxaldihydrazone OR (bicyclohexanone W/3 oxalyldihydrazone)) AND (myelin OR "myelinic body" OR remyelination OR demyelination OR oligodendrocyte* OR oligodendroglia OR cns OR "central nervous system" OR (cerebrospinal W/3 axi*) OR (spinal* W/3 (cord* OR medulla OR marrow)) OR spinalcord*))) AND (TITLE-ABS-KEY (animal OR animals OR pisces OR fish OR fishes OR catfish OR catfishes OR sheatfish OR silurus OR arius OR heteropneustes OR clarias OR gariepinus OR "fathead minnow" OR "fathead minnows" OR pimephales OR promelas OR cichlidae OR trout OR trouts OR char OR chars OR Salvelinus OR salmo OR oncorhynchus OR guppy OR guppies OR millionfish OR poecilia OR goldfish OR goldfishes OR carassius OR auratus OR mullet OR mullets OR mugil OR curema OR shark OR sharks OR cod OR cods OR gadus OR morhua OR carp OR carps OR cyprinus OR carpio OR killifish OR eel OR eels OR anguilla OR zander OR sander OR lucioperca OR stizostedion OR turbot OR turbots OR psetta OR flatfish OR flatfishes OR plaice OR pleuronectes OR platessa OR tilapia OR tilapias OR oreochromis OR sarotherodon OR "common sole" OR "dover sole" OR solea OR zebrafish OR zebrafishes OR danio OR rerio OR seabass OR dicentrarchus OR labrax OR morone OR lamprey OR lampreys OR Petromyzon OR pumpkinseed OR pumpkinseeds OR lepomis OR gibbosus OR herring OR clupea OR harengus OR amphibia OR amphibian OR amphibians OR anura OR salientia OR frog OR frogs OR rana OR toad OR toads OR bufo OR xenopus OR laevis OR bombina OR epidalea OR calamita OR salamander OR salamanders OR newt OR newts OR triturus OR reptilia OR reptile OR reptiles OR "bearded dragon" OR pogona OR vitticeps OR iguana OR iguanas OR lizard OR lizards OR "anguis fragilis" OR turtle OR turtles OR snakes OR snake OR aves OR bird OR birds OR quail OR quails OR coturnix OR bobwhite OR colinus OR virginianus OR poultry OR poultries OR fowl OR fowls OR chicken OR chickens OR gallus OR "zebra finch" OR taeniopygia OR guttata OR canary OR canaries OR serinus OR canaria OR parakeet OR parakeets OR grasskeet OR parrot OR parrots OR psittacine OR psittacines OR shelduck OR tadorna OR goose OR geese OR branta OR leucopsis OR woodlark OR lullula OR flycatcher OR ficedula OR hypoleuca OR dove OR doves OR geopelia OR cuneata OR duck OR ducks OR greylag OR graylag OR anser OR harrier OR "circus pygargus" OR "red knot" OR "great knot" OR calidris OR canutus OR godwit OR limosa OR lapponica OR meleagris OR gallopavo OR jackdaw OR corvus OR monedula OR ruff OR philomachus OR pugnax OR lapwing OR peewit OR plover OR vanellus OR swan OR cygnus OR columbianus OR bewickii OR gull OR chroicocephalus OR ridibundus OR albifrons OR "great tit" OR parus OR aythya OR fuligula OR streptopelia OR risoria OR spoonbill OR platalea OR leucorodia OR blackbird OR turdus OR merula OR "blue tit" OR cyanistes OR pigeon OR pigeons OR columba OR pintail OR anas OR starling OR sturnus OR owl OR "athene noctua" OR pochard OR ferina OR cockatiel OR nymphicus OR hollandicus OR skylark OR alauda OR tern OR sterna OR teal OR crecca OR oystercatcher OR haematopus OR ostralegus OR shrew OR shrews OR sorex OR araneus OR crocidura OR russula OR "european mole" OR talpa OR chiroptera OR bat OR bats OR eptesicus OR serotinus OR myotis OR dasycneme OR daubentonii OR pipistrelle OR pipistrellus OR cat OR cats OR felis OR catus OR feline OR dog OR dogs OR canis OR canine OR canines OR otter OR otters OR lutra OR badger OR badgers OR meles OR fitchew OR fitch OR foumart OR foulmart OR ferrets OR ferret OR polecat OR polecats OR Mustela OR putorius OR weasel OR weasels OR fox OR foxes OR vulpes OR "common seal" OR phoca OR vitulina OR "grey seal" OR halichoerus OR horse OR horses OR equus OR equine OR equidae OR donkey OR donkeys OR mule OR mules OR pig OR pigs OR swine OR swines OR hog OR hogs OR boar OR boars OR porcine OR piglet OR piglets OR sus OR scrofa OR llama OR llamas OR lama OR glama OR

deer OR deers OR cervus OR elaphus OR cow OR cows OR "bos taurus" OR "bos indicus" OR bovine OR bull OR bulls OR cattle OR bison OR bisons OR sheep OR sheeps OR "ovis aries" OR ovine OR lamb OR lambs OR mouflon OR mouflons OR goat OR goats OR capra OR caprine OR chamois OR rupicapra OR leporidae OR lagomorpha OR lagomorph OR rabbit OR rabbits OR oryctolagus OR cuniculus OR laprine OR hares OR lepus OR rodentia OR rodent OR rodents OR murinae OR mouse OR mice OR mus OR musculus OR murine OR woodmouse OR apodemus OR rat OR rats OR rattus OR norvegicus OR "guinea pig" OR "guinea pigs" OR cavia OR porcellus OR hamster OR hamsters OR mesocricetus OR cricetulus OR cricetus OR gerbil OR gerbils OR jird OR jirds OR meriones OR unguiculatus OR jerboa OR jerboas OR jaculus OR chinchilla OR chinchillas OR beaver OR beavers OR "castor fiber" OR "castor canadensis" OR Sciuridae OR squirrel OR squirrels OR sciurus OR chipmunk OR chipmunks OR marmot OR marmots OR marmota OR suslik OR susliks OR spermophilus OR cynomys OR cottonrat OR cottonrats OR sigmodon OR vole OR voles OR microtus OR myodes OR glareolus OR primate OR primates OR prosimian OR prosimians OR lemur OR lemurs OR lemuridae OR loris OR "bush baby" OR "bush babies" OR bushbaby OR bushbabies OR galago OR galagos OR anthropoidea OR anthropoids OR simian OR simians OR monkey OR monkeys OR marmoset OR marmosets OR callithrix OR cebuella OR tamarin OR tamarins OR saguinus OR leontopithecus OR "squirrel monkey" OR "squirrel monkeys" OR saimiri OR "night monkey" OR "night monkeys" OR "owl monkey" OR "owl monkeys" OR douroucoulis OR aotus OR "spider monkey" OR "spider monkeys" OR ateles OR baboon OR baboons OR papio OR "rhesus monkey" OR macaque OR macaca OR mulatta OR cynomolgus OR fascicularis OR "green monkey" OR "green monkeys" OR chlorocebus OR vervet OR vervets OR pygerythrus OR hominoidea OR ape OR apes OR hylobatidae OR gibbon OR gibbons OR siamang OR siamangs OR nomascus OR symphalangus OR hominidae OR orangutan OR orangutans OR pongo OR chimpanzee OR chimpanzees OR "pan troglodytes" OR bonobo OR bonobos OR "pan paniscus" OR gorilla OR gorillas OR troglodytes))

Ethidium bromide

TITLE-ABS-KEY (etbr OR homidium OR ((ethidium OR homidium OR novidium W/3 (hydrobromide OR bromide))) AND (myelin OR "myelinic body" OR remyelination OR demyelination OR oligodendrocyte* OR oligodendroglia OR cns OR "central nervous system" OR (cerebrospinal W/3 axi*) OR (spinal* W/3 (cord* OR medulla OR marrow)) OR spinalcord*))) AND (TITLE-ABS-KEY (animal OR animals OR pisces OR fish OR fishes OR catfish OR catfishes OR sheatfish OR silurus OR arius OR heteropneustes OR clarias OR gariepinus OR "fathead minnow" OR "fathead minnows" OR pimephales OR promelas OR cichlidae OR trout OR trouts OR char OR chars OR salvelinus OR salmo OR Oncorhynchus OR guppy OR guppies OR millionfish OR poecilia OR goldfish OR goldfishes OR carassius OR auratus OR mullet OR mullets OR mugil OR curema OR shark OR sharks OR cod OR cods OR gadus OR morhua OR carp OR carps OR cyprinus OR carpio OR killifish OR eel OR eels OR anguilla OR zander OR sander OR lucioperca OR stizostedion OR turbot OR turbots OR psetta OR flatfish OR flatfishes OR plaice OR pleuronectes OR platessa OR tilapia OR tilapias OR oreochromis OR sarotherodon OR "common sole" OR "dover sole" OR solea OR zebrafish OR zebrafishes OR danio OR rerio OR seabass OR dicentrarchus OR labrax OR morone OR lamprey OR lampreys OR petromyzon OR pumpkinseed OR pumpkinseeds OR lepomis OR gibbosus OR herring OR clupea OR harengus OR amphibia OR amphibian OR amphibians OR anura OR salientia OR frog OR frogs OR rana OR toad OR toads OR bufo OR xenopus OR laevis OR bombina OR epidalea OR calamita OR salamander OR salamanders OR newt OR newts OR triturus OR reptilia OR reptile OR reptiles OR "bearded dragon" OR pogona OR vitticeps OR iguana OR iguanas OR lizard OR lizards OR "anguis fragilis" OR turtle OR turtles OR snakes OR snake OR aves OR bird OR birds OR quail OR quails OR coturnix OR bobwhite OR colinus OR virginianus OR poultry OR poultries OR fowl OR fowls OR chicken OR chickens OR gallus OR "zebra finch" OR taeniopygia OR

guttata OR canary OR canaries OR serinus OR canaria OR parakeet OR parakeets OR grasskeet OR parrot OR parrots OR psittacine OR psittacines OR shelduck OR tadorna OR goose OR geese OR branta OR leucopsis OR woodlark OR lullula OR flycatcher OR ficedula OR hypoleuca OR dove OR doves OR geopelia OR cuneata OR duck OR ducks OR greylag OR graylag OR anser OR harrier OR "circus pygargus" OR "red knot" OR "great knot" OR calidris OR canutus OR godwit OR limosa OR lapponica OR meleagris OR gallopavo OR jackdaw OR corvus OR monedula OR ruff OR philomachus OR pugnax OR lapwing OR peewit OR plover OR vanellus OR swan OR cygnus OR columbianus OR bewickii OR gull OR chroicocephalus OR ridibundus OR albifrons OR "great tit" OR parus OR aythya OR fuligula OR streptopelia OR risoria OR spoonbill OR platalea OR leucorodia OR blackbird OR turdus OR merula OR "blue tit" OR cyanistes OR pigeon OR pigeons OR columba OR pintail OR anas OR starling OR sturnus OR owl OR "athene noctua" OR pochard OR ferina OR cockatiel OR nymphicus OR hollandicus OR skylark OR alauda OR tern OR sterna OR teal OR crecca OR oystercatcher OR haematopus OR ostralegus OR shrew OR shrews OR sorex OR araneus OR crocidura OR russula OR "european mole" OR talpa OR chiroptera OR bat OR bats OR eptesicus OR serotinus OR myotis OR dasycneme OR daubentonii OR pipistrelle OR pipistrellus OR cat OR cats OR felis OR catus OR feline OR dog OR dogs OR canis OR canine OR canines OR otter OR otters OR lutra OR badger OR badgers OR meles OR fitchew OR fitch OR foumart OR foulmart OR ferrets OR ferret OR polecat OR polecats OR mustela OR putorius OR weasel OR weasels OR fox OR foxes OR vulpes OR "common seal" OR phoca OR vitulina OR "grey seal" OR halichoerus OR horse OR horses OR equus OR equine OR equidae OR donkey OR donkeys OR mule OR mules OR pig OR pigs OR swine OR swines OR hog OR hogs OR boar OR boars OR porcine OR piglet OR piglets OR sus OR scrofa OR llama OR llamas OR lama OR glama OR deer OR deers OR cervus OR elaphus OR cow OR cows OR "bos taurus" OR "bos indicus" OR bovine OR bull OR bulls OR cattle OR bison OR bisons OR sheep OR sheeps OR "ovis aries" OR ovine OR lamb OR lambs OR mouflon OR mouflons OR goat OR goats OR capra OR caprine OR chamois OR rupicapra OR leporidae OR lagomorpha OR lagomorph OR rabbit OR rabbits OR oryctolagus OR cuniculus OR laprine OR hares OR lepus OR rodentia OR rodent OR rodents OR murinae OR mouse OR mice OR mus OR musculus OR murine OR woodmouse OR apodemus OR rat OR rats OR rattus OR norvegicus OR "guinea pig" OR "guinea pigs" OR cavia OR porcellus OR hamster OR hamsters OR mesocricetus OR cricetulus OR cricetus OR gerbil OR gerbils OR jird OR jirds OR meriones OR unguiculatus OR jerboa OR jerboas OR jaculus OR chinchilla OR chinchillas OR beaver OR beavers OR "castor fiber" OR "castor canadensis" OR sciuridae OR squirrel OR squirrels OR sciurus OR chipmunk OR chipmunks OR marmot OR marmots OR marmota OR suslik OR susliks OR spermophilus OR cynomys OR cottonrat OR cottonrats OR sigmodon OR vole OR voles OR microtus OR myodes OR glareolus OR primate OR primates OR prosimian OR prosimians OR lemur OR lemurs OR lemuridae OR loris OR "bush baby" OR "bush babies" OR bushbaby OR bushbabies OR galago OR galagos OR anthropoidea OR anthropoids OR simian OR simians OR monkey OR monkeys OR marmoset OR marmosets OR callithrix OR cebuella OR tamarin OR tamarins OR saguinus OR leontopithecus OR "squirrel monkey" OR "squirrel monkeys" OR saimiri OR "night monkey" OR "night monkeys" OR "owl monkey" OR "owl monkeys" OR douroucoulis OR aotus OR "spider monkey" OR "spider monkeys" OR ateles OR baboon OR baboons OR papio OR "rhesus monkey" OR macaque OR macaca OR mulatta OR cynomolgus OR fascicularis OR "green monkey" OR "green monkeys" OR chlorocebus OR vervet OR vervets OR pygerythrus OR hominoidea OR ape OR apes OR hylobatidae OR gibbon OR gibbons OR siamang OR siamangs OR nomascus OR symphalangus OR Hominidae OR orangutan OR orangutans OR pongo OR chimpanzee OR chimpanzees OR "pantroglodytes" OR bonobo OR bonobos OR "pan paniscus" OR gorilla OR gorillas OR troglodytes))

<u>Lysolecithin</u>

TITLE-ABS-KEY ((lysophosphatidylcholine* OR lysolecithin* OR (choline W/3 lysophosphatid*) OR (phosphatidylcholine W/3 lyso)) AND (myelin OR "myelinic body" OR remyelination OR demyelination OR oligodendrocyte* OR oligodendroglia OR cns OR "central nervous system" OR (cerebrospinal W/3 axi*) OR (spinal* W/3 (cord* OR medulla OR marrow)) OR spinalcord*))) AND (TITLE-ABS-KEY (animal OR animals OR pisces OR fish OR fishes OR catfish OR catfishes OR sheatfish OR silurus OR arius OR heteropneustes OR clarias OR gariepinus OR "fathead minnow" OR "fathead minnows" OR pimephales OR promelas OR cichlidae OR trout OR trouts OR char OR chars OR salvelinus OR salmo OR Oncorhynchus OR guppy OR guppies OR millionfish OR poecilia OR goldfish OR goldfishes OR carassius OR auratus OR mullet OR mullets OR mugil OR curema OR shark OR sharks OR cod OR cods OR gadus OR morhua OR carp OR carps OR cyprinus OR carpio OR killifish OR eel OR eels OR anguilla OR zander OR sander OR lucioperca OR stizostedion OR turbot OR turbots OR psetta OR flatfish OR flatfishes OR plaice OR pleuronectes OR platessa OR tilapia OR tilapias OR oreochromis OR sarotherodon OR "common sole" OR "dover sole" OR solea OR zebrafish OR zebrafishes OR danio OR rerio OR seabass OR dicentrarchus OR labrax OR morone OR lamprey OR lampreys OR petromyzon OR pumpkinseed OR pumpkinseeds OR lepomis OR gibbosus OR herring OR clupea OR harengus OR amphibia OR amphibian OR amphibians OR anura OR salientia OR frog OR frogs OR rana OR toad OR toads OR bufo OR xenopus OR laevis OR bombina OR epidalea OR calamita OR salamander OR salamanders OR newt OR newts OR triturus OR reptilia OR reptile OR reptiles OR "bearded dragon" OR pogona OR vitticeps OR iguana OR iguanas OR lizard OR lizards OR "anguis fragilis" OR turtle OR turtles OR snakes OR snake OR aves OR bird OR birds OR quail OR quails OR coturnix OR bobwhite OR colinus OR virginianus OR poultry OR poultries OR fowl OR fowls OR chicken OR chickens OR gallus OR "zebra finch" OR taeniopygia OR guttata OR canary OR canaries OR serinus OR canaria OR parakeet OR parakeets OR grasskeet OR parrot OR parrots OR psittacine OR psittacines OR shelduck OR tadorna OR goose OR geese OR branta OR leucopsis OR woodlark OR lullula OR flycatcher OR ficedula OR hypoleuca OR dove OR doves OR geopelia OR cuneata OR duck OR ducks OR greylag OR graylag OR anser OR harrier OR "circus pygargus" OR "red knot" OR "great knot" OR calidris OR canutus OR godwit OR limosa OR lapponica OR meleagris OR gallopavo OR jackdaw OR corvus OR monedula OR ruff OR philomachus OR pugnax OR lapwing OR peewit OR plover OR vanellus OR swan OR cygnus OR columbianus OR bewickii OR gull OR chroicocephalus OR ridibundus OR albifrons OR "great tit" OR parus OR aythya OR fuligula OR streptopelia OR risoria OR spoonbill OR platalea OR leucorodia OR blackbird OR turdus OR merula OR "blue tit" OR cyanistes OR pigeon OR pigeons OR columba OR pintail OR anas OR starling OR sturnus OR owl OR "athene noctua" OR pochard OR ferina OR cockatiel OR nymphicus OR hollandicus OR skylark OR alauda OR tern OR sterna OR teal OR crecca OR oystercatcher OR haematopus OR ostralegus OR shrew OR shrews OR sorex OR araneus OR crocidura OR russula OR "european mole" OR talpa OR chiroptera OR bat OR bats OR eptesicus OR serotinus OR myotis OR dasycneme OR daubentonii OR pipistrelle OR pipistrellus OR cat OR cats OR felis OR catus OR feline OR dog OR dogs OR canis OR canine OR canines OR otter OR otters OR lutra OR badger OR badgers OR meles OR fitchew OR fitch OR foumart OR foulmart OR ferrets OR ferret OR polecat OR polecats OR mustela OR putorius OR weasel OR weasels OR fox OR foxes OR vulpes OR "common seal" OR phoca OR vitulina OR "grey seal" OR halichoerus OR horse OR horses OR equus OR equine OR equidae OR donkey OR donkeys OR mule OR mules OR pig OR pigs OR swine OR swines OR hog OR hogs OR boar OR boars OR porcine OR piglet OR piglets OR sus OR scrofa OR llama OR llamas OR lama OR glama OR deer OR deers OR cervus OR elaphus OR cow OR cows OR "bos taurus" OR "bos indicus" OR bovine OR bull OR bulls OR cattle OR bison OR bisons OR sheep OR sheeps OR "ovis aries" OR ovine OR lamb OR lambs OR mouflon OR mouflons OR goat OR goats OR capra OR caprine OR chamois OR rupicapra OR leporidae OR lagomorpha OR lagomorph OR rabbit OR rabbits OR oryctolagus OR cuniculus OR laprine OR hares OR lepus OR rodentia OR rodent OR rodents OR murinae OR mouse OR mice OR mus OR musculus OR murine OR woodmouse OR apodemus OR rat OR rats OR rattus OR norvegicus OR "guinea pig" OR "guinea pigs" OR cavia OR porcellus OR hamster OR hamsters OR mesocricetus OR cricetulus OR cricetus OR gerbil

OR gerbils OR jird OR jirds OR meriones OR unguiculatus OR jerboa OR jerboas OR jaculus OR chinchilla OR chinchillas OR beaver OR beavers OR "castor fiber" OR "castor canadensis" OR sciuridae OR squirrel OR squirrels OR sciurus OR chipmunk OR chipmunks OR marmot OR marmots OR marmota OR suslik OR susliks OR spermophilus OR cynomys OR cottonrat OR cottonrats OR sigmodon OR vole OR voles OR microtus OR myodes OR glareolus OR primate OR primates OR prosimian OR prosimians OR lemur OR lemurs OR lemuridae OR loris OR "bush baby" OR "bush babies" OR bushbaby OR bushbabies OR galago OR galagos OR anthropoidea OR anthropoids OR simian OR simians OR monkey OR monkeys OR marmoset OR marmosets OR callithrix OR cebuella OR tamarin OR tamarins OR saguinus OR leontopithecus OR "squirrel monkey" OR "squirrel monkeys" OR saimiri OR "night monkey" OR "night monkeys" OR "owl monkey" OR "owl monkeys" OR douroucoulis OR aotus OR "spider monkey" OR "spider monkeys" OR ateles OR baboon OR baboons OR papio OR "rhesus monkey" OR macaque OR macaca OR mulatta OR cynomolgus OR fascicularis OR "green monkey" OR "green monkeys" OR chlorocebus OR vervet OR vervets OR pygerythrus OR hominoidea OR ape OR apes OR hylobatidae OR gibbon OR gibbons OR siamang OR siamangs OR nomascus OR symphalangus OR Hominidae OR orangutan OR orangutans OR pongo OR chimpanzee OR chimpanzees OR "pan troglodytes" OR bonobo OR bonobos OR "pan paniscus" OR gorilla OR gorillas OR troglodytes))

<u>Anti-galactocerebroside antibodies/complement</u>

TITLE-ABS-KEY (animal OR animals OR pisces OR fish OR fishes OR catfish OR catfishes OR sheatfish OR silurus OR arius OR heteropneustes OR clarias OR gariepinus OR "fathead minnow" OR "fathead minnows" OR pimephales OR promelas OR cichlidae OR trout OR trouts OR char OR chars OR salvelinus OR salmo OR oncorhynchus OR guppy OR guppies OR millionfish OR poecilia OR goldfish OR goldfishes OR carassius OR auratus OR mullet OR mullets OR mugil OR curema OR shark OR sharks OR cod OR cods OR gadus OR morhua OR carp OR carps OR cyprinus OR carpio OR killifish OR eel OR eels OR anguilla OR zander OR sander OR lucioperca OR stizostedion OR turbot OR turbots OR psetta OR flatfish OR flatfishes OR plaice OR pleuronectes OR platessa OR tilapia OR tilapias OR oreochromis OR sarotherodon OR "common sole" OR "dover sole" OR solea OR zebrafish OR zebrafishes OR danio OR rerio OR seabass OR dicentrarchus OR labrax OR morone OR lamprey OR lampreys OR petromyzon OR pumpkinseed OR pumpkinseeds OR lepomis OR gibbosus OR herring OR clupea OR harengus OR amphibia OR amphibian OR amphibians OR anura OR salientia OR frog OR frogs OR rana OR toad OR toads OR bufo OR xenopus OR laevis OR bombina OR epidalea OR calamita OR salamander OR salamanders OR newt OR newts OR triturus OR reptilia OR reptile OR reptiles OR "bearded dragon" OR pogona OR vitticeps OR iguana OR iguanas OR lizard OR lizards OR "anguis fragilis" OR turtle OR turtles OR snakes OR snake OR aves OR bird OR birds OR quail OR quails OR coturnix OR bobwhite OR colinus OR virginianus OR poultry OR poultries OR fowl OR fowls OR chicken OR chickens OR gallus OR "zebra finch" OR taeniopygia OR guttata OR canary OR canaries OR serinus OR canaria OR parakeet OR parakeets OR grasskeet OR parrot OR parrots OR psittacine OR psittacines OR shelduck OR tadorna OR goose OR geese OR branta OR leucopsis OR woodlark OR lullula OR flycatcher OR ficedula OR hypoleuca OR dove OR doves OR geopelia OR cuneata OR duck OR ducks OR greylag OR graylag OR anser OR harrier OR "circus pygargus" OR "red knot" OR "great knot" OR calidris OR canutus OR godwit OR limosa OR lapponica OR meleagris OR gallopavo OR jackdaw OR corvus OR monedula OR ruff OR philomachus OR pugnax OR lapwing OR peewit OR plover OR vanellus OR swan OR cygnus OR columbianus OR bewickii OR gull OR chroicocephalus OR ridibundus OR albifrons OR "great tit" OR parus OR aythya OR fuligula OR streptopelia OR risoria OR spoonbill OR platalea OR leucorodia OR blackbird OR turdus OR merula OR "blue tit" OR cyanistes OR pigeon OR pigeons OR columba OR pintail OR anas OR starling OR sturnus OR owl OR "athene noctua" OR pochard OR ferina OR cockatiel OR nymphicus OR hollandicus OR skylark OR alauda OR tern OR sterna OR teal OR crecca OR oystercatcher OR

haematopus OR ostralegus OR shrew OR shrews OR sorex OR araneus OR crocidura OR russula OR "european mole" OR talpa OR chiroptera OR bat OR bats OR eptesicus OR serotinus OR myotis OR dasycneme OR daubentonii OR pipistrelle OR pipistrellus OR cat OR cats OR felis OR catus OR feline OR dog OR dogs OR canis OR canine OR canines OR otter OR otters OR lutra OR badger OR badgers OR meles OR fitchew OR fitch OR foumart OR foulmart OR ferrets OR ferret OR polecat OR polecats OR mustela OR putorius OR weasel OR weasels OR fox OR foxes OR vulpes OR "common seal" OR phoca OR vitulina OR "grey seal" OR halichoerus OR horse OR horses OR equus OR equine OR equidae OR donkey OR donkeys OR mule OR mules OR pig OR pigs OR swine OR swines OR hog OR hogs OR boar OR boars OR porcine OR piglet OR piglets OR sus OR scrofa OR llama OR llamas OR lama OR glama OR deer OR deers OR cervus OR elaphus OR cow OR cows OR "bos taurus" OR "bos indicus" OR bovine OR bull OR bulls OR cattle OR bison OR bisons OR sheep OR sheeps OR "ovis aries" OR ovine OR lamb OR lambs OR mouflon OR mouflons OR goat OR goats OR capra OR caprine OR chamois OR rupicapra OR leporidae OR lagomorpha OR lagomorph OR rabbit OR rabbits OR oryctolagus OR cuniculus OR laprine OR hares OR lepus OR rodentia OR rodent OR rodents OR murinae OR mouse OR mice OR mus OR musculus OR murine OR woodmouse OR apodemus OR rat OR rats OR rattus OR norvegicus OR "guinea pig" OR "guinea pigs" OR cavia OR porcellus OR hamster OR hamsters OR mesocricetus OR cricetulus OR cricetus OR gerbil OR gerbils OR jird OR jirds OR meriones OR unguiculatus OR jerboa OR jerboas OR jaculus OR chinchilla OR chinchillas OR beaver OR beavers OR "castor fiber" OR "castor canadensis" OR sciuridae OR squirrel OR squirrels OR sciurus OR chipmunk OR chipmunks OR marmot OR marmots OR marmota OR suslik OR susliks OR spermophilus OR Cynomys OR cottonrat OR cottonrats OR sigmodon OR vole OR voles OR microtus OR myodes OR glareolus OR primate OR primates OR prosimian OR prosimians OR lemur OR lemurs OR lemuridae OR loris OR "bush baby" OR "bush babies" OR bushbaby OR bushbabies OR galago OR galagos OR anthropoidea OR anthropoids OR simian OR simians OR monkey OR monkeys OR marmoset OR marmosets OR callithrix OR cebuella OR tamarin OR tamarins OR saguinus OR leontopithecus OR "squirrel monkey" OR "squirrel monkeys" OR saimiri OR "night monkey" OR "night monkeys" OR "owl monkey" OR "owl monkeys" OR douroucoulis OR aotus OR "spider monkey" OR "spider monkeys" OR ateles OR baboon OR baboons OR papio OR "rhesus monkey" OR macaque OR macaca OR mulatta OR cynomolgus OR fascicularis OR "green monkey" OR "green monkeys" OR chlorocebus OR vervet OR vervets OR pygerythrus OR hominoidea OR ape OR apes OR hylobatidae OR gibbon OR gibbons OR siamang OR siamangs OR nomascus OR symphalangus OR hominidae OR orangutan OR orangutans OR pongo OR chimpanzee OR chimpanzees OR "pan troglodytes" OR bonobo OR bonobos OR "pan paniscus" OR gorilla OR gorillas OR troglodytes)) AND (TITLE-ABS-KEY (((antigalactocerebroside* OR antigalactocerebroside* OR "anti galactocerebroside" OR "galactocerebroside antibody" OR "galactocerebroside antibodies" OR anti-galc OR "anti-GalC" OR "GalC antibody" OR "GalC antibodies" OR anti-galactosylceramide* OR "antigalactosylceramide" OR "anti galactosylceramides") OR (anti* W/6 ("acylsphingosine galactoside" OR "ceramide galactoside" OR cerebrogalactoside* OR galactocerebroside* OR "galactosyl acylsphingosine" OR "galactosyl ceramide" OR galactosylceramide*))) AND (myelin OR "myelinic body" OR remyelination OR demyelination OR oligodendrocyte* OR oligodendroglia OR cns OR "central nervous system" OR (cerebrospinal W/3 axi*) OR (spinal* W/3 (cord* OR medulla OR marrow)) OR spinalcord*)))

Animal filter according to[1,2].

Search string References

1 de Vries, R. B., Hooijmans, C. R., Tillema, A., Leenaars, M. & Ritskes-Hoitinga, M. Updated version of the Embase search filter for animal studies. *Laboratory animals* **48**, 88, doi:10.1177/0023677213494374 (2014).

2 Hooijmans, C. R., Tillema, A., Leenaars, M. & Ritskes-Hoitinga, M. Enhancing search efficiency by means of a search filter for finding all studies on animal experimentation in PubMed. *Laboratory animals* **44**, 170-175, doi:10.1258/la.2010.009117 (2010).

Therapy	First author	Year	Model	Species	Sex	Remyelination compared to control	Oligodendrocyte cell counts compared to control	OPC cell counts compared to control	Latest Phase of clinical trials
4-hydroxyquinazolin (PARP inhibitor)	Veto et al.	2010	Cuprizone	Mouse	Male	Increase	No data	No data	-
AH6809 (EP2 antagonist)	Palumbo et al.	2012	Cuprizone	Mouse	Male	Increase	No data	No data	-
AL-8810 (Prostaglandin F2 alpha agonist)	Iwasa et al.	2014	Cuprizone	Mouse	Male	Increase	No data	Increase	-
Amphotericin B (antimycotic)	Döring et al.	2015	LPC	Mouse	Female	Increase	No data	Decrease	-
Androstenediol	Kalakh et al.	2015	EB	Rat	Male	Increase	Increase	Increase	-
Anti-Lingo-1 antibody	Mi et al.	2009	LPC	Rat	Not reported	Increase	No data	No data	2
Anti-Lingo-1 antibody	Zhang et al.	2015	LPC	Mouse	Female	Increase	No data	No data	2
Anti-Lingo-1 antibody	Hu et al.	2011	LPC	Rat	Not reported	Equal	No data	No data	2
Anti-Lingo-1 antibody	Pepinsky et al.	2011	LPC	Rat	Female	Increase	No data	No data	2
Anti-Lingo-1 antibody	Pepinsky et al.	2011	LPC	Rat	Female	Increase	No data	No data	2
Anti-Lingo-1 antibody	Mi et al.	2009	Cuprizone	Mouse	Not reported	Increase	Decrease	No data	2
Apotransferrin	Aparicio et al.	2013	LPC	Rat	Mixed	No data	Increase	Decrease	-
Apotransferrin	Adamo et al.	2006	Cuprizone	Rat	Mixed	Increase	Increase	No data	-
Areca catechu nut extract (ANE)	Adilijiang et al.	2015	Cuprizone	Mouse	Male	Increase	No data	Decrease	-
Benztropine	Deshmukh et al.	2013	Cuprizone	Rat	Female	Increase	Increase	No data	-
Bucladesine (phosphodiesterase inhibitor)	Vakilzadeh et al.	2015	Cuprizone	Rat	Male	Increase	No data	No data	-
Calcitriol (1,25-dihydroxyvitamin D3)	Nystad et al.	2014	Cuprizone	Mouse	Female	Increase	Increase	No data	4
CCX771 (CXCR7 antagonist)	Williams et al.	2014	Cuprizone	Mouse	Male	Increase	Decrease	Increase	-
Celecoxib (Selective COX-2 inhibitor)	Palumbo et al.	2012	Cuprizone	Mouse	Male	Increase	No data	Decrease	-
Ciliary neurotrophic factor (CNTF)	Talbott et al.	2007	EB	Rat	Not reported	No data	Equal	Equal	-
Clobetasol	Najm et al.	2015	LPC	Mouse	Female	No data	Increase	No data	-
Cloprostenol	Iwasa et al.	2014	Cuprizone	Mouse	Male	Equal	Equal	No data	
17β-estradiol (E2)	Kashani et al.	2012	Cuprizone	Mouse	Male	Increase	Increase	No data	-

Cyclicphosphatidicacid (Phospholipid)	Yamamoto et al.	2014	Cuprizone	Mouse	Male	Increase	No data	No data	-
Cyclosporin	Bondan et al.	2011	EB	Rat	Male	Increase	No data	No data	3
Cyclosporin	Smith et al.	2001	EB	Rat	Female	Equal	No data	No data	3
Cytidine-50-diphospho-choline (CDP choline)	Skripuletz et al.	2015	Cuprizone	Mouse	Not reported	Increase	Increase	No data	-
Dizocilipine (MK801, NMDA receptor antagonist)	Li et al.	2013	Cuprizone	Mouse	Male	Decrease	No data	Increase	-
Dl-3-n-butylphthalide (extract from Apium Graveolens Linn)	Wu et al.	2015	EB	Rat	Male	Increase	No data	No data	-
Dimethyl Fumaric acid ester	Moharregh-Khiabani et al.	2010	Cuprizone	Mouse	Male	Increase	Equal	No data	4
Ebselen (glutaminase inhibitor)	Mazzanti et al.	2009	EB	Rat	Male	Increase	No data	No data	2
Electro-acupuncture	Huang et al.	2011	EB	Rat	Male	Increase	Increase	Increase	2
Electromagnetic field stimulation (EMFs)	Sherafat et al.	2012	LPC	Rat	Female	Increase	No data	No data	2
Electromagnetic field stimulation (EMFs)	Li et al.	2010	EB	Rat	Male	Increase	No data	No data	2
Heparin-binding EGF-like growth factor	Gonzalez-Perez et al.	2009	LPC	Mouse	Not reported	Increase	Increase	No data	-
Epimedium flavonoids (extract of epimedium sagittatum)	Liang et al.	2015	Cuprizone	Mouse	Female	Increase	Increase	Decrease	-
Erythropoietin (EPO)	Hagemeyer et al.	2012	Cuprizone	Mouse	Male	Equal	Equal	No data	2
Estrogen receptor agonist G1	Hirahara et al.	2013	Cuprizone	Rat	Not reported	Increase	No data	No data	-
Fingolimod (Sphingosin-1-phosphate agonist)	Hu et al.	2011	LPC	Rat	Not reported	Decrease	No data	No data	4
Fingolimod	Hu et al.	2011	Cuprizone	Mouse	Not reported	Equal	Equal	Increase	4
Fingolimod	Kim et al.	2011	Cuprizone	Mouse	Male	Equal	Increase	Increase	4
Fingolimod	Slowik et al.	2014	Cuprizone	Mouse	Not reported	Increase	No data	No data	4
Fingolimod	Alme et al.	2015	Cuprizone	Mouse	Female	Equal	Equal	No data	4
Geissoschizine methyl ether (Alkaloid from Uncaria Hook)	Morita et al.	2014	Cuprizone	Mouse	Male	Increase	Increase	No data	-

Glatiramer acetate (mixture of synthetic polypeptides)	Siri et al.	2013	Cuprizone	Rat	Mixed	Increase	Increase	Decrease	4
Glatiramer acetate	Skihar et al.	2009	LPC	Mouse	Male	Increase	No data	No data	4
Growth factor cocktail*	Kumar et al.	2007	Cuprizone	Mouse	Female	Increase	No data	No data	-
Hepatocyte Growth Factor	Bai et al.	2012	LPC	Mouse	Female	No data	No data	Increase	-
Iloprost (prostaglandin I2)	Takahashi et al.	2013	LPC	Mouse	Not reported	Increase	No data	Increase	-
Iloprost	Muramatsu et al.	2015	LPC	Mouse	Female	Increase	No data	No data	-
Indazol chloride (Estrogen receptor agonist)	Moore et al.	2013	Cuprizone	Mouse	Male	Increase	Increase	No data	-
Lactacystin (Proteasome inhibitor)	Millet et al.	2009	Cuprizone	Mouse	Male	Increase	No data	Decrease	-
Laquinimod (Carboxamide derivate)	Brück et al.	2012	Cuprizone	Mouse	Male	Increase	No data	No data	3
L-ascorbic acid 6-hexadecanoate (VCPAL)	Preston et al.	2013	LPC	Mouse	Not reported	No data	Increase	Increase	-
Latanoprost	Iwasa et al.	2014	Cuprizone	Mouse	Male	Decrease	No data	No data	-
Leukemia inhibiting factor (LIF)	Marriott et al.	2008	Cuprizone	Mouse	Not reported	Increase	Increase	No data	-
Macrophage colony-stimulating factor (MCSF)	Döring et al.	2015	LPC	Mouse	Female	Equal	No data	Equal	-
Melatonin	Kashani et al.	2014	Cuprizone	Mouse	Male	Increase	No data	No data	2
Melatonin	Vakilzadeh et al.	2015	Cuprizone	Mouse	Male	Equal	No data	No data	2
Methotrexat (Dihydrofolate reductase inhibitor)	Mueller et al.	2013	Cuprizone	Mouse	Not reported	Equal	No data	No data	4
Methylprednisolone	Cate et al.	2010	Cuprizone	Mouse	Female	Equal	Equal	No data	4
Methylprednisolone	Clarner et al.	2011	Cuprizone	Mouse	Male	Decrease	Equal	No data	4
Methylprednisolone	Chari et al.	2006	EB	Rat	Female	Decrease	Decrease	Equal	4
Methylprednisolone	Pavelko et al.	1998	LPC	Mouse	Not reported	Equal	No data	No data	4
Mexiletine (sodium channel blocker)	Lee et al.	2010	EB	Rat	Male	Increase	No data	No data	-
Miconazol	Najm et al.	2015	LPC	Mouse	Female	No data	Increase	No data	-
Minocycline (Tetracycline)	Li et al.	2005	EB	Rat	Female	Decrease	No data	Decrease	3
Minocycline (Tetracycline)	Skripuletz et al.	2010	Cuprizone	Mouse	Male	Equal	Equal	Equal	3

MK886 (5-Lipoxygenase inhibitor)	Yoshikawa et al.	2011	Cuprizone	Mouse	Male	Equal	No data	No data	-
Monocloncal anti-SCH94.03 IgM-kappa	Pavelko et al.	1998	LPC	Mouse	Not reported	Increase	No data	No data	-
Monomethyl fumaric acid ester	Moharregh-Khiabani et al.	2010	Cuprizone	Mouse	Male	Increase	Equal	No data	-
N6-cyclohexyladenosine (Adenosine A1 receptor agonist)	Asghari et al.	2013	LPC	Rat	Male	Increase	No data	No data	-
Neurotrophin 3 (NT-3)	Jean et al.	2003	LPC	Rat	Male	Increase	Equal	No data	-
Ninjin'yoeito (extract from different medicinal herbs)	Seiwa et al.	2007	Cuprizone	Mouse	Male	Increase	No data	No data	-
Noggin (BMP inhibitor)	Sabo et al.	2011	Cuprizone	Mouse	Female	Increase	Increase	No data	-
Noggin (BMP inhibitor)	Sabo et al.	2013	Cuprizone	Mouse	Not reported	Equal	Increase	No data	-
Olanzapine (Neuroleptic)	Zhang et al.	2014	Cuprizone	Mouse	Female	Increase	Increase	Decrease	-
Olesoxime (cholesterol-like compound)	Magalon et al.	2012	LPC	Mouse	Not reported	Increase	Increase	Equal	1
Olesoxime	Magalon et al.	2012	Cuprizone	Mouse	Not reported	Increase	Increase	No data	1
Olesoxime	Li et al.	2013	Cuprizone	Rat	Female	Increase	No data	No data	1
Omega-3 poly unsaturated fatty acid (PUFA) diet	Torkildsen et al.	2009	Cuprizone	Mouse	Female	Increase	No data	No data	4
Pifithrin-alpha (p53 inhibitor)	Li et al.	2008	Cuprizone	Mouse	Not reported	No data	Increase	No data	-
Pigment epithelium-derived factor (PEDF)	Sohn et al.	2012	LPC	Mouse	Not reported	No data	Increase	Increase	-
Plateled derived growth factor (PDGF)	Allamargot et al.	2001	LPC	Rat	Male	Increase	No data	Increase	-
Polyclonal Ig directed against spinal cord homogenate (anti-SCH-Ig)	Pavelko et al.	1998	LPC	Mouse	Not reported	Increase	No data	No data	-
Polyclonal IgG	Pavelko et al.	1998	LPC	Mouse	Not reported	Increase	No data	No data	-
Progesterone	Ibanez et al.	2004	EB	Rat	Male	Increase	No data	No data	3
Progesterone	Kashani et al.	2015	Cuprizone	Mouse	Male	Increase	Increase	No data	3
Progesterone	El-Etr et al.	2015	Cuprizone	Mouse	Female	Increase	Increase	Increase	3

Quetiapine (atypical antipsychotic)	Xiao et al.	2008	Cuprizone	Mouse	Not reported	Increase	No data	No data	2
Quetiapine	Zhang et al.	2008	Cuprizone	Mouse	Not reported	Increase	Increase	No data	2
Quetiapine	Zhang et al.	2012	Cuprizone	Mouse	Male	Increase	Increase	Decrease	2
Recombinant human Gas6 (rhGas6)	Tsiperson et al.	2010	Cuprizone	Mouse	Male	Increase	No data	No data	-
Recombinant human glial growth factor 2 (RhGGF-2)	Penderis et al.	2003	EB	Rat	Female	Equal	No data	No data	-
Scutellarin (flavonoid extracted from herbal medication)	Wang et al.	2015	Cuprizone	Mouse	Male	Increase	No data	No data	-
Serum derived human polyclonal IgM (sHIgM)	Bieber et al.	2002	LPC	Mouse	Female	Increase	No data	No data	-
Serum-derived human monoclonal IgM14 (sHIgM14)	Bieber et al.	2002	LPC	Mouse	Female	Equal	No data	No data	-
Serum-derived human monoclonal IgM22 (sHIgM22)	Bieber et al.	2002	LPC	Mouse	Female	Increase	No data	No data	1
Serum-derived human polyclonal IgG (sHIgG)	Bieber et al.	2002	LPC	Mouse	Female	Equal	No data	No data	-
Sildenafil (phosphodiesterase inhibitor)	Nunes et al.	2012	Cuprizone	Mouse	Male	Increase	No data	No data	-
Simvastatin	Klopfleisch et al.	2008	Cuprizone	Mouse	Male	Decrease	No data	No data	4
Simvastatin	Miron et al.	2009	Cuprizone	Mouse	Male	Decrease	Decrease	Decrease	4
SiRNA against Nogo-receptor (siNgR)	Pourabdolhossein et al.	2014	LPC	Mouse	Male	Increase	No data	No data	-
Sonic hedgehog (Shh)	Bambakidis et al.	2003	LPC	Rat	Not reported	No data	Increase	No data	-
Testosteron	Hussain et al.	2013	Cuprizone	Mouse	Mixed	Increase	Increase	Increase	2
Thymosin beta4 (Tβ4)	Zhang et al.	2016	Cuprizone	Mouse	Not reported	Increase	Increase	No data	1
Tocopherol derivate TFA-12 (Vitamin E derivate)	Blanchard et al.	2013	LPC	Mouse	Not reported	Increase	Increase	Decrease	-
Trapidil (PDGF agonist)	McKay et al.	1997	LPC	Rat	Not reported	Decrease	No data	No data	-
Travoprost	Iwasa et al.	2014	Cuprizone	Mouse	Male	Equal	No data	No data	-
Triiodothyronine	Franco et al.	2008	Cuprizone	Rat	Not reported	Increase	Increase	No data	1

Triiodothyronine	Harsan et al.	2008	Cuprizone	Mouse	Female	Increase	Increase	No data	1
Triiodothyronine	Silvestroff et al.	2012	Cuprizone	Rat	Not reported	Increase	Increase	No data	1
Triiodothyronine	Zhang et al.	2015	Cuprizone	Mouse	Male	Increase	Increase	Increase	1
Valproic acid	Shen et al.	2008	Cuprizone	Mouse	Male	Decrease	Decrease	Increase	-
Vitamin D (Cholecalciferol)	Nystad et al.	2014	Cuprizone	Mouse	Female	Increase	Increase	No data	4
Vitamin D (Cholecalciferol)	Wergeland et al.	2011	Cuprizone	Mouse	Female	Increase	Equal	No data	4
Vitamin D (Cholecalciferol)	Goudarzvand et al.	2010	EB	Rat	Male	Increase	No data	No data	4
Vitamin E	Mazzanti et al.	2009	EB	Rat	Male	Increase	No data	No data	2
Vitamin E	Goudarzvand et al.	2009	EB	Rat	Male	Increase	No data	No data	2
Vitamin E	Spanevello et al.	2009	EB	Rat	Female	Increase	No data	No data	2

Table 1: Characteristics of the included studies/experiments. Additional information about supposed mode of action or nature of the compound(s) is provided in brackets. Abbreviations: EB, ethidium bromide, LPC, lysolecithin; OPC, oligodendrocyte precursor cell. *The growth factor cocktail contained Platelet derived growth factor-AA, neurotrophin-3, basic fibroblast growth factor, and insulin-like growth factor-1.

Supplementary reference list

Adamo, A. M., P. M. Paez, O. E. Escobar Cabrera, M. Wolfson, P. G. Franco, J. M. Pasquini, and E. F. Soto (2006). Remyelination after cuprizone-induced demyelination in the rat is stimulated by apotransferrin. *Exp. Neurol.*; **198**(2): 519–529.
[DOI:10.1016/j.expneurol.2005.12.027] [PubMed:16480980]

Adilijiang, A., T. Guan, J. He, K. Hartle, W. Wang, and X. Li (2015). The protective effects of Areca catechu extract on cognition and social interaction ceficits in a cuprizone-induced demyelination model. *Evid Based Complement Alternat Med*; **2015**: 426092.
[DOI:10.1155/2015/426092] [PubMed:25815032]

Allamargot, C., A. Pouplard-Barthelaix, and C. Fressinaud (2001). A single intracerebral microinjection of platelet-derived growth factor (PDGF) accelerates the rate of remyelination in vivo. *Brain Res.*; **918**(1-2): 28–39.
[DOI:10.1016/S0006-8993(01)02761-5] [PubMed:11684039]

Alme, M. N., A. E. Nystad, L. Bø, K. M. Myhr, C. A. Vedeler, S. Wergeland, and Ø. Torkildsen (2015). Fingolimod does not enhance cerebellar remyelination in the cuprizone model. *J. Neuroimmunol.*; **285**: 180–186.
[DOI:10.1016/j.jneuroim.2015.06.006] [PubMed:26198937]

Aparicio, E., P. Mathieu, M. Pereira Luppi, M. F. Almeira Gubiani, and A. M. Adamo (2013). The notch signaling pathway: its role in focal CNS demyelination and apotransferrin-induced remyelination. *J. Neurochem.*; **127**(6): 819–836.
[DOI:10.1111/jnc.12440] [PubMed:24032544]

Asghari, A. A., M. Azarnia, J. Mirnajafi-Zadeh, and M. Javan (2013). Adenosine A1 receptor agonist, N6-cyclohexyladenosine, protects myelin and induces remyelination in an experimental model of rat optic chiasm demyelination; electrophysiological and histopathological studies. *J. Neurol. Sci.*; **325**(1-2): 22–28.
[DOI:10.1016/j.jns.2012.11.008] [PubMed:23260322]

Bai, L., D. P. Lennon, A. I. Caplan, A. DeChant, J. Hecker, J. Kranso, A. Zaremba, and R. H. Miller (2012). Hepatocyte growth factor mediates mesenchymal stem cell-induced recovery in multiple sclerosis models. *Nat. Neurosci.*; **15**(6): 862–870.
[DOI:10.1038/nn.3109] [PubMed:22610068]

Bambakidis, N. C., R. Z. Wang, L. Franic, and R. H. Miller (2003). Sonic hedgehog-induced neural precursor proliferation after adult rodent spinal cord injury. *J. Neurosurg.*; **99**(1 Suppl): 70–75.
[DOI:10.3171/spi.2003.99.1.0070] [PubMed:12859063]

Bieber, A. J., A. Warrington, K. Asakura, B. Ciric, S. V. Kaveri, L. R. Pease, and M. Rodriguez (2002). Human antibodies accelerate the rate of remyelination following lysolecithin-induced demyelination in mice. *Glia*; **37**(3): 241–249.
[DOI:10.1002/glia.10033] [PubMed:11857682]

Blanchard, B., T. Heurtaux, C. Garcia, N. M. Moll, C. Caillava, L. Grandbarbe, A. Klosptein, C. Kerninon, M. Frah, D. Coowar, A. Baron-Van Evercooren, E. Morga, P. Heuschling, and B. Nait Oumesmar (2013). Tocopherol derivative TFA-12 promotes myelin repair in experimental models of multiple sclerosis. *J. Neurosci.*; **33**(28): 11633–11642.
[DOI:10.1523/JNEUROSCI.0774-13.2013] [PubMed:23843531]

Bondan, E. F., M. de F. Martins, A. M. Branco, and M. A. Lallo (2011). Semi-quantitative analysis of the effects of cyclosporine on remyelination following gliotoxic injection in the brainstem. *Arq Neuropsiquiatr*; **69**(2B): 377–383.
[DOI:10.1590/S0004-282X2011000300021] [PubMed:21625769]

Brück, W., R. Pförtner, T. Pham, J. Zhang, L. Hayardeny, V. Piryatinsky, U. K. Hanisch, T. Regen, D. van Rossum, L. Brakelmann, K. Hagemeier, T. Kuhlmann, C. Stadelmann, G. R. John, N. Kramann, and C.

Wegner (2012). Reduced astrocytic NF-κB activation by laquinimod protects from cuprizone-induced demyelination. *Acta Neuropathol.*; **124**(3): 411–424.
[DOI:10.1007/s00401-012-1009-1] [PubMed:22766690]

Cate, H. S., Q. Z. Wu, D. Kemper, D. Merlo, H. X. Wang, K. Fang, G. F. Egan, and T. J. Kilpatrick (2010). Influence of methylprednisolone on magnetic resonance and histological measures during cuprizone-induced demyelination. *Neurosci. Lett.*; **483**(1): 47–52.
[DOI:10.1016/j.neulet.2010.07.060] [PubMed:20674674]

Chari, D. M., C. Zhao, M. R. Kotter, W. F. Blakemore, and R. J. Franklin (2006). Corticosteroids delay remyelination of experimental demyelination in the rodent central nervous system. *J. Neurosci. Res.*; **83**(4): 594–605.
[DOI:10.1002/jnr.20763] [PubMed:16429447]

Clarner, T., A. Parabucki, C. Beyer, and M. Kipp (2011). Corticosteroids impair remyelination in the corpus callosum of cuprizone-treated mice. *J. Neuroendocrinol.*; **23**(7): 601–611.
[DOI:10.1111/j.1365-2826.2011.02140.x] [PubMed:21507085]

Deshmukh, V. A., V. Tardif, C. A. Lyssiotis, C. C. Green, B. Kerman, H. J. Kim, K. Padmanabhan, J. G. Swoboda, I. Ahmad, T. Kondo, F. H. Gage, A. N. Theofilopoulos, B. R. Lawson, P. G. Schultz, and L. L. Lairson (2013). A regenerative approach to the treatment of multiple sclerosis. *Nature*; **502**(7471): 327–332.
[DOI:10.1038/nature12647] [PubMed:24107995]

Döring, A., S. Sloka, L. Lau, M. Mishra, J. van Minnen, X. Zhang, D. Kinniburgh, S. Rivest, and V. W. Yong (2015). Stimulation of monocytes, macrophages, and microglia by amphotericin B and macrophage colony-stimulating factor promotes remyelination. *J. Neurosci.*; **35**(3): 1136–1148.
[DOI:10.1523/JNEUROSCI.1797-14.2015] [PubMed:25609628]

El-Etr, M., M. Rame, C. Boucher, A. M. Ghoumari, N. Kumar, P. Liere, A. Pianos, M. Schumacher, and R. Sitruk-Ware (2015). Progesterone and nestorone promote myelin regeneration in chronic demyelinating lesions of corpus callosum and cerebral cortex. *Glia*; **63**(1): 104–117.
[DOI:10.1002/glia.22736] [PubMed:25092805]

Franco, P. G., L. Silvestroff, E. F. Soto, and J. M. Pasquini (2008). Thyroid hormones promote differentiation of oligodendrocyte progenitor cells and improve remyelination after cuprizone-induced demyelination. *Exp. Neurol.*; **212**(2): 458–467.
[DOI:10.1016/j.expneurol.2008.04.039] [PubMed:18572165]

Gonzalez-Perez, O., R. Romero-Rodriguez, M. Soriano-Navarro, J. M. Garcia-Verdugo, and A. Alvarez-Buylla (2009). Epidermal growth factor induces the progeny of subventricular zone type B cells to migrate and differentiate into oligodendrocytes. *Stem Cells*; **27**(8): 2032–2043.
[DOI:10.1002/stem.119] [PubMed:19544429]

Goudarzvand, M., M. Javan, J. Mirnajafi-Zadeh, S. Mozafari, and T. Tiraihi (2009). De-myelination, cell death and endogenous re-myelination following ethidium bromide induced insult in rat hippocampal formation. *J. Neurochem.*; **110**(Suppl. 1): 1–106, Abstract of the 4th European Society for Neurochemistry Conference on Advances in Molecular Mechanisms of Neurological Disorders, poster 28.
[DOI:10.1111/j.1471-4159.2009.06064_5.x]

Goudarzvand, M., M. Javan, J. Mirnajafi-Zadeh, S. Mozafari, and T. Tiraihi (2010). Vitamins E and D_3 attenuate demyelination and potentiate remyelination processes of hippocampal formation of rats following local injection of ethidium bromide. *Cell. Mol. Neurobiol.*; **30**(2): 289–299.
[DOI:10.1007/s10571-009-9451-x] [PubMed:19768531]

Hagemeyer, N., S. Boretius, C. Ott, A. Von Streitberg, H. Welpinghus, S. Sperling, J. Frahm, M. Simons, P. Ghezzi, and H. Ehrenreich (2012). Erythropoietin attenuates neurological and histological consequences of toxic demyelination in mice. *Mol. Med.*; **18**: 628–635.
[DOI:10.2119/molmed.2011.00457] [PubMed:22396019]

Harsan, L. A., J. Steibel, A. Zaremba, A. Agin, R. Sapin, P. Poulet, B. Guignard, N. Parizel, D. Grucker, N. Boehm, R. H. Miller, and M. S. Ghandour (2008). Recovery from chronic demyelination by thyroid hormone therapy: myelinogenesis induction and assessment by diffusion tensor magnetic resonance imaging. *J. Neurosci.*; **28**(52): 14189–14201.
[DOI:10.1523/JNEUROSCI.4453-08.2008] [PubMed:19109501]

Hirahara, Y., K. I. Matsuda, H. Yamada, A. Saitou, S. Morisaki, K. Takanami, J. M. Boggs, and M. Kawata (2013). G protein-coupled receptor 30 contributes to improved remyelination after cuprizone-induced demyelination. *Glia*; **61**(3): 420–431.
[DOI:10.1002/glia.22445] [PubMed:23281138]

Hu, Y., X. Lee, B. Ji, K. Guckian, D. Apicco, R. B. Pepinsky, R. H. Miller, and S. Mi (2011). Sphingosine 1-phosphate receptor modulator fingolimod (FTY720) does not promote remyelination in vivo. *Mol. Cell. Neurosci.*; **48**(1): 72–81.
[DOI:10.1016/j.mcn.2011.06.007] [PubMed:21740973]

Huang, S. F., Y. Ding, J. W. Ruan, W. Zhang, J. L. Wu, B. He, Y. J. Zhang, Y. Li, and Y. S. Zeng (2011). An experimental electro-acupuncture study in treatment of the rat demyelinated spinal cord injury induced by ethidium bromide. *Neurosci. Res.*; **70**(3): 294–304.
[DOI:10.1016/j.neures.2011.03.010] [PubMed:21470565]

Hussain, R., A. M. Ghoumari, B. Bielecki, J. Steibel, N. Boehm, P. Liere, W. B. Macklin, N. Kumar, R. Habert, S. Mhaouty-Kodja, F. Tronche, R. Sitruk-Ware, M. Schumacher, and M. S. Ghandour (2013). The neural androgen receptor: a therapeutic target for myelin repair in chronic demyelination. *Brain*; **136**(1): 132–146.
[DOI:10.1093/brain/aws284] [PubMed:23365095]

Ibanez, C., S. A. Shields, M. El-Etr, E. E. Baulieu, M. Schumacher, and R. J. Franklin (2004). Systemic progesterone administration results in a partial reversal of the age-associated decline in CNS remyelination following toxin-induced demyelination in male rats. *Neuropathol. Appl. Neurobiol.*; **30**(1): 80–89.
[DOI:10.1046/j.0305-1846.2003.00515.x] [PubMed:14720179]

Iwasa, K., S. Yamamoto, M. Takahashi, S. Suzuki, S. Yagishita, T. Awaji, K. Maruyama, and K. Yoshikawa (2014). Prostaglandin $F_{2\alpha}$ FP receptor inhibitor reduces demyelination and motor dysfunction in a cuprizone-induced multiple sclerosis mouse model. *Prostaglandins Leukot. Essent. Fatty Acids*; **91**(5): 175–182.
[DOI:10.1016/j.plefa.2014.08.004] [PubMed:25224839]

Jean, I. and C. Fressinaud (2003). Spontaneous central nervous system remyelination is not altered in NFH-lacZ transgenic mice after chemical demyelination. *J. Neurosci. Res.*; **73**(1): 54–60.
[DOI:10.1002/jnr.10640] [PubMed:12815708]

Jean, I., C. Lavialle, A. Barthelaix-Pouplard, and C. Fressinaud (2003). Neurotrophin-3 specifically increases mature oligodendrocyte population and enhances remyelination after chemical demyelination of adult rat CNS. *Brain Res.*; **972**(1-2): 110–118.
[DOI:10.1016/S0006-8993(03)02510-1] [PubMed:12711083]

Kalakh, S. and A. Mouihate (2015). The promyelinating properties of androstenediol in gliotoxin-induced demyelination in rat corpus callosum. *Neuropathol. Appl. Neurobiol.*; **41**(7): 964–982.
[DOI:10.1111/nan.12237] [PubMed:25786683]

Kashani, I. R., A. Hedayatpour, P. Pasbakhsh, L. Kafami, N. Atlasi, V. P. Mahabadi, R. Mamoudi, and M. Baazm (2012). 17β-Estradiol enhances the efficacy of adipose-derived mesenchymal stem cells on remyelination in mouse model of multiple sclerosis. *Acta Medica Iranica*; **50**(12): 789–797.
[URL:http://acta.tums.ac.ir/index.php/acta/article/view/3995]

Kashani, I. R., A. Hedayatpour, P. Pasbakhsh, L. Kafami, B. Khallaghi, and F. Malek (2015). Progesterone enhanced remyelination in the mouse corpus callosum after cuprizone induced demyelination. *Iran J Med Sci*; **40**(6): 507–514.
[URL:http://ijms.sums.ac.ir/index.php/IJMS/article/view/191] [PubMed:26538779]

Kashani, I. R., Z. Rajabi, M. Akbari, G. Hassanzadeh, A. Mohseni, M. K. Eramsadati, K. Rafiee, C. Beyer, M. Kipp, and A. Zendedel (2014). Protective effects of melatonin against mitochondrial injury in a mouse model of multiple sclerosis. *Exp Brain Res*; **232**(9): 2835–2846.
[DOI:10.1007/s00221-014-3946-5] [PubMed:24798398]

Kim, H. J., V. E. Miron, D. Dukala, R. L. Proia, S. K. Ludwin, M. Traka, J. P. Antel, and B. Soliven (2011). Neurobiological effects of sphingosine 1-phosphate receptor modulation in the cuprizone model. *FASEB J.*; **25**(5): 1509–1518.
[DOI:10.1096/fj.10-173203] [PubMed:21248243]

Klopfleisch, S., D. Merkler, M. Schmitz, S. Klöppner, M. Schedensack, G. Jeserich, H. H. Althaus, and W. Brück (2008). Negative impact of statins on oligodendrocytes and myelin formation *in vitro* and *in vivo*. *J. Neurosci.*; **28**(50): 13609–13614.
[DOI:10.1523/JNEUROSCI.2765-08.2008] [PubMed:19074034]

Kumar, S., J. C. Biancotti, M. Yamaguchi, and J. de Vellis (2007). Combination of growth factors enhances remyelination in a cuprizone-induced demyelination mouse model. *Neurochem. Res.*; **32**(4-5): 783–797.
[DOI:10.1007/s11064-006-9208-6] [PubMed:17186374]

Lee, K. H., D. H. Yoon, M. A. Chung, J. H. Sohn, H. J. Lee, and B. H. Lee (2010). Neuroprotective effects of mexiletine on motor evoked potentials in demyelinated rat spinal cords. *Neurosci. Res.*; **67**(1): 59–64.
[DOI:10.1016/j.neures.2010.01.004] [PubMed:20096736]

Li, C., L. Xiao, X. Liu, W. Yang, W. Shen, C. Hu, G. Yang, and C. He (2013). A functional role of NMDA receptor in regulating the differentiation of oligodendrocyte precursor cells and remyelination. *Glia*; **61**(5): 732–749.
[DOI:10.1002/glia.22469] [PubMed:23440860]

Li, J., C. A. Ghiani, J. Y. Kim, A. Liu, J. Sandoval, J. DeVellis, and P. Casaccia-Bonnefil (2008). Inhibition of p53 transcriptional activity: a potential target for future development of therapeutic strategies for primary demyelination. *J. Neurosci.*; **28**(24): 6118–6127.
[DOI:10.1523/JNEUROSCI.0184-08.2008] [PubMed:18550754]

Li, W. W., A. Setzu, C. Zhao, and R. J. Franklin (2005). Minocycline-mediated inhibition of microglia activation impairs oligodendrocyte progenitor cell responses and remyelination in a non-immune model of demyelination. *J. Neuroimmunol.*; **158**(1-2): 58–66.
[DOI:10.1016/j.jneuroim.2004.08.011] [PubMed:15589038]

Li, Y., Y. Zhang, W. Han, F. Hu, Y. Qian, and Q. Chen (2013). TRO19622 promotes myelin repair in a rat model of demyelination. *Int. J. Neurosci.*; **123**(11): 810–822.
[DOI:10.3109/00207454.2013.804523] [PubMed:23668883]

Li, Z., Z. Y. Fang, L. Xiong, and X. L. Huang (2010). Spinal cord injury-induced astrocyte migration and glial scar formation: effects of magnetic stimulation frequency. *Indian J. Biochem. Biophys.*; **47**(6): 359–363.
[URL:http://nopr.niscair.res.in/handle/123456789/10859] [PubMed:21355419]

Liang, M., Y. Chen, L. Zhang, L. Li, G. Chen, and L. Yin (2015). Epimedium flavonoids ameliorate neuropathological changes and increases IGF-1 expression in C57BL/6 mice exposed to cuprizone. *Neurochem. Res.*; **40**(3): 492–500.
[DOI:10.1007/s11064-014-1490-0] [PubMed:25663299]

Magalon, K., C. Zimmer, M. Cayre, J. Khaldi, C. Bourbon, I. Robles, G. Tardif, A. Viola, R. M. Pruss, T. Bordet, and P. Durbec (2012). Olesoxime accelerates myelination and promotes repair in models of demyelination. *Ann. Neurol.*; **71**(2): 213–226.
[DOI:10.1002/ana.22593] [PubMed:22367994]

Maheshwari, A., K. Janssens, J. Bogie, C. Van Den Haute, T. Struys, I. Lambrichts, V. Baekelandt, P. Stinissen, J. J. Hendriks, H. Slaets, and N. Hellings (2013). Local overexpression of interleukin-11 in the central nervous system limits demyelination and enhances remyelination. *Mediators Inflamm.*; **2013**: 685317.
[DOI:10.1155/2013/685317] [PubMed:23818742]

Marriott, M. P., B. Emery, H. S. Cate, M. D. Binder, D. Kemper, Q. Wu, S. Kolbe, I. R. Gordon, H. Wang, G. Egan, S. Murray, H. Butzkueven, and T. J. Kilpatrick (2008). Leukemia inhibitory factor signaling modulates both central nervous system demyelination and myelin repair. *Glia*; **56**(6): 686–698. [DOI:10.1002/glia.20646] [PubMed:18293407]

Mazzanti, C. M., R. Spanevello, M. Ahmed, L. B. Pereira, J. F. Gonçalves, M. Corrêa, R. Schmatz, N. Stefanello, D. B. Leal, A. Mazzanti, A. T. Ramos, T. B. Martins, C. C. Danesi, D. L. Graça, V. M. Morsch, and M. R. Schetinger (2009). Pre-treatment with ebselen and vitamin E modulate acetylcholinesterase activity: interaction with demyelinating agents. *Int. J. Dev. Neurosci.*; **27**(1): 73–80. [DOI:10.1016/j.ijdevneu.2008.09.005] [PubMed:18930802]

McKay, J. S., W. F. Blakemore, and R. J. Franklin (1997). The effects of the growth factor-antagonist, trapidil, on remyelination in the CNS. *Neuropathol. Appl. Neurobiol.*; **23**(1): 50–58. [DOI:10.1111/j.1365-2990.1997.tb01185.x] [PubMed:9061690]

McKay, J. S., W. F. Blakemore, and R. J. Franklin (1998). Trapidil-mediated inhibition of CNS remyelination results from reduced numbers and impaired differentiation of oligodendrocytes. *Neuropathol. Appl. Neurobiol.*; **24**(6): 498–506. [DOI:10.1046/j.1365-2990.1998.00148.x] [PubMed:9888160]

Mi, S., R. H. Miller, W. Tang, X. Lee, B. Hu, W. Wu, Y. Zhang, C. B. Shields, Y. Zhang, S. Miklasz, D. Shea, J. Mason, R. J. Franklin, B. Ji, Z. Shao, A. Chédotal, F. Bernard, A. Roulois, J. Xu, V. Jung, and B. Pepinsky (2009). Promotion of central nervous system remyelination by induced differentiation of oligodendrocyte precursor cells. *Ann. Neurol.*; **65**(3): 304–315. [DOI:10.1002/ana.21581] [PubMed:19334062]

Millet, V., C. P. Moiola, J. M. Pasquini, E. F. Soto, and L. A. Pasquini (2009). Partial inhibition of proteasome activity enhances remyelination after cuprizone-induced demyelination. *Exp. Neurol.*; **217**(2): 282–296. [DOI:10.1016/j.expneurol.2009.03.005] [PubMed:19303006]

Miron, V. E., S. P. Zehntner, T. Kuhlmann, S. K. Ludwin, T. Owens, T. E. Kennedy, B. J. Bedell, and J. P. Antel (2009). Statin therapy inhibits remyelination in the central nervous system. *Am. J. Pathol.*; **174**(5): 1880–1890. [DOI:10.2353/ajpath.2009.080947] [PubMed:19349355]

Moharregh-Khiabani, D., A. Blank, T. Skripuletz, E. Miller, A. Kotsiari, V. Gudi, and M. Stangel (2010). Effects of fumaric acids on cuprizone induced central nervous system de- and remyelination in the mouse. *PLoS ONE*; **5**(7): e11769. [DOI:10.1371/journal.pone.0011769] [PubMed:20668697]

Morita, S., K. Tatsumi, M. Makinodan, H. Okuda, T. Kishimoto, and A. Wanaka (2014). Geissoschizine methyl ether, an alkaloid from the Uncaria hook, improves remyelination after cuprizone-induced demyelination in medial prefrontal cortex of adult mice. *Neurochem. Res.*; **39**(1): 59–67. [DOI:10.1007/s11064-013-1190-1] [PubMed:24190599]

Mueller, A. M., A. Nassery, H. Conlon, X. Liu, E. Jun, B. H. Yoon, M. Cristofanilli, and S. A. Sadiq (2013). Effects of intraventricular methotrexate administration on Cuprizone-induced demyelination in mice. *Front Mol Neurosci*; **6**: 34. [DOI:10.3389/fnmol.2013.00034] [PubMed:24137109]

Muramatsu, R., M. Kuroda, K. Matoba, H. Lin, C. Takahashi, Y. Koyama, and T. Yamashita (2015). Prostacyclin prevents pericyte loss and demyelination induced by lysophosphatidylcholine in the central nervous system. *J. Biol. Chem.*; **290**(18): 11515–11525. [DOI:10.1074/jbc.M114.587253] [PubMed:25795781]

Najm, F. J., M. Madhavan, A. Zaremba, E. Shick, R. T. Karl, D. C. Factor, T. E. Miller, Z. S. Nevin, C. Kantor, A. Sargent, K. L. Quick, D. M. Schlatzer, H. Tang, R. Papoian, K. R. Brimacombe, M. Shen, M. B. Boxer, A. Jadhav, A. P. Robinson, J. R. Podojil, S. D. Miller, R. H. Miller, and P. J. Tesar (2015). Drug-based modulation of endogenous stem cells promotes functional remyelination *in vivo*. *Nature*; **522**(7555): 216–

220.
[DOI:10.1038/nature14335] [PubMed:25896324]

Nunes, A. K., C. Rapôso, R. L. Luna, M. A. Cruz-Höfling, and C. A. Peixoto (2012). Sildenafil (Viagra®) down regulates cytokines and prevents demyelination in a cuprizone-induced MS mouse model. *Cytokine*; **60**(2): 540–551.
[DOI:10.1016/j.cyto.2012.06.011] [PubMed:22749439]

Nystad, A. E., S. Wergeland, L. Aksnes, K. M. Myhr, L. Bø, and Ø. Torkildsen (2014). Effect of high-dose 1.25 dihydroxyvitamin D_3 on remyelination in the cuprizone model. *APMIS*; **122**(12): 1178–1186.
[DOI:10.1111/apm.12281] [PubMed:24862867]

Palumbo, S., C. D. Toscano, L. Parente, R. Weigert, and F. Bosetti (2012). The cyclooxygenase-2 pathway via the PGE_2, EP2 receptor contributes to oligodendrocytes apoptosis in cuprizone-induced demyelination. *J. Neurochem.*; **121**(3): 418–427.
[DOI:10.1111/j.1471-4159.2011.07363.x] [PubMed:21699540]

Patel, R., S. Moore, D. K. Crawford, G. Hannsun, M. V. Sasidhar, K. Tan, D. Molaie, and S. K. Tiwari-Woodruff (2013). Attenuation of corpus callosum axon myelination and remyelination in the absence of circulating sex hormones. *Brain Pathol.*; **23**(4): 462–475.
[DOI:10.1111/bpa.12029] [PubMed:23311751]

Pavelko, K. D., B. G. van Engelen, and M. Rodriguez (1998). Acceleration in the rate of CNS remyelination in lysolecithin-induced demyelination. *J. Neurosci.*; **18**(7): 2498–2505.
[URL:http://www.jneurosci.org/content/18/7/2498] [PubMed:9502810]

Penderis, J., R. H. Woodruff, A. Lakatos, W. W. Li, M. D. Dunning, C. Zhao, M. Marchionni, and R. J. Franklin (2003). Increasing local levels of neuregulin (glial growth factor-2) by direct infusion into areas of demyelination does not alter remyelination in the rat CNS. *Eur. J. Neurosci.*; **18**(8): 2253–2264.
[DOI:10.1046/j.1460-9568.2003.02969.x] [PubMed:14622186]

Pepinsky, R. B., Z. Shao, B. Ji, Q. Wang, G. Meng, L. Walus, X. Lee, Y. Hu, C. Graff, E. Garber, W. Meier, and S. Mi (2011). Exposure levels of anti-LINGO-1 Li81 antibody in the central nervous system and dose-efficacy relationships in rat spinal cord remyelination models after systemic administration. *J. Pharmacol. Exp. Ther.*; **339**(2): 519–529.
[DOI:10.1124/jpet.111.183483] [PubMed:21807883]

Pourabdolhossein, F., S. Mozafari, G. Morvan-Dubois, J. Mirnajafi-Zadeh, A. Lopez-Juarez, J. Pierre-Simons, B. A. Demeneix, and M. Javan (2014). Nogo receptor inhibition enhances functional recovery following lysolecithin-induced demyelination in mouse optic chiasm. *PLoS ONE*; **9**(9): e106378.
[DOI:10.1371/journal.pone.0106378] [PubMed:25184636]

Preston, M., X. Gong, W. Su, S. G. Matsumoto, F. Banine, C. Winkler, S. Foster, R. Xing, J. Struve, J. Dean, B. Baggenstoss, P. H. Weigel, T. J. Montine, S. A. Back, and L. S. Sherman (2013). Digestion products of the PH20 hyaluronidase inhibit remyelination. *Ann. Neurol.*; **73**(2): 266–280.
[DOI:10.1002/ana.23788] [PubMed:23463525]

Rosato Siri, M. V., M. E. Badaracco, and J. M. Pasquini (2013). Glatiramer promotes oligodendroglial cell maturation in a cuprizone-induced demyelination model. *Neurochemistry International*; **63**(1): 10–24.
[DOI:10.1016/j.neuint.2013.04.008]

Sabo, J. K., T. D. Aumann, T. J. Kilpatrick, and H. S. Cate (2013). Investigation of sequential growth factor delivery during cuprizone challenge in mice aimed to enhance oligodendrogliogenesis and myelin repair. *PLoS ONE*; **8**(5): e63415.
[DOI:10.1371/journal.pone.0063415] [PubMed:23650566]

Sabo, J. K., T. D. Aumann, D. Merlo, T. J. Kilpatrick, and H. S. Cate (2011). Remyelination is altered by bone morphogenic protein signaling in demyelinated lesions. *J. Neurosci.*; **31**(12): 4504–4510.
[DOI:10.1523/JNEUROSCI.5859-10.2011] [PubMed:21430151]

Seiwa, C., M. Yamamoto, K. Tanaka, M. Fukutake, T. Ueki, S. Takeda, R. Sakai, A. Ishige, K. Watanabe, M. Akita, T. Yagi, K. Tanaka, and H. Asou (2007). Restoration of FcRγ/Fyn signaling repairs central nervous system demyelination. *J. Neurosci. Res.*; **85**(5): 954–966.
[DOI:10.1002/jnr.21196] [PubMed:17290413]

Shen, S., J. Sandoval, V. A. Swiss, J. Li, J. Dupree, R. J. Franklin, and P. Casaccia-Bonnefil (2008). Age-dependent epigenetic control of differentiation inhibitors is critical for remyelination efficiency. *Nat. Neurosci.*; **11**(9): 1024–1034.
[DOI:10.1038/nn.2172] [PubMed:19160500]

Sherafat, M. A., M. Heibatollahi, S. Mongabadi, F. Moradi, M. Javan, and A. Ahmadiani (2012). Electromagnetic field stimulation potentiates endogenous myelin repair by recruiting subventricular neural stem cells in an experimental model of white matter demyelination. *J. Mol. Neurosci.*; **48**(1): 144–153.
[DOI:10.1007/s12031-012-9791-8] [PubMed:22588976]

Silvestroff, L., S. Bartucci, J. Pasquini, and P. Franco (2012). Cuprizone-induced demyelination in the rat cerebral cortex and thyroid hormone effects on cortical remyelination. *Exp. Neurol.*; **235**(1): 357–367.
[DOI:10.1016/j.expneurol.2012.02.018] [PubMed:22421533]

Skihar, V., C. Silva, A. Chojnacki, A. Döring, W. B. Stallcup, S. Weiss, and V. W. Yong (2009). Promoting oligodendrogenesis and myelin repair using the multiple sclerosis medication glatiramer acetate. *Proc. Natl. Acad. Sci. U.S.A.*; **106**(42): 17992–17997.
[DOI:10.1073/pnas.0909607106] [PubMed:19815532]

Skripuletz, T., A. Manzel, K. Gropengiesser, N. Schäfer, V. Gudi, V. Singh, L. Salinas Tejedor, S. Jörg, A. Hammer, E. Voss, F. Vulinovic, D. Degen, R. Wolf, D. H. Lee, R. Pul, D. Moharregh-Khiabani, W. Baumgärtner, R. Gold, R. A. Linker, and M. Stangel (2015). Pivotal role of choline metabolites in remyelination. *Brain*; **138**(2): 398–413.
[DOI:10.1093/brain/awu358] [PubMed:25524711]

Skripuletz, T., E. Miller, D. Moharregh-Khiabani, A. Blank, R. Pul, V. Gudi, C. Trebst, and M. Stangel (2010). Beneficial effects of minocycline on cuprizone induced cortical demyelination. *Neurochem. Res.*; **35**(9): 1422–1433.
[DOI:10.1007/s11064-010-0202-7] [PubMed:20544279]

Slowik, A., T. Schmidt, C. Beyer, S. Amor, T. Clarner, and M. Kipp (2015). The sphingosine 1-phosphate receptor agonist FTY720 is neuroprotective after cuprizone-induced CNS demyelination. *Br. J. Pharmacol.*; **172**(1): 80–92.
[DOI:10.1111/bph.12938] [PubMed:25220526]

Smith, P. M. and R. J. Franklin (2001). The effect of immunosuppressive protocols on spontaneous CNS remyelination following toxin-induced demyelination. *J. Neuroimmunol.*; **119**(2): 261–268.
[DOI:10.1016/S0165-5728(01)00396-4] [PubMed:11585629]

Sohn, J., V. Selvaraj, K. Wakayama, L. Orosco, E. Lee, S. E. Crawford, F. Guo, J. Lang, M. Horiuchi, K. Zarbalis, T. Itoh, W. Deng, and D. Pleasure (2012). PEDF is a novel oligodendrogenic morphogen acting on the adult SVZ and corpus callosum. *J. Neurosci.*; **32**(35): 12152–12164.
[DOI:10.1523/JNEUROSCI.0628-12.2012] [PubMed:22933798]

Spanevello, R., C. M. Mazzanti, R. Schmatz, M. Bagatini, N. Stefanello, M. Correa, R. Kaizer, P. Maldonado, A. Mazzanti, D. L. Graça, T. B. Martins, C. Danesi, V. M. Morsch, and M. R. Schetinger (2009). Effect of vitamin E on ectonucleotidase activities in synaptosomes and platelets and parameters of oxidative stress in rats experimentally demyelinated. *Brain Res. Bull.*; **80**(1-2): 45–51.
[DOI:10.1016/j.brainresbull.2009.05.015] [PubMed:19463911]

Takahashi, C., R. Muramatsu, H. Fujimura, H. Mochizuki, and T. Yamashita (2013). Prostacyclin promotes oligodendrocyte precursor recruitment and remyelination after spinal cord demyelination. *Cell Death Dis*; **4**: e795.
[DOI:10.1038/cddis.2013.335] [PubMed:24030147]

Talbott, J. F., Q. Cao, J. Bertram, M. Nkansah, R. L. Benton, E. Lavik, and S. R. Whittemore (2007). CNTF promotes the survival and differentiation of adult spinal cord-derived oligodendrocyte precursor cells *in vitro* but fails to promote remyelination *in vivo*. *Exp. Neurol.*; **204**(1): 485–489.
[DOI:10.1016/j.expneurol.2006.12.013] [PubMed:17274982]

Torkildsen, Ø., L. A. Brunborg, F. Thorsen, S. J. Mørk, M. Stangel, K. M. Myhr, and L. Bø (2009). Effects of dietary intervention on MRI activity, de- and remyelination in the cuprizone model for demyelination. *Exp. Neurol.*; **215**(1): 160–166.
[DOI:10.1016/j.expneurol.2008.09.026] [PubMed:19000674]

Tsiperson, V., X. Li, G. J. Schwartz, C. S. Raine, and B. Shafit-Zagardo (2010). GAS6 enhances repair following cuprizone-induced demyelination. *PLoS ONE*; **5**(12): e15748.
[DOI:10.1371/journal.pone.0015748] [PubMed:21203420]

Vakilzadeh, G., F. Khodagholi, T. Ghadiri, A. Ghaemi, F. Noorbakhsh, M. Sharifzadeh, and A. Gorji (2016). The effect of melatonin on behavioral, molecular, and histopathological changes in cuprizone model of demyelination. *Mol. Neurobiol.*; **53**(7): 4675–4684.
[DOI:10.1007/s12035-015-9404-y] [PubMed:26310973]

Veto, S., P. Acs, J. Bauer, H. Lassmann, Z. Berente, G. Setalo, G. Borgulya, B. Sumegi, S. Komoly, F. Gallyas, and Z. Illes (2010). Inhibiting poly(ADP-ribose) polymerase: a potential therapy against oligodendrocyte death. *Brain*; **133**(3): 822–834.
[DOI:10.1093/brain/awp337] [PubMed:20157013]

Wang, W. W., L. Lu, T. H. Bao, H. M. Zhang, J. Yuan, W. Miao, S. F. Wang, and Z. C. Xiao (2016). Scutellarin alleviates behavioral deficits in a mouse model of multiple sclerosis, possibly through protecting neural stem cells. *J. Mol. Neurosci.*; **58**(2): 210–220.
[DOI:10.1007/s12031-015-0660-0] [PubMed:26514969]

Wergeland, S., Ø. Torkildsen, K. M. Myhr, L. Aksnes, S. J. Mørk, and L. Bø (2011). Dietary vitamin D3 supplements reduce demyelination in the cuprizone model. *PLoS ONE*; **6**(10): e26262.
[DOI:10.1371/journal.pone.0026262] [PubMed:22028844]

Williams, J. L., J. R. Patel, B. P. Daniels, and R. S. Klein (2014). Targeting CXCR7/ACKR3 as a therapeutic strategy to promote remyelination in the adult central nervous system. *J. Exp. Med.*; **211**(5): 791–799.
[DOI:10.1084/jem.20131224] [PubMed:24733828]

Wu, Y., Q. Huang, X. Liu, and X. Wei (2015). Dl-3-n-butylphthalide is effective for demyelination: a case-combined study. *Clin Neurol Neurosurg*; **137**: 83–88.
[DOI:10.1016/j.clineuro.2015.06.024] [PubMed:26164676]

Xiao, L., H. Xu, Y. Zhang, Z. Wei, J. He, W. Jiang, X. Li, L. E. Dyck, R. M. Devon, Y. Deng, and X. M. Li (2008). Quetiapine facilitates oligodendrocyte development and prevents mice from myelin breakdown and behavioral changes. *Mol. Psychiatry*; **13**(7): 697–708.
[DOI:10.1038/sj.mp.4002064] [PubMed:17684494]

Yamamoto, S., M. Gotoh, Y. Kawamura, K. Yamashina, S. Yagishita, T. Awaji, M. Tanaka, K. Maruyama, K. Murakami-Murofushi, and K. Yoshikawa (2014). Cyclic phosphatidic acid treatment suppress cuprizone-induced demyelination and motor dysfunction in mice. *Eur. J. Pharmacol.*; **741**: 17–24.
[DOI:10.1016/j.ejphar.2014.07.040] [PubMed:25084219]

Yoshikawa, K., S. Palumbo, C. D. Toscano, and F. Bosetti (2011). Inhibition of 5-lipoxygenase activity in mice during cuprizone-induced demyelination attenuates neuroinflammation, motor dysfunction and axonal damage. *Prostaglandins Leukot. Essent. Fatty Acids*; **85**(1): 43–52.
[DOI:10.1016/j.plefa.2011.04.022] [PubMed:21555210]

Zhang, H., Y. Zhang, H. Xu, L. Wang, A. Adilijiang, J. Wang, K. Hartle, Z. Zhang, D. Zhang, Q. Tan, J. Kong, Q. Huang, and X. M. Li (2014). Olanzapine ameliorates neuropathological changes and increases IGF-1 expression in frontal cortex of C57BL/6 mice exposed to cuprizone. *Psychiatry Res*; **216**(3): 438–445.
[DOI:10.1016/j.psychres.2014.02.019] [PubMed:24613202]

Zhang, J., Z. G. Zhang, Y. Li, M. Lu, Y. Zhang, S. B. Elias, and M. Chopp (2016). Thymosin beta4 promotes oligodendrogenesis in the demyelinating central nervous system. *Neurobiol. Dis.*; **88**: 85–95. [DOI:10.1016/j.nbd.2016.01.010] [PubMed:26805386]

Zhang, M., X. L. Zhan, Z. Y. Ma, X. S. Chen, Q. Y. Cai, and Z. X. Yao (2015). Thyroid hormone alleviates demyelination induced by cuprizone through its role in remyelination during the remission period. *Exp. Biol. Med. (Maywood)*; **240**(9): 1183–1196. [DOI:10.1177/1535370214565975] [PubMed:25577802]

Zhang, Y., H. Xu, W. Jiang, L. Xiao, B. Yan, J. He, Y. Wang, X. Bi, X. Li, J. Kong, and X. M. Li (2008). Quetiapine alleviates the cuprizone-induced white matter pathology in the brain of C57BL/6 mouse. *Schizophr. Res.*; **106**(2-3): 182–191. [DOI:10.1016/j.schres.2008.09.013] [PubMed:18938062]

Zhang, Y., H. Zhang, L. Wang, W. Jiang, H. Xu, L. Xiao, X. Bi, J. Wang, S. Zhu, R. Zhang, J. He, Q. Tan, D. Zhang, J. Kong, and X. M. Li (2012). Quetiapine enhances oligodendrocyte regeneration and myelin repair after cuprizone-induced demyelination. *Schizophr. Res.*; **138**(1): 8–17. [DOI:10.1016/j.schres.2012.04.006] [PubMed:22555017]

Zhang, Y., Y. P. Zhang, B. Pepinsky, G. Huang, L. B. Shields, C. B. Shields, and S. Mi (2015). Inhibition of LINGO-1 promotes functional recovery after experimental spinal cord demyelination. *Exp. Neurol.*; **266**: 68–73. [DOI:10.1016/j.expneurol.2015.02.006] [PubMed:25681574]
